# Supplementary material for: The acquisition of molecular drivers in pediatric therapy-related myeloid neoplasms
Source: Nat Commun. 2021 Feb 12;12:985. doi: 10.1038/s41467-021-21255-8 (PMC7880998; doi:10.1038/s41467-021-21255-8)
Supplement: Supplementary file 1 — Supplementary Information [file 41467_2021_21255_MOESM1_ESM.pdf]

Supplementary Figures

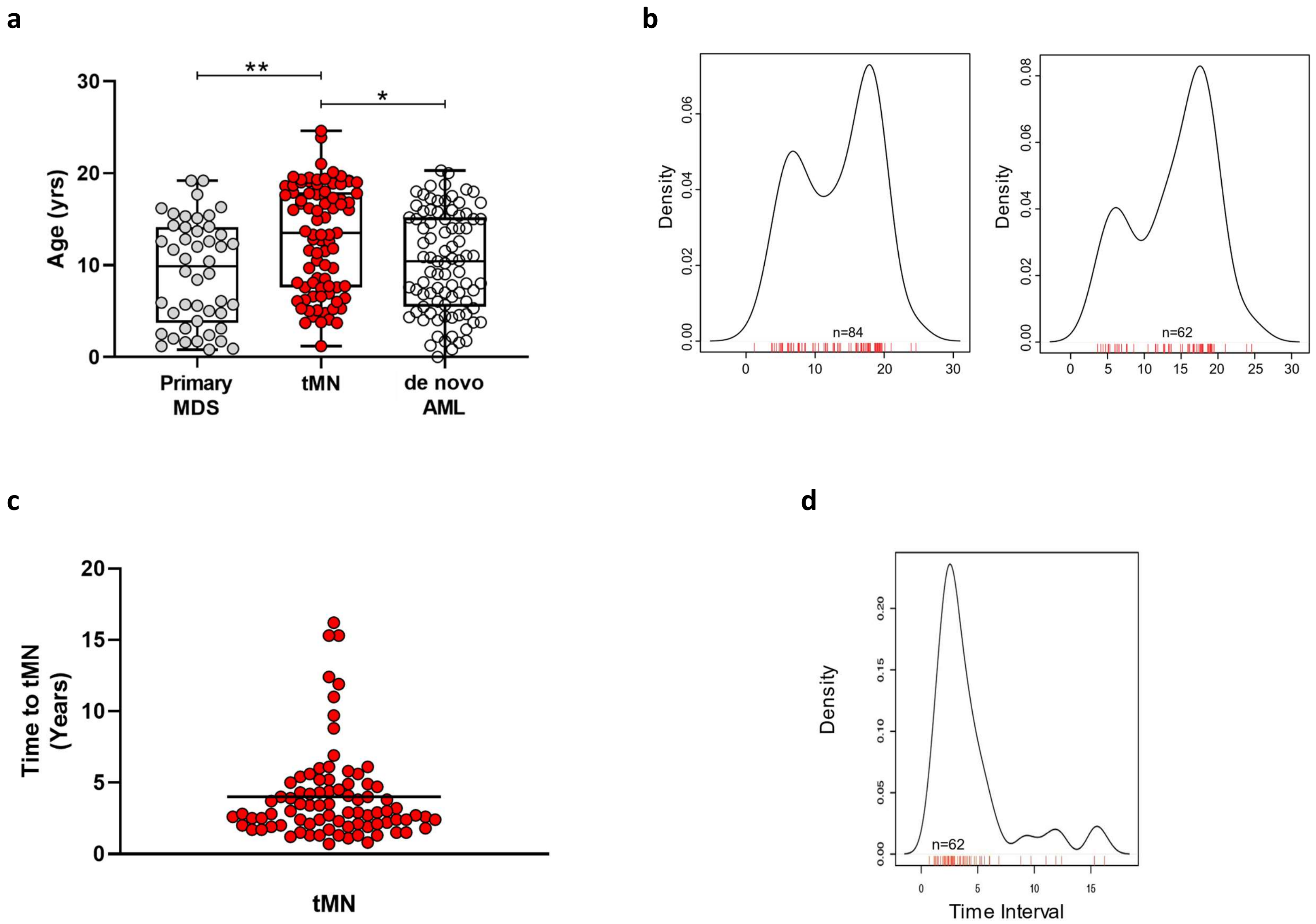

e

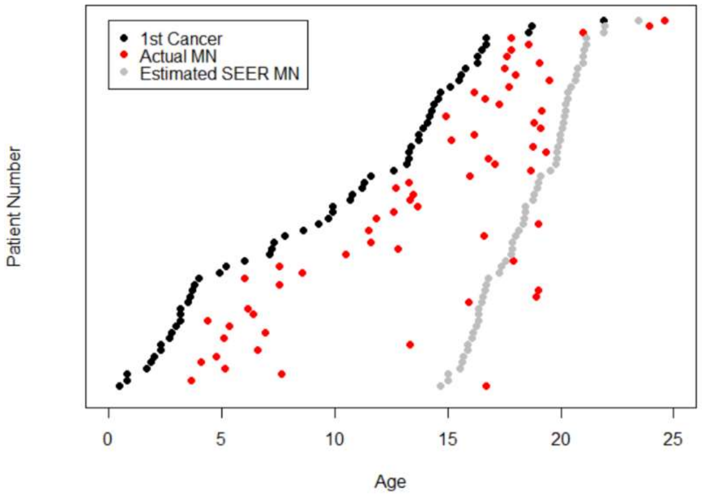

Supplementary Figure 1. a) The median age of the cohort (n=84) at the time of tMN diagnosis was 13.6 years (range: 1.2-24.6 yrs) which is significantly greater than that of pediatric primary MDS (median: 9.9 years, **: p=0.0002) and pediatric *de novo* core-binding factor AML (median: 10.4 years, *: p=0.0007)[1,2]. The Wilcoxon-Mann-Whitney non-parametric, two-tailed test was used in (a). Boxes delineate the upper and lower quartiles and the black bar indicates the median. b) Density plots showing that both the entire tMN cohort (left; n=84) and the tumor/normal sub-cohort (right; n=62) are bimodal populations (Dip Test p=0.05), however, there was no significant association between age and disease-related death (p=0.77) or transplant-related death (p=0.49). c) The time to tMN for the full cohort (n=84); line indicates a mean of 4.0 years. d) Density plot showing that the time interval between initial diagnosis and tMN in the tumor/normal sub-cohort (n=62) is not bimodal (Dip Test p=0.99) and there was no significant association with outcome (disease-related death; p=0.72, transplant-related death; p=0.77). In panels b & d the red hash marks along the x-axis indicate the data point for each case. e) Estimated age of myeloid neoplasm based on the age of first cancer and SEER age-specific incidence rates for AML. Each row of three points represents the age of first cancer diagnosis (black dot), the actual age of MN diagnosis (red dot), and the estimated median age of MN diagnosis based on age of first cancer diagnosis from SEER data[3] (gray dot). Patients are sorted by age of first diagnosis. Fifty-five of 62 patients developed a myeloid neoplasm earlier than estimated from SEER incidence data (88%; 95% CI: 78.1%-95.3%, p = 2.43×10^{-10}, two-tailed sign-test).

a

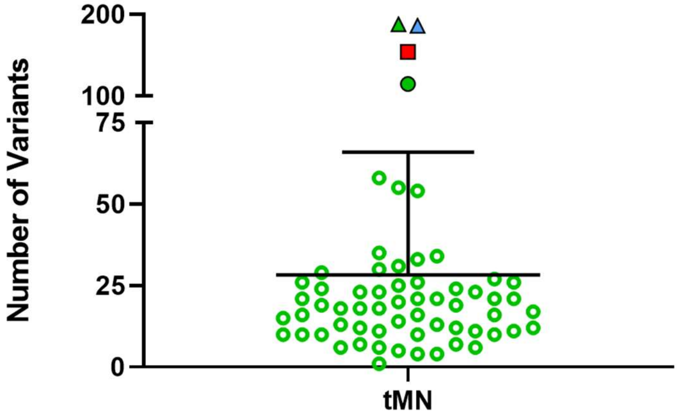

b

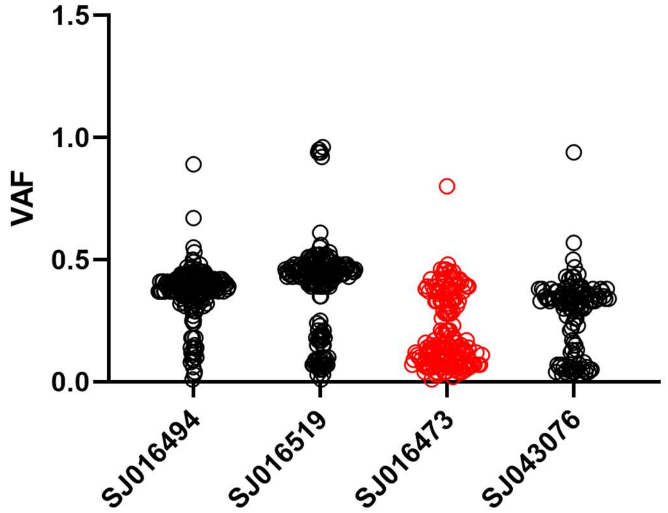

Supplementary Figure 2. a) Total number of somatic variants per patient in 62 tumor/normal pairs (includes the following mutation types: silent, nonsense, frameshift, indels, splice site, ITD, RNA coding gene, 3' and 5' UTR). The 4 hypermutated cases are highlighted; triangles denote *PMS2* mutations; green=somatic, blue=germline, the red square denotes the presence of a mosaic *TP53* mutation, and the green filled circle indicates a somatic *TP53* mutation, and green outlined circles denote non-hypermutated cases. A hypermutated case was defined as having a mutation burden >2 standard deviations (SD) from the mean (mean=28; SD=37.7; mean + 2(SD)=104). Interestingly, both patients with *PMS2* mutations exhibit the relapse MMR signature mutational signature and other studies also suggest that both somatic and germline MMR gene mutations have similar pathogenic effects[4]. b) VAF plot showing all validated somatic mutations for the 4 hypermutated cases (SJ016494, n=188; SJ016519, n=186; SJ016473, n=154; SJ043076, n=115). SJ016473 (red) shows that the hypermutation status appears to be driven by variants with VAFs <0.2, and the corresponding driver alteration (e.g., a mismatch repair gene) could have escaped detection due to limited depth.

a

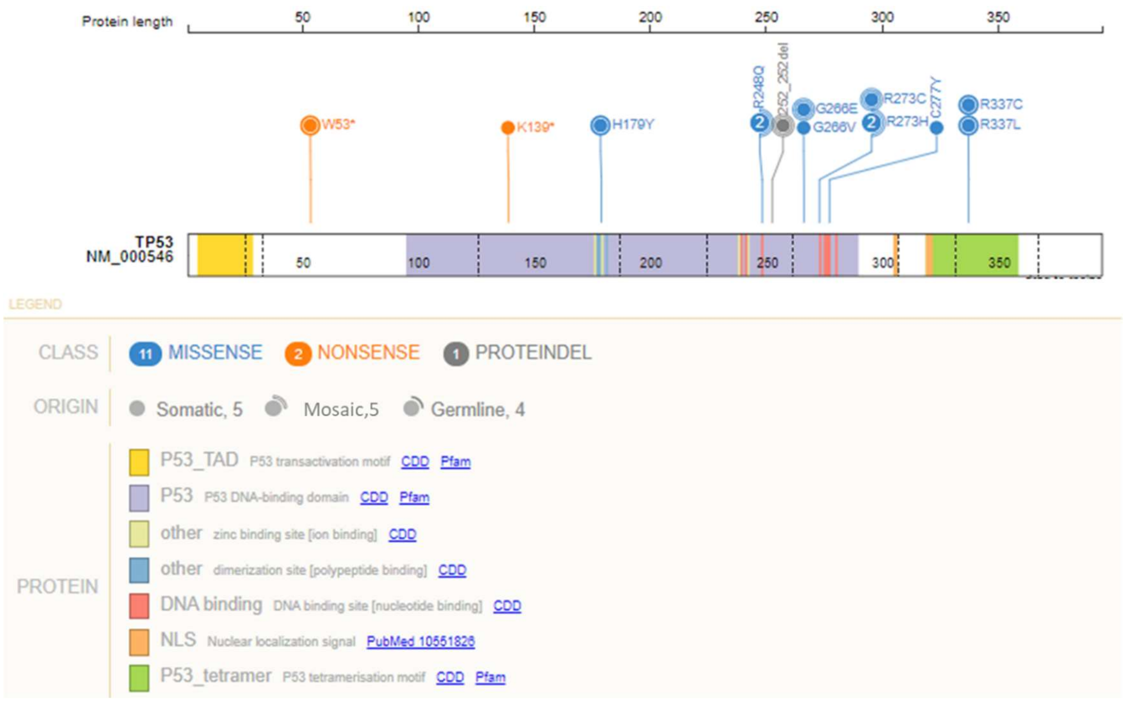

Protein length
TP53
NM_000546
W53*
K139*
H179Y
R248Q
252_252del
G266E
G266V
R273C
R273H
C277Y
R337C
R337L
LEGEND
CLASS
11 MISSENSE
2 NONSENSE
1 PROTEINDEL
ORIGIN
Somatic, 5
Mosaic,5
Germline, 4
PROTEIN
P53_TAD P53 transactivation motif CDD Pfam
P53 P53 DNA-binding domain CDD Pfam
other zinc binding site [ion binding] CDD
other dimerization site [polypeptide binding] CDD
DNA binding DNA binding site [nucleotide binding] CDD
NLS Nuclear localization signal PubMed 10551826
P53_tetramer P53 tetramerisation motif CDD Pfam

b

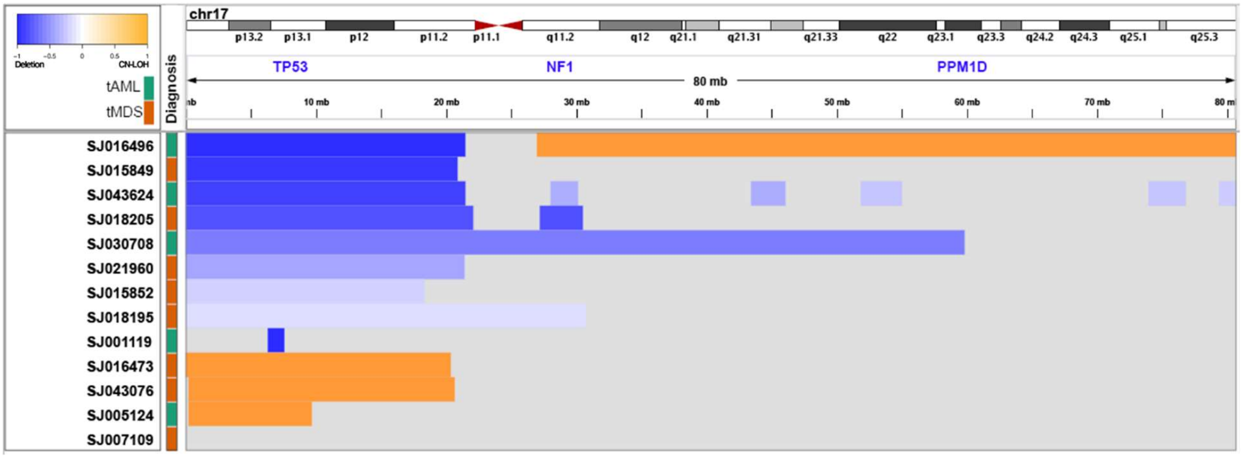

Deletion
CN-LOH
tAML
tMDS
Diagnosis
chr17
TP53
NF1
PPM1D
80 mb
SJ016496
SJ015849
SJ043624
SJ018205
SJ030708
SJ021960
SJ015852
SJ018195
SJ001119
SJ016473
SJ043076
SJ005124
SJ007109

c

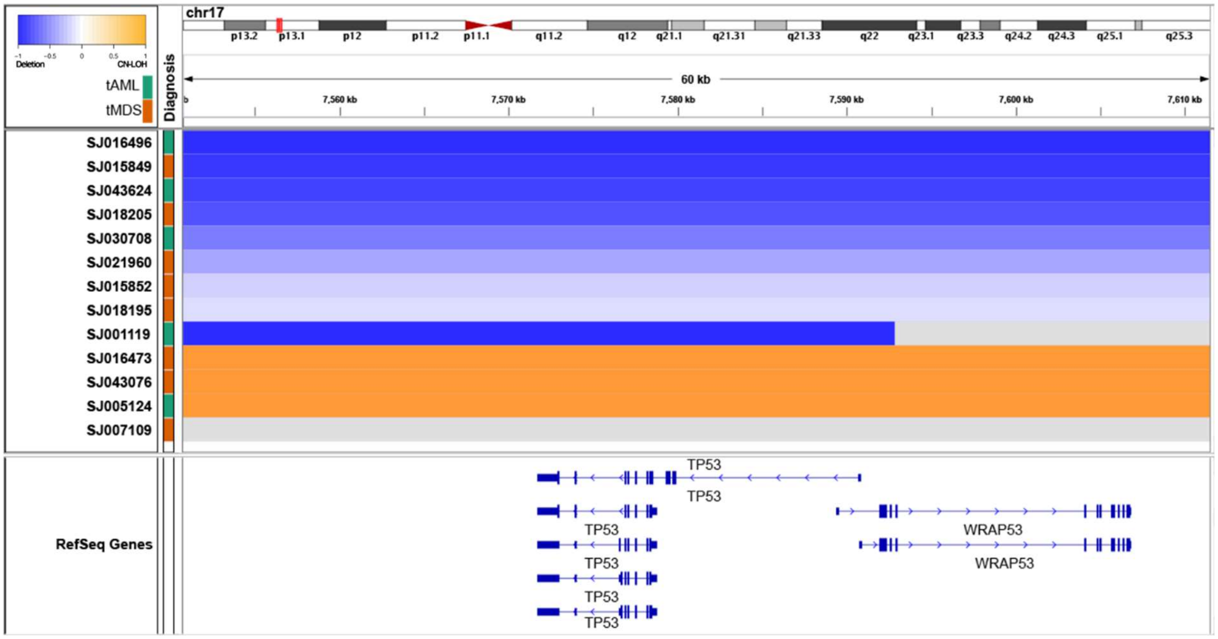
Deletion
CN-LOH
tAML
tMDS
Diagnosis
chr17
p13.2
p13.1
p12
p11.2
p11.1
q11.2
q12
q21.1
q21.31
q21.33
q22
q23.1
q23.3
q24.2
q24.3
q25.1
q25.3
60 kb
7,560 kb
7,570 kb
7,580 kb
7,590 kb
7,600 kb
7,610 kb
SJ016496
SJ015849
SJ043624
SJ018205
SJ030708
SJ021960
SJ015852
SJ018195
SJ001119
SJ016473
SJ043076
SJ005124
SJ007109
RefSeq Genes
TP53
TP53
TP53
TP53
TP53
TP53
WRAP53
WRAP53

d

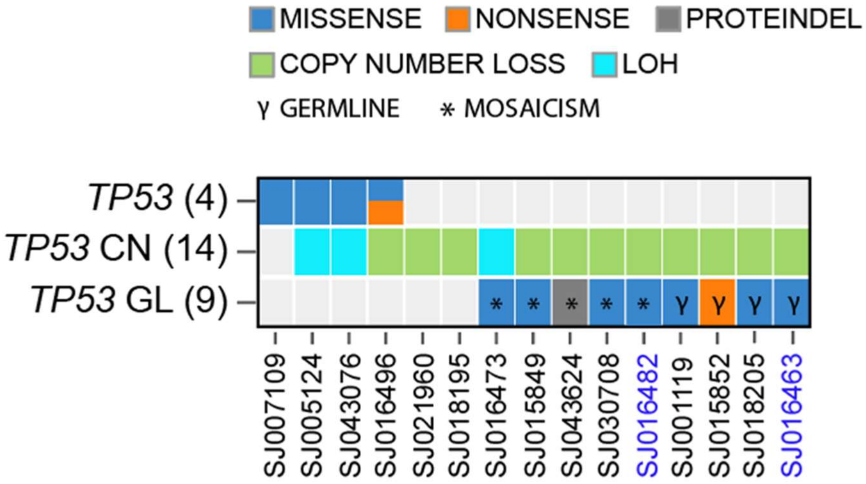

e

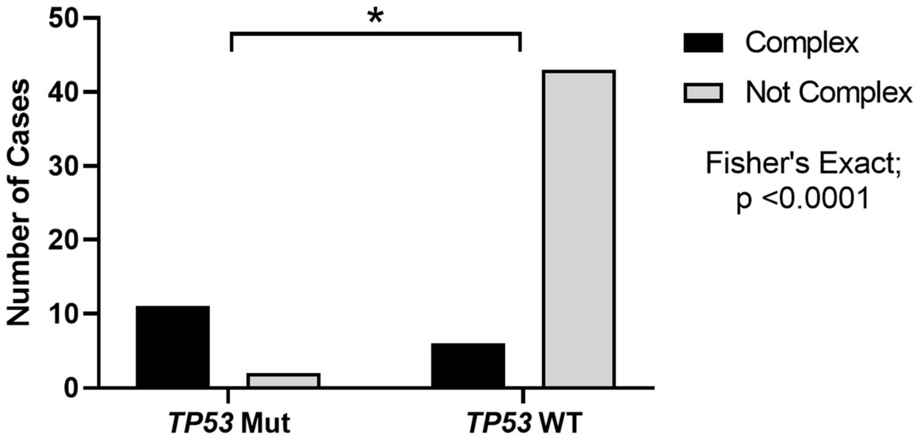

Supplementary Figure 3. a) *TP53* protein domain architecture indicating location and frequency of the somatic, mosaic, and germline *TP53* variants present in the pediatric tMN cohort (14 variants in 13 of 84 cases). b) Integrated Genome Viewer (IGV) plot showing copy number changes involving chromosome 17 for each patient with tumor/normal pairs (13 of 62). Of note, SJMDS007109 has a *TP53* mutation but without a chromosome 17 copy number alteration. Locations of notable gene locations are below chromosome ideogram in blue font. c) IGV plot showing copy number changes involving the *TP53* locus (indicated by the red box on the chromosome 17 ideogram) for each patient with tumor/normal pairs (13 of 62). NOTE: for IGV plots shades of blue denote deletions and orange denotes CN-LOH. d) Heatmap showing the 15 patients with *TP53* alterations. The top row shows the somatic mutations, middle row demonstrates those cases with copy number (CN) alterations, and germline (GL) alterations are on the bottom row. Normal only cases are shown with blue font; note: the *TP53* CN data for these two cases were obtained from clinical data (tumor karyotype) rather than WES as in the remainder of cases shown. Note that SJ021960 and SJ018195 only have chromosome 17 deletions but no somatic SNVs or Indels. e) Contingency bar graph showing a statistically significant (Fisher's exact test, two tailed) enrichment of complex cytogenetics within the group with *TP53* alterations; n=62 biologically independent samples.

a

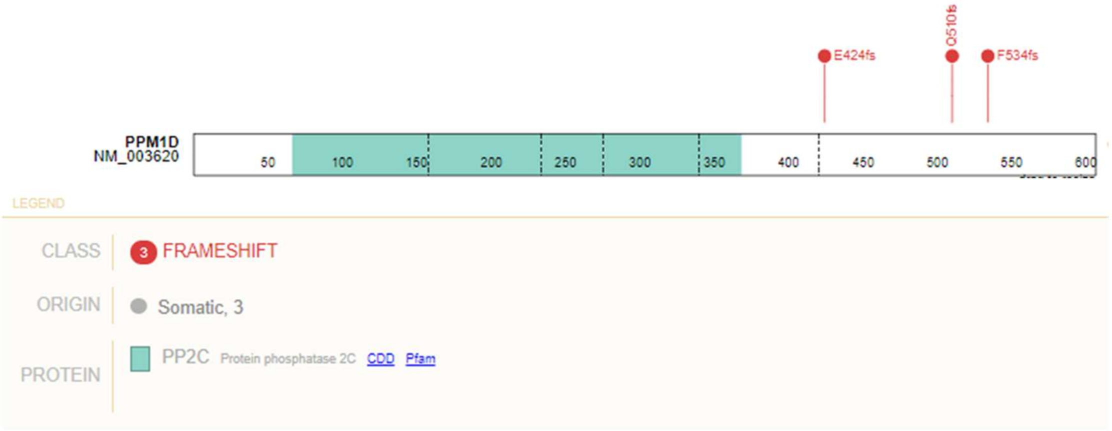

b

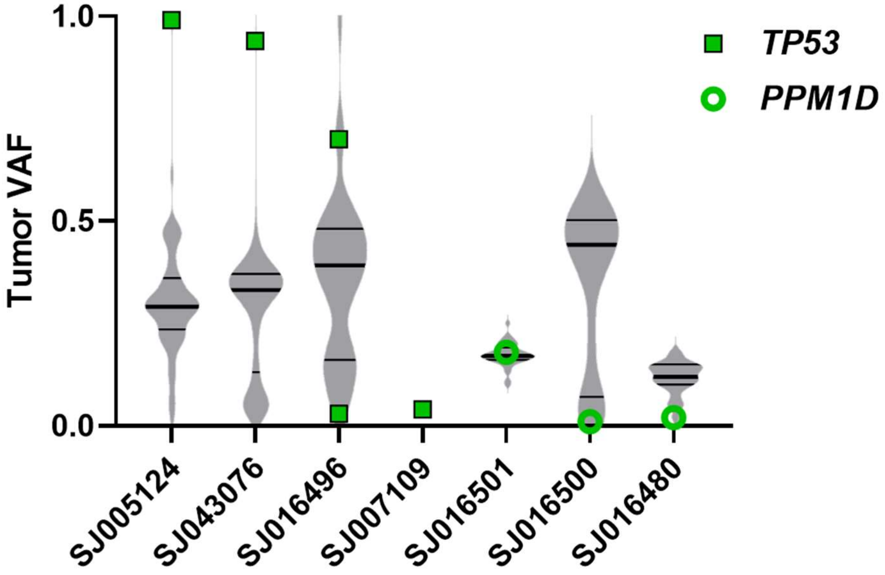

Supplementary Figure 4. a) *PPM1D* protein domain architecture indicating location of the 3 somatic *PPM1D* variants present in the pediatric tMN cohort (3 of 62). b) Tumor VAF plot showing somatic *PPM1D* VAFs (green open circles) relative to the somatic *TP53* VAFs (green closed squares) in other patients. Violin plots represent the range of VAFs for all somatic variants in that case (SJ005124, n=54; SJ043076, n=115; SJ016496, n=35; SJ007109, n=1; SJ016501, n=34; SJ016500, n=15; SJ016480, n=23). Black bars indicate the median and upper and lower quartiles.

a

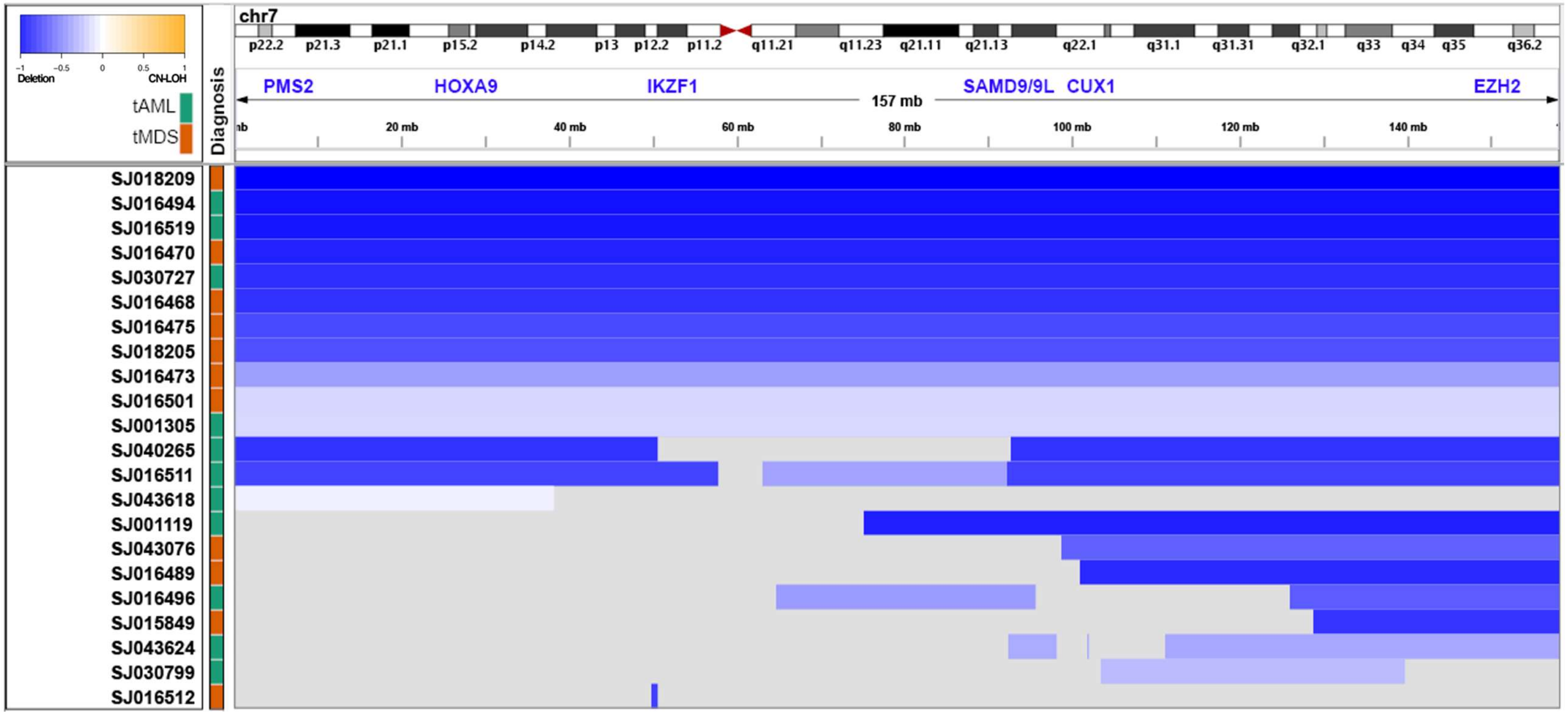

chr7
p22.2
p21.3
p21.1
p15.2
p14.2
p13
p12.2
p11.2
q11.21
q11.23
q21.11
q21.13
q22.1
q31.1
q31.31
q32.1
q33
q34
q35
q36.2
PMS2
HOXA9
IKZF1
SAMD9/9L
CUX1
EZH2
157 mb
20 mb
40 mb
60 mb
80 mb
100 mb
120 mb
140 mb
Deletion
CN-LOH
tAML
tMDS
Diagnosis
SJ018209
SJ016494
SJ016519
SJ016470
SJ030727
SJ016468
SJ016475
SJ018205
SJ016473
SJ016501
SJ001305
SJ040265
SJ016511
SJ043618
SJ001119
SJ043076
SJ016489
SJ016496
SJ015849
SJ043624
SJ030799
SJ016512

b

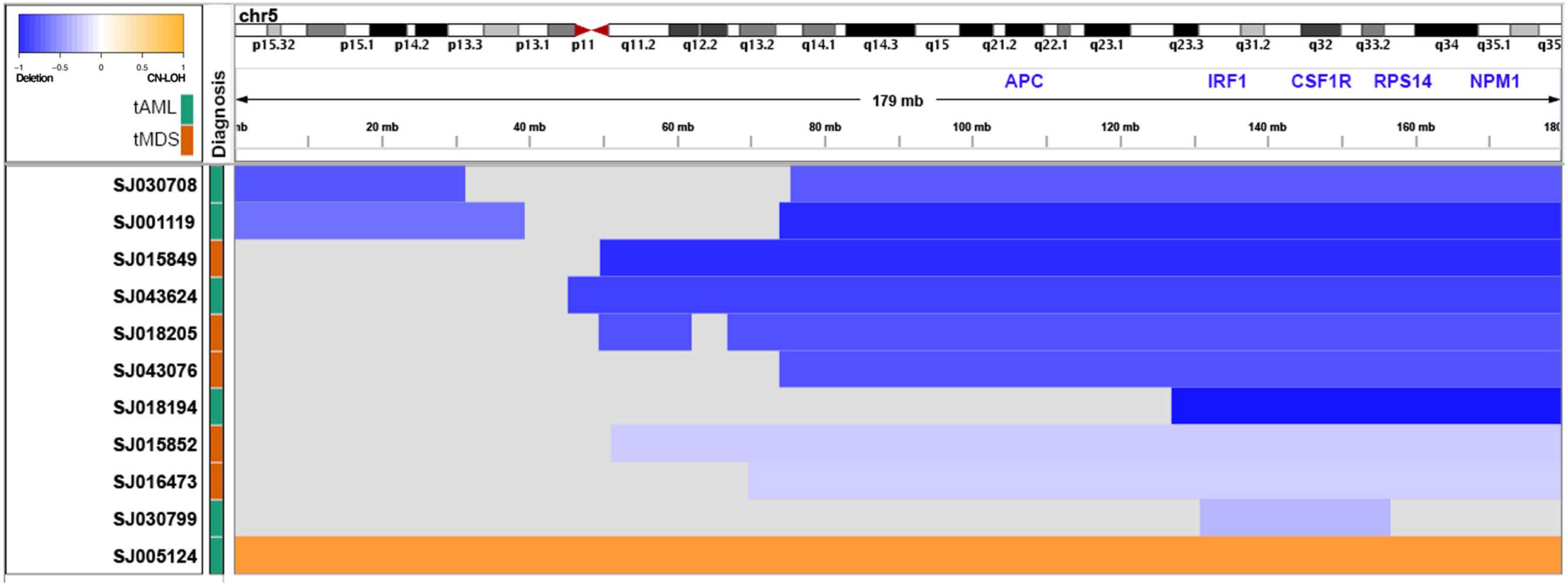

chr5
Deletion
CN-LOH
tAML
tMDS
Diagnosis
179 mb
APC
IRF1
CSF1R
RPS14
NPM1
SJ030708
SJ001119
SJ015849
SJ043624
SJ018205
SJ043076
SJ018194
SJ015852
SJ016473
SJ030799
SJ005124

c

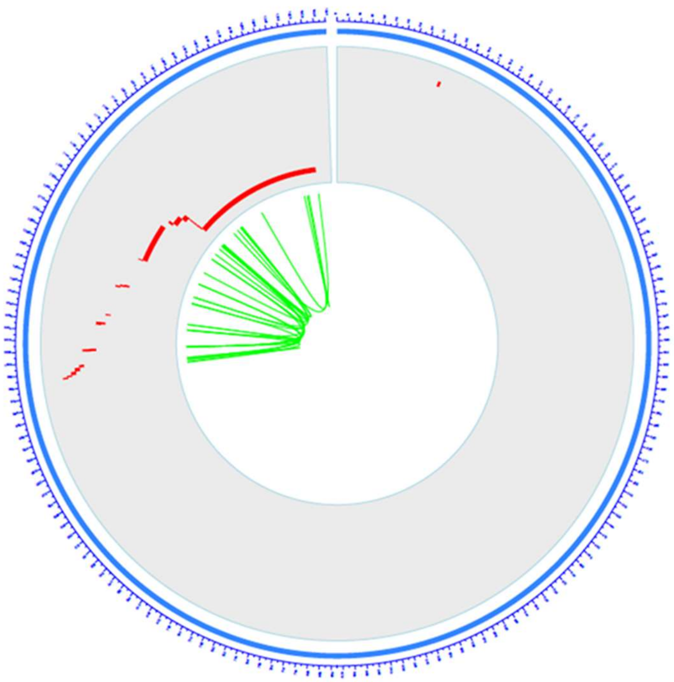

Chromosome 11

Supplementary Figure 5. a) IGV plot showing copy number changes involving chromosome 7 for each patient with tumor/normal pairs (22 of 62). b) IGV plot showing copy number changes involving chromosome 5 for each patient with tumor/normal pairs (11 of 62). NOTE: for IGV plots shades of blue denote deletions and orange denotes CN-LOH. Locations of notable gene locations are below chromosome ideogram in blue font. c) Circos plot showing only chromosome 11 and a large number of rearrangements and limited number of copy-number states in this localized region suggestive of chromothripsis (SJ030708).

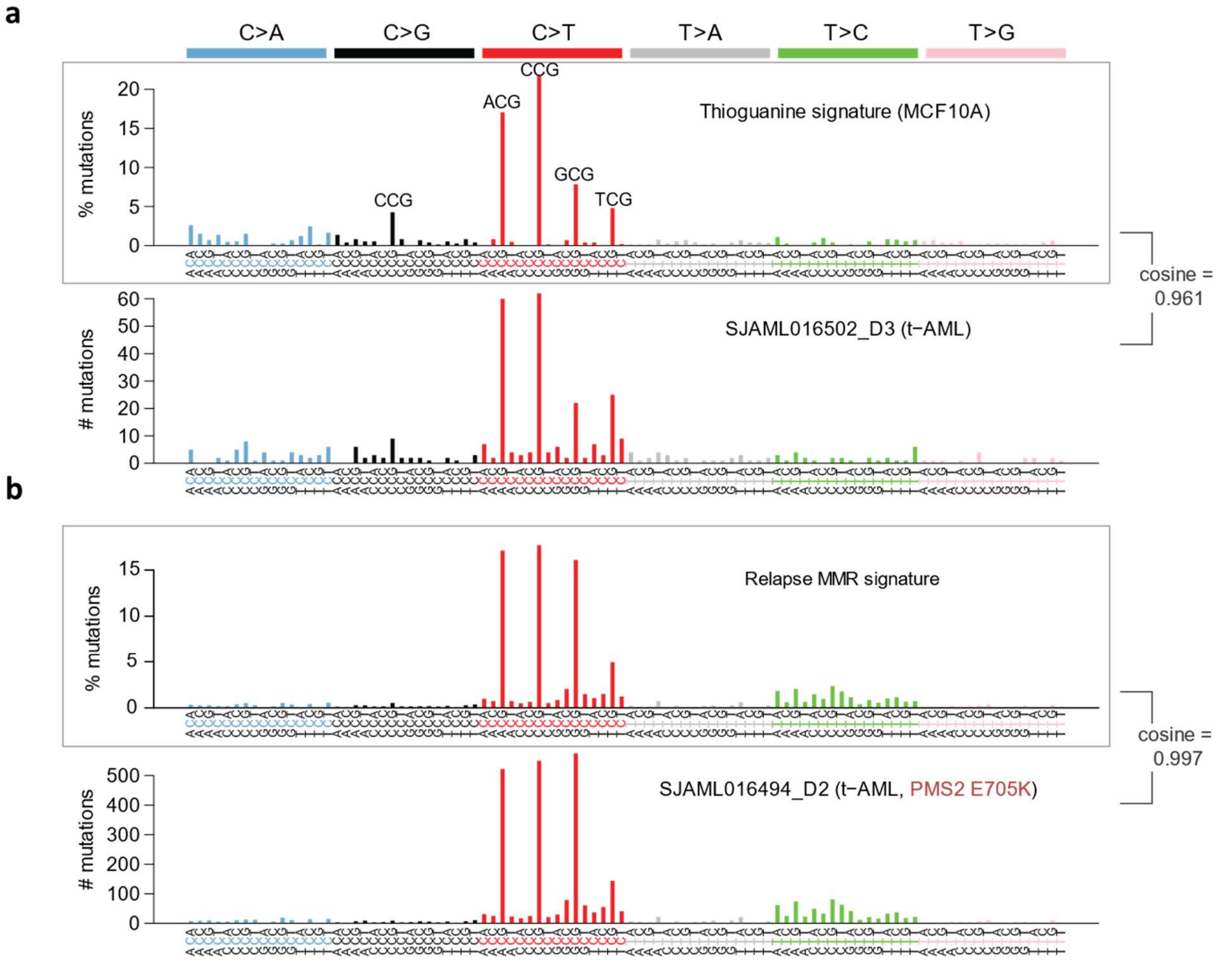

Supplementary Figure 6. a) Mutational spectra of the thioguanine signature as discovered in the MCF10A cell line[5] as compared to (cosine similarity = 0.961) an example case with an initial diagnosis of ALL and known to have been treated with thiopurines. b) Mutational spectra of the relapse MMR signature extracted from the 16 WGS samples as compared to (cosine similarity 0.997) an example case with a somatic pathogenic *PMS2* mutation and an initial diagnosis of ALL and subsequent therapy with thiopurines.

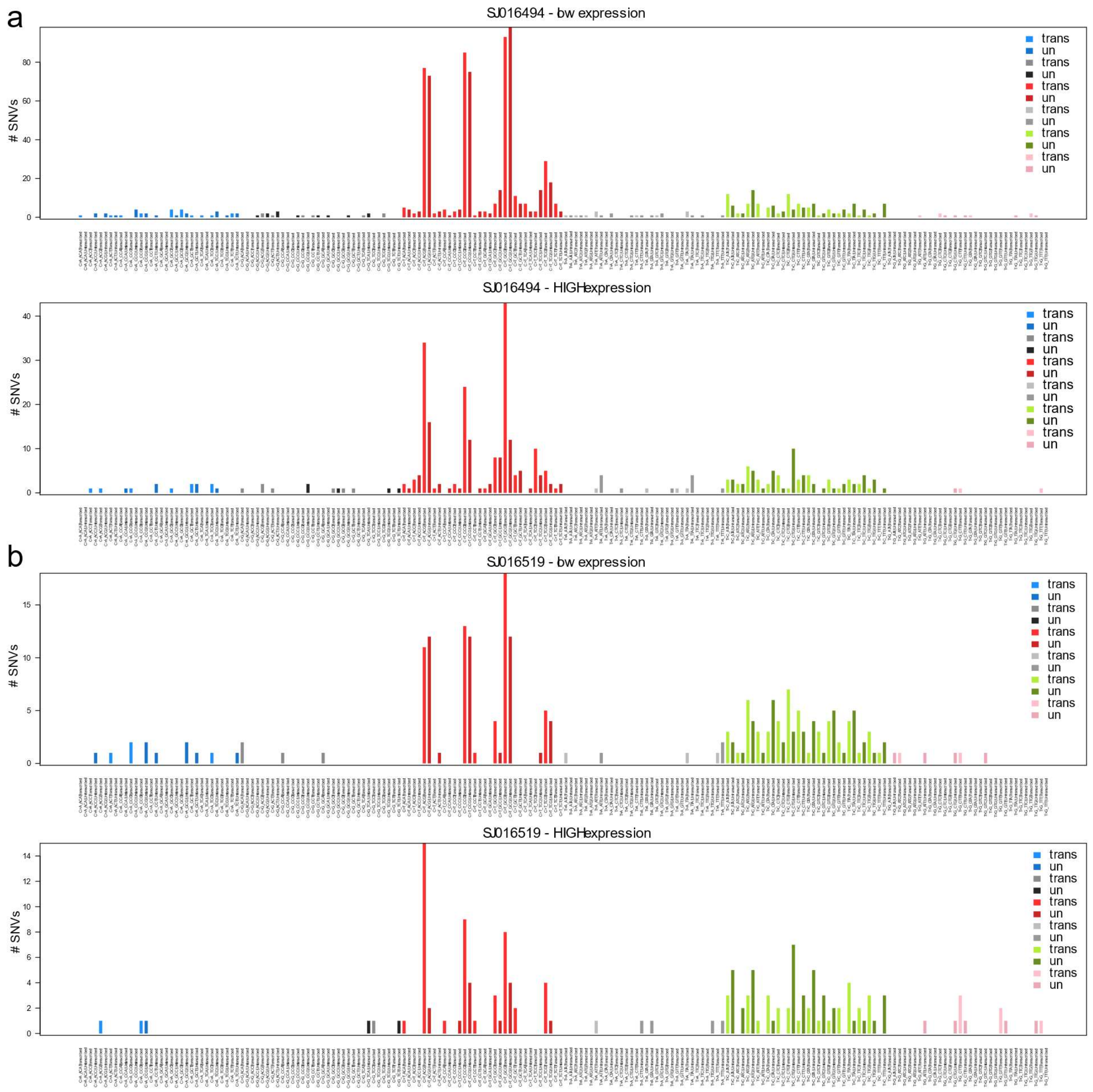

Supplementary Figure 7. Mutation spectrum of SNVs in highly-expressed gene bodies (top 25% of expression) vs. lowly-expressed (bottom 75%) for each of 2 patients with *PMS2* mutations (SJ016494 (a) & SJ016519 (b)) and the relapse MMR signature, showing strand bias in highly expressed genes, indicating the similarities in strand bias to the thiopurine signature and the difference between the relapse MMR signature and COSMIC signature 1 which has no strand bias.

a

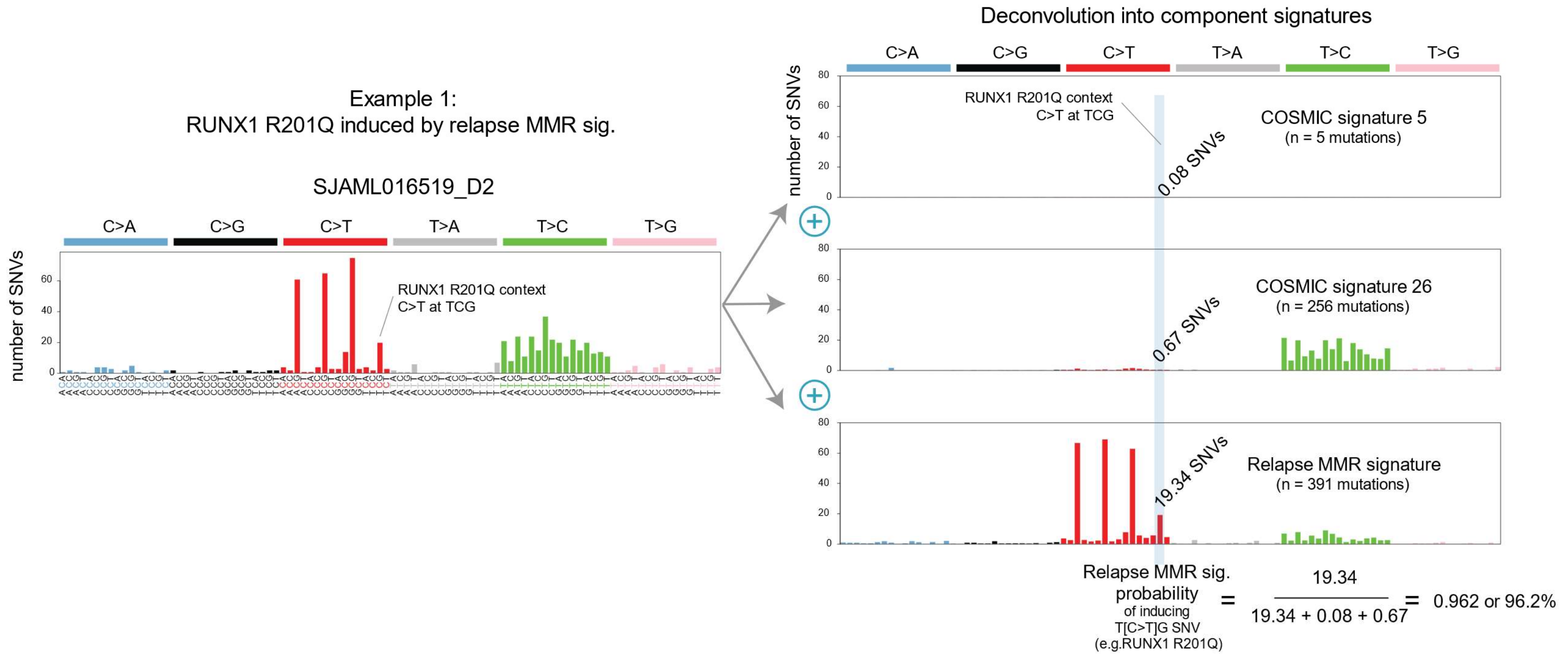
Example 1:
RUNX1 R201Q induced by relapse MMR sig.
SJAML016519_D2
C>A
C>G
C>T
T>A
T>C
T>G
number of SNVs
RUNX1 R201Q context
C>T at TCG
Deconvolution into component signatures
COSMIC signature 5
(n = 5 mutations)
0.08 SNVs
COSMIC signature 26
(n = 256 mutations)
0.67 SNVs
Relapse MMR signature
(n = 391 mutations)
19.34 SNVs
Relapse MMR sig. probability of inducing T[C>T]G SNV (e.g.RUNX1 R201Q)
= 19.34 / (19.34 + 0.08 + 0.67) = 0.962 or 96.2%

b

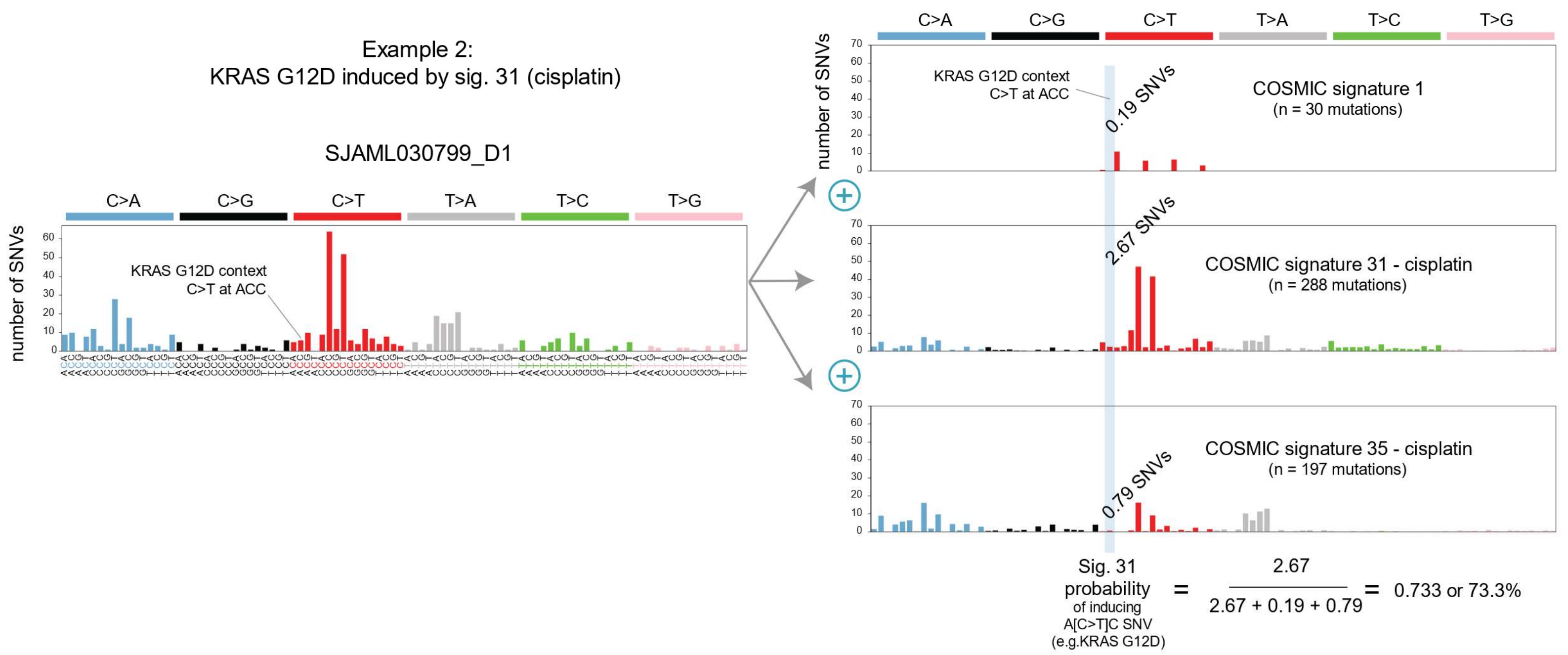

Example 2:
KRAS G12D induced by sig. 31 (cisplatin)
SJAML030799_D1
C>A
C>G
C>T
T>A
T>C
T>G
number of SNVs
KRAS G12D context
C>T at ACC
COSMIC signature 1
(n = 30 mutations)
0.19 SNVs
2.67 SNVs
COSMIC signature 31 - cisplatin
(n = 288 mutations)
0.79 SNVs
COSMIC signature 35 - cisplatin
(n = 197 mutations)
Sig. 31
probability
of inducing
A[C>T]C SNV
(e.g.KRAS G12D)
= 2.67 / (2.67 + 0.19 + 0.79) = 0.733 or 73.3%

c

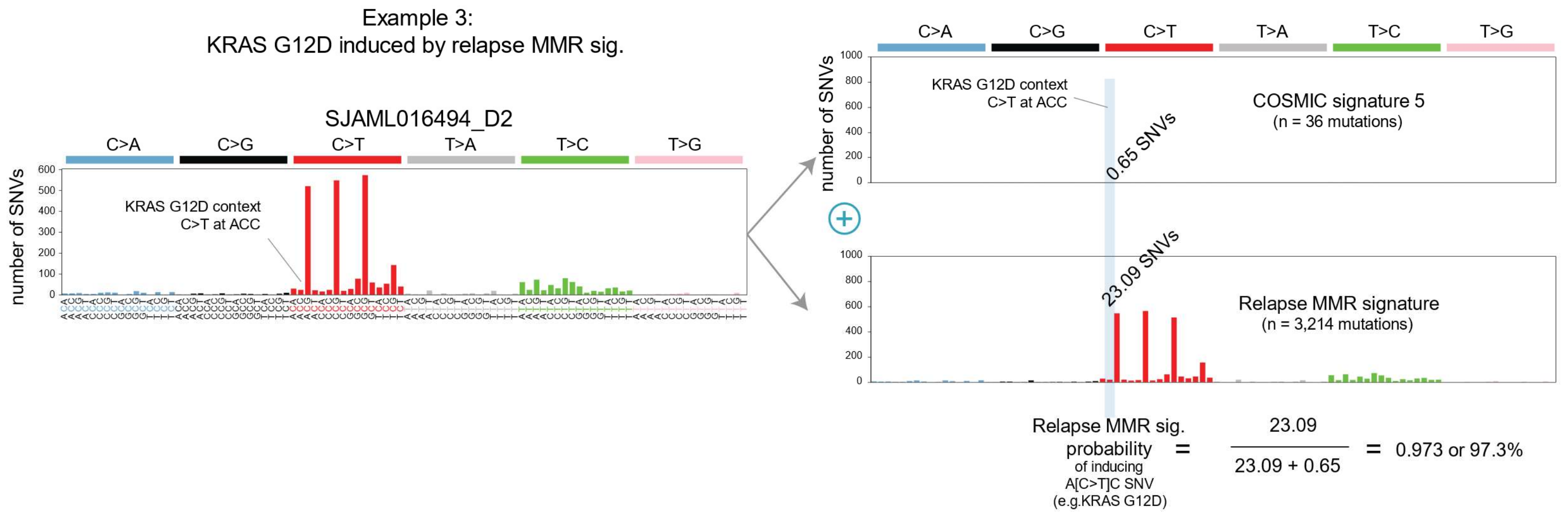

Supplementary Figure 8. Example probability calculations to determine the mutational processes likely causing driver mutations. (a-c) Plots at left show mutational spectra of individual samples based on WGS. Somatic SNVs are divided into 96 categories based on the transition or transversion (top) and the trinucleotide context (light blue shaded bar) surrounding the mutated position (bottom). Y-axis represents number of somatic SNVs falling into each category. Right shows the deconvolution of each sample into three (a, b) or two (c) signatures using SigProfiler, with cosine similarities of 0.98 or above for each sample shown when comparing the actual spectra to the spectra reconstructed by a combination of signatures. The number of mutations predicted to have been caused by each signature are indicated in text (e.g. “n = 256 mutations”), and the numbers of SNVs at the mutation channel corresponding to the driver mutation of interest (e.g. “0.08 SNVs”) were determined by multiplying the total number of mutations caused by the signature by the proportion of mutations caused by the signature at that channel. The probability calculation that each driver SNV was caused by the signature of interest is shown at bottom right. The *KRAS* G12D mutations in (b) and (c) were likely caused by different signatures since the two samples harbored different sets of signatures.

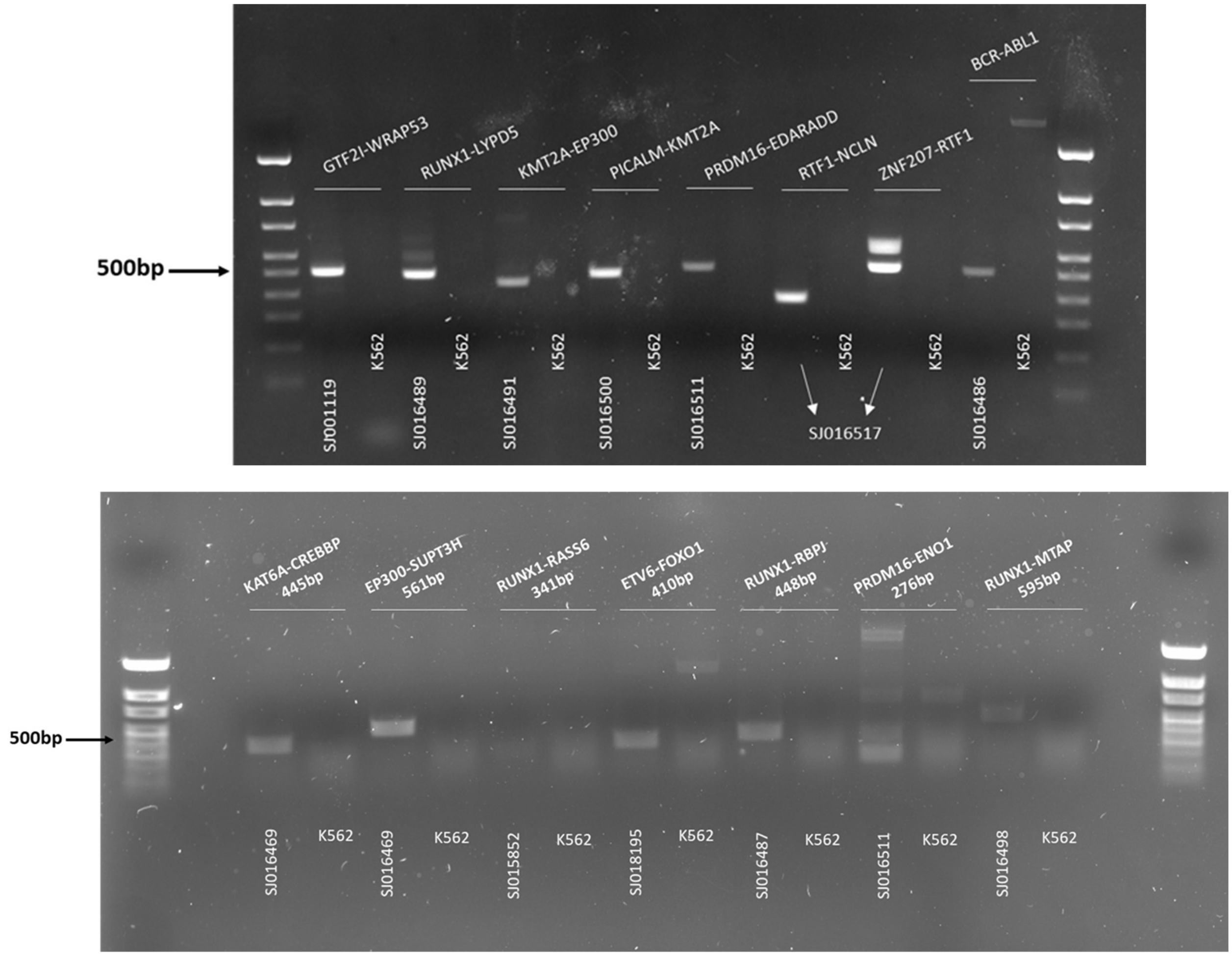

Supplementary Figure 9. RT-PCR validation of selected fusions identified by RNA-Seq. K562 cell line RNA was used as a negative control. NOTE: *RUNX1-RASS6* and *RUNX1-RBPJ* are not in-frame fusions. Based on manual review the *PRDM16-EDARADD* is most likely the *PRDM16-ENO1.* RT-PCR validation was performed once for each fusion given limited patient material.

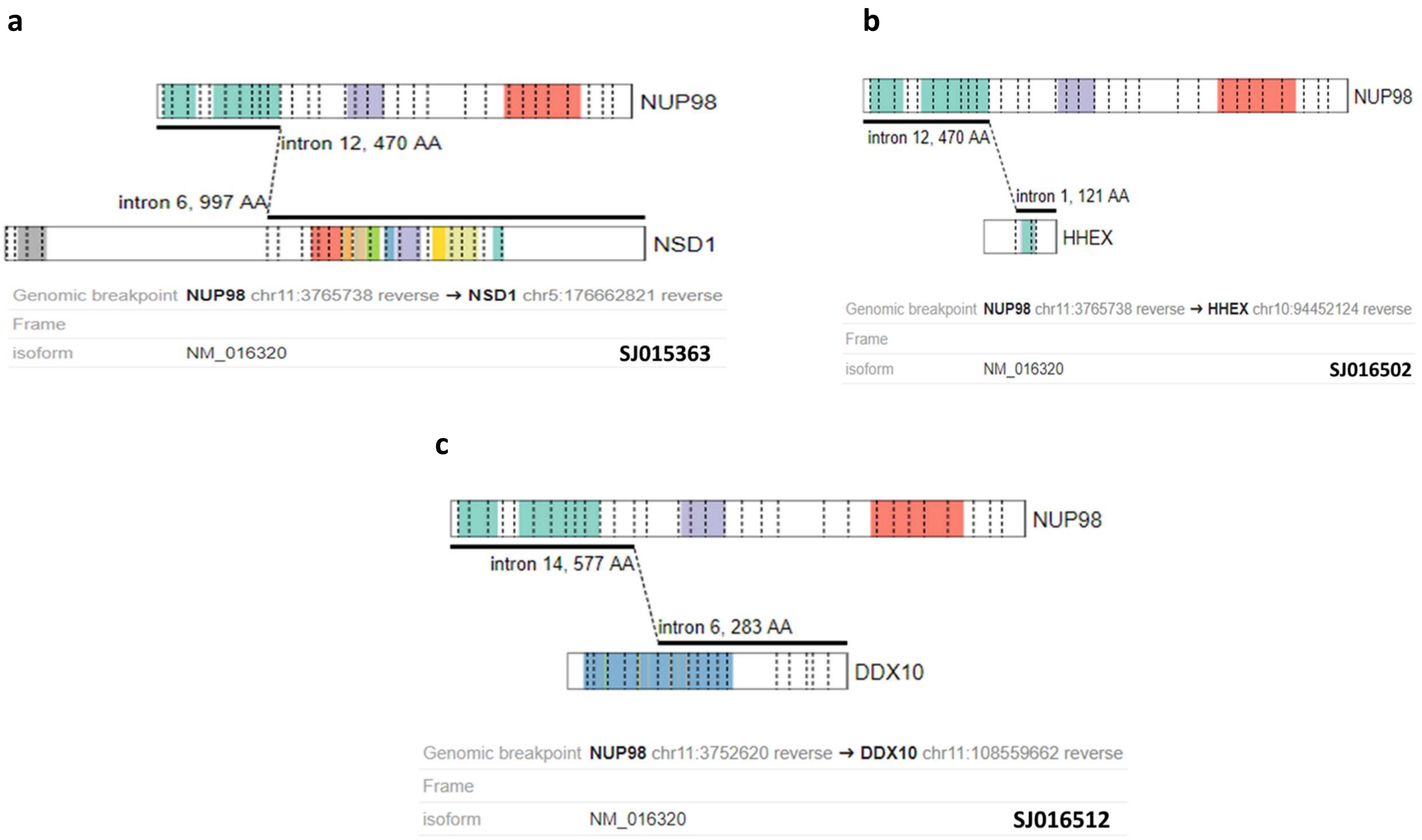

a
NUP98
intron 12, 470 AA
intron 6, 997 AA
NSD1
Genomic breakpoint NUP98 chr11:3765738 reverse → NSD1 chr5:176662821 reverse
Frame
isoform NM_016320
SJ015363
b
NUP98
intron 12, 470 AA
intron 1, 121 AA
HHEX
Genomic breakpoint NUP98 chr11:3765738 reverse → HHEX chr10:94452124 reverse
Frame
isoform NM_016320
SJ016502
c
NUP98
intron 14, 577 AA
intron 6, 283 AA
DDX10
Genomic breakpoint NUP98 chr11:3752620 reverse → DDX10 chr11:108559662 reverse
Frame
isoform NM_016320
SJ016512

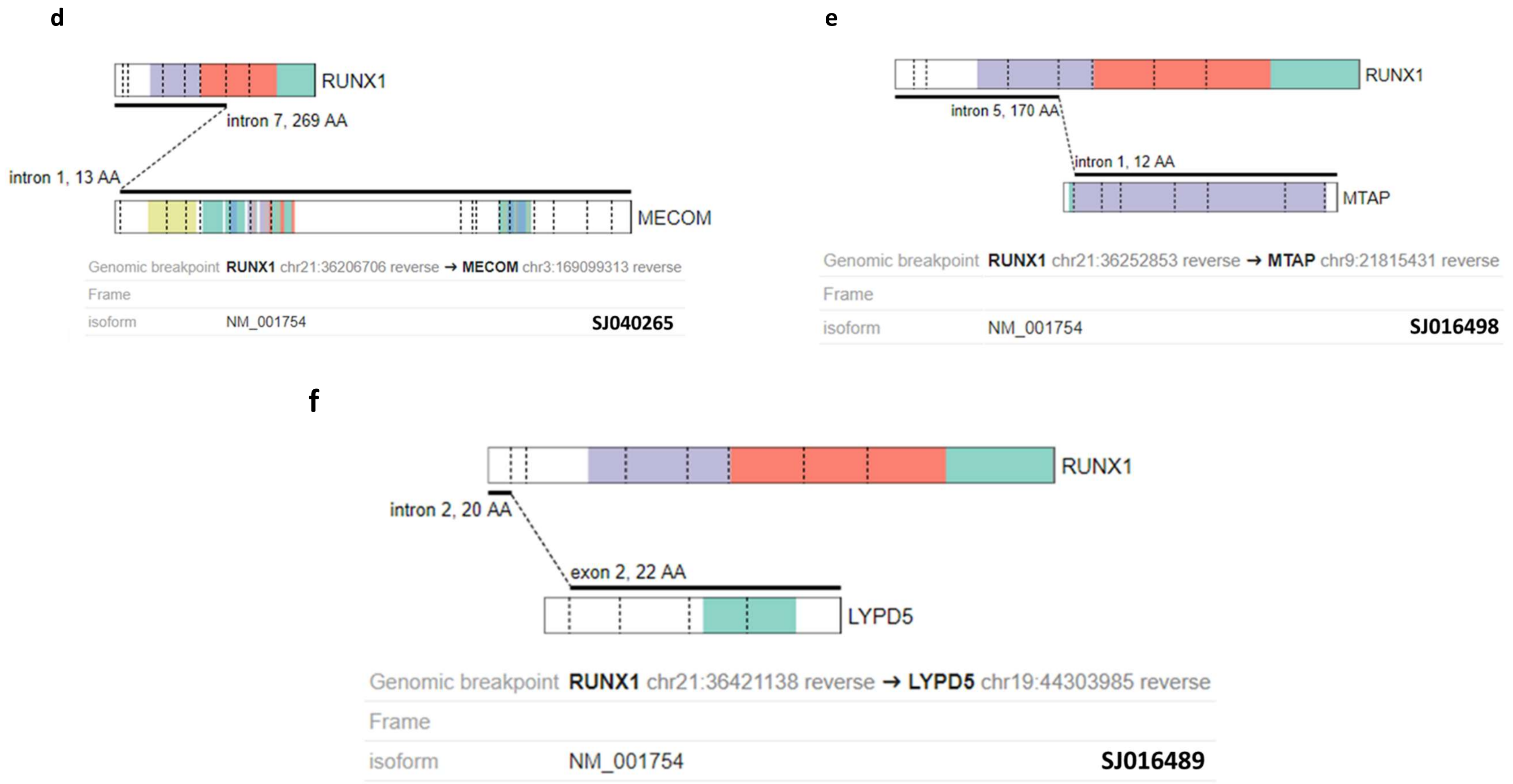
d
RUNX1
intron 7, 269 AA
intron 1, 13 AA
MECOM
Genomic breakpoint RUNX1 chr21:36206706 reverse → MECOM chr3:169099313 reverse
Frame
isoform NM_001754
SJ040265
e
RUNX1
intron 5, 170 AA
intron 1, 12 AA
MTAP
Genomic breakpoint RUNX1 chr21:36252853 reverse → MTAP chr9:21815431 reverse
Frame
isoform NM_001754
SJ016498
f
RUNX1
intron 2, 20 AA
exon 2, 22 AA
LYPD5
Genomic breakpoint RUNX1 chr21:36421138 reverse → LYPD5 chr19:44303985 reverse
Frame
isoform NM_001754
SJ016489

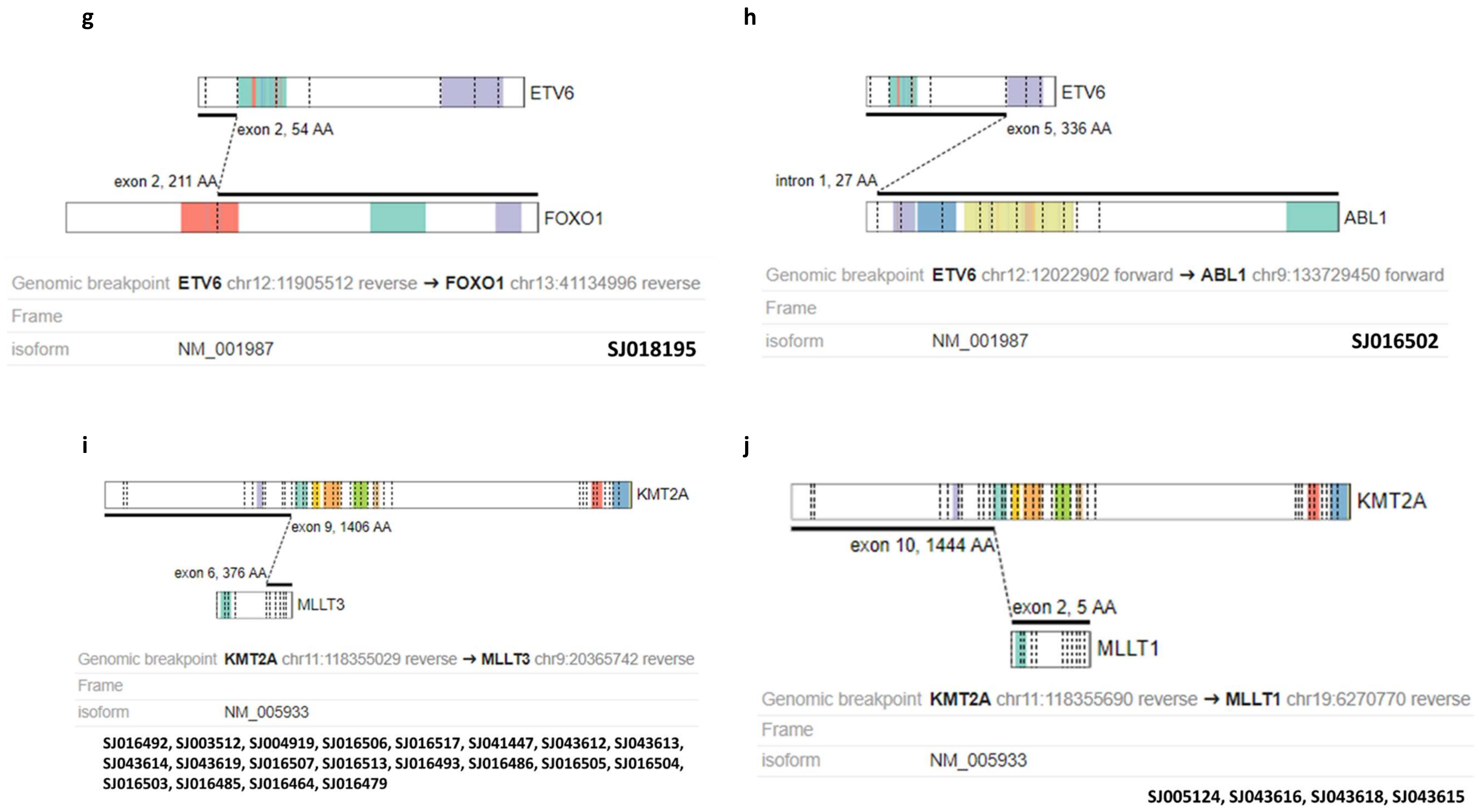
g
ETV6
exon 2, 54 AA
exon 2, 211 AA
FOXO1
Genomic breakpoint ETV6 chr12:11905512 reverse → FOXO1 chr13:41134996 reverse
Frame
isoform NM_001987
SJ018195
h
ETV6
exon 5, 336 AA
intron 1, 27 AA
ABL1
Genomic breakpoint ETV6 chr12:12022902 forward → ABL1 chr9:133729450 forward
Frame
isoform NM_001987
SJ016502
i
KMT2A
exon 9, 1406 AA
exon 6, 376 AA
MLLT3
Genomic breakpoint KMT2A chr11:118355029 reverse → MLLT3 chr9:20365742 reverse
Frame
isoform NM_005933
SJ016492, SJ003512, SJ004919, SJ016506, SJ016517, SJ041447, SJ043612, SJ043613, SJ043614, SJ043619, SJ016507, SJ016513, SJ016493, SJ016486, SJ016505, SJ016504, SJ016503, SJ016485, SJ016464, SJ016479
j
KMT2A
exon 10, 1444 AA
exon 2, 5 AA
MLLT1
Genomic breakpoint KMT2A chr11:118355690 reverse → MLLT1 chr19:6270770 reverse
Frame
isoform NM_005933
SJ005124, SJ043616, SJ043618, SJ043615

k

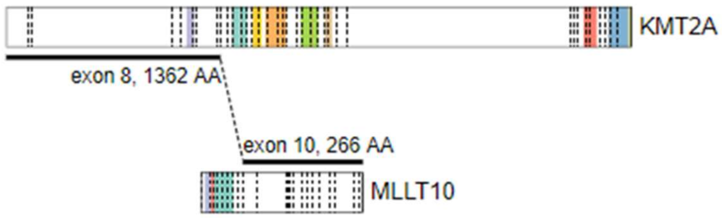

Genomic breakpoint	**KMT2A** chr11:118353210 reverse → **MLLT10** chr10:21959378 reverse
Frame	
isoform	NM_005933

SJ005142, SJ016462, SJ016500

l

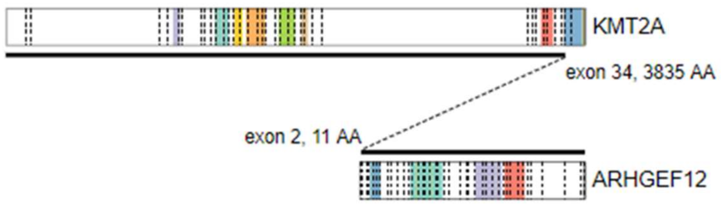

Genomic breakpoint	**KMT2A** chr11:118391600 reverse → **ARHGEF12** chr10:120276827 reverse
Frame	
isoform	NM_005933

SJ016489

m

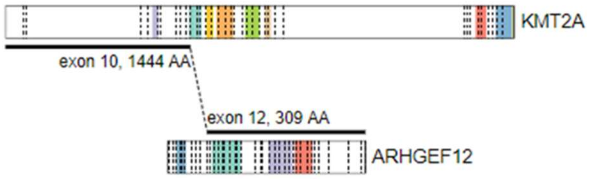

Genomic breakpoint	**KMT2A** chr11:118355690 reverse → **ARHGEF12** chr11:120308017 reverse
Frame	
isoform	NM_005933

SJ015847, SJ021960

n

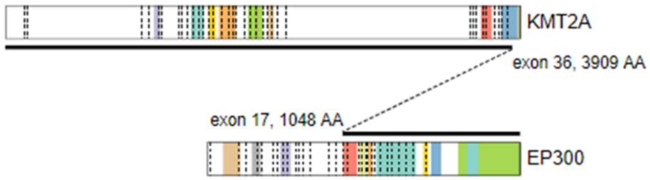

Genomic breakpoint	**KMT2A** chr11:118392702 reverse → **EP300** chr22:41550999 reverse
Frame	
isoform	NM_005933

SJ016491

o **p**

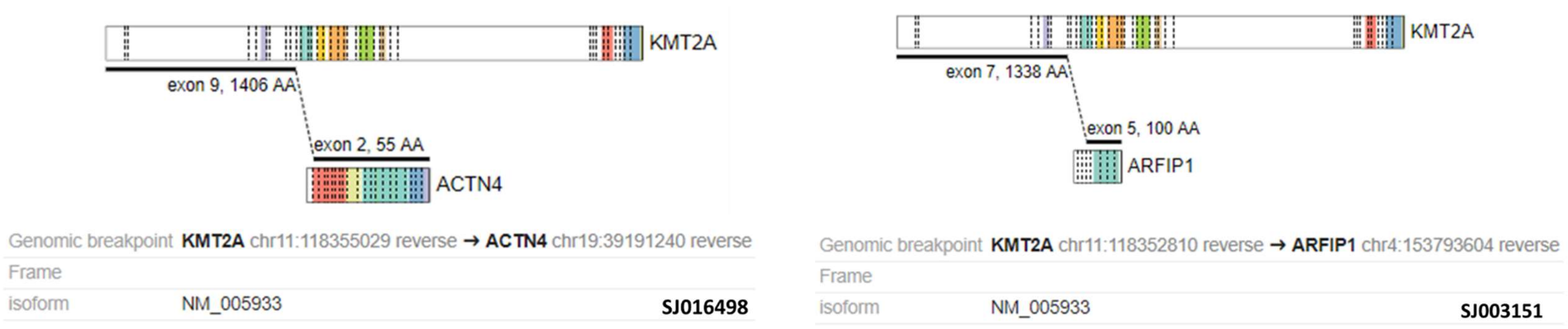

Supplementary Figure 10. Protein domain architecture of the in-frame fusions involving *NUP98* (a-c), *RUNX1* (d-f), and *ETV6* (g-h) present in pediatric tMN. i-p) Secondary protein structure of the *KMT2A*r found in the pediatric tMN cohort.

a

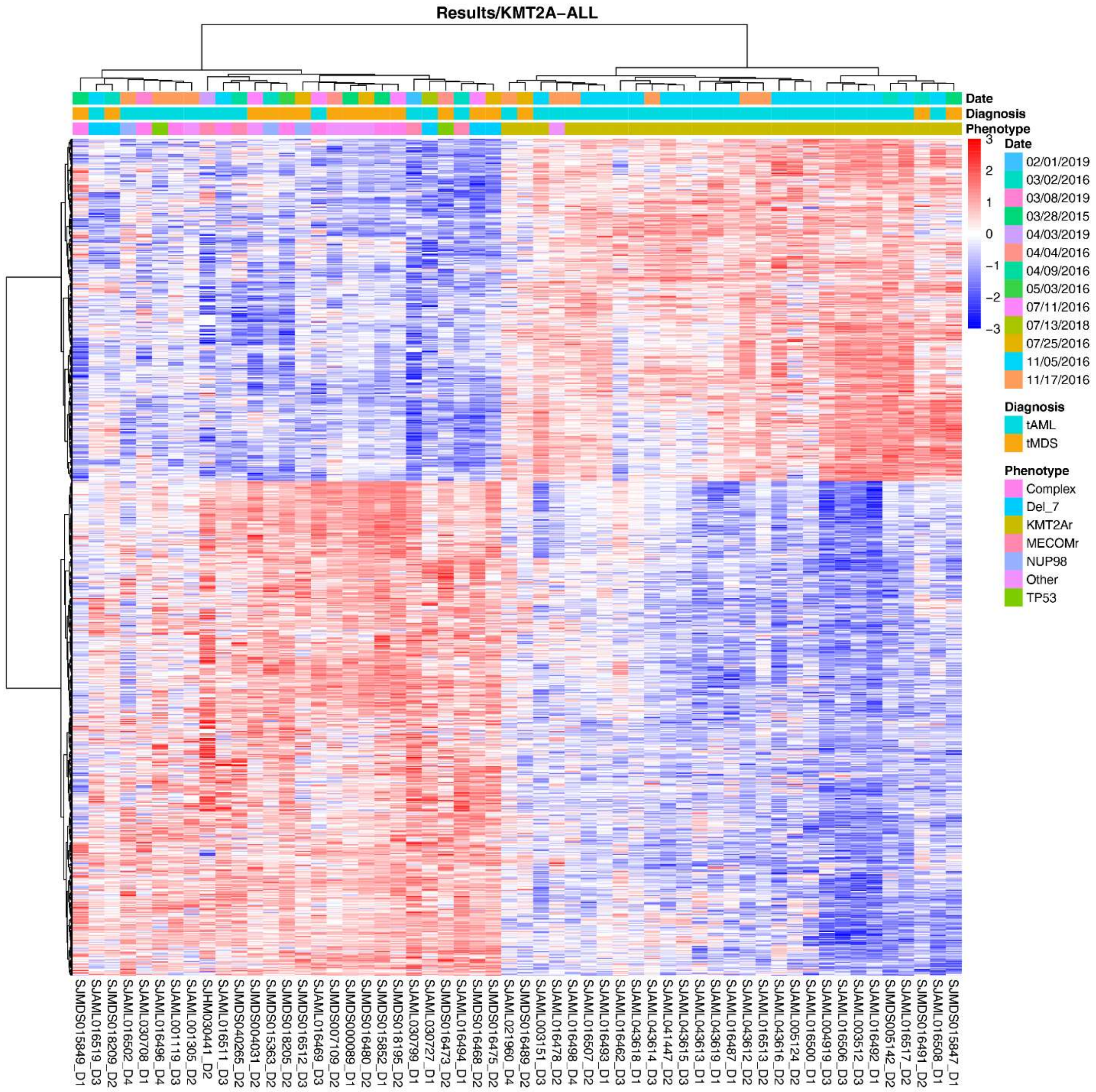

Results/KMT2A−ALL
Date
Diagnosis
Phenotype
3
2
1
0
−1
−2
−3
Date
02/01/2019
03/02/2016
03/08/2019
03/28/2015
04/03/2019
04/04/2016
04/09/2016
05/03/2016
07/11/2016
07/13/2018
07/25/2016
11/05/2016
11/17/2016
Diagnosis
tAML
tMDS
Phenotype
Complex
Del_7
KMT2Ar
MECOMr
NUP98
Other
TP53
SJMDS015849_D1
SJAML016519_D3
SJMDS018209_D2
SJAML016502_D4
SJAML030708_D1
SJAML016496_D4
SJAML001119_D3
SJAML001305_D2
SJHM030441_D2
SJAML016511_D3
SJMDS040265_D2
SJMDS004031_D2
SJMDS015363_D2
SJMDS018205_D2
SJMDS016512_D3
SJAML016469_D3
SJMDS007109_D2
SJMDS000089_D1
SJMDS016480_D2
SJMDS015852_D1
SJMDS018195_D2
SJAML030799_D1
SJAML030727_D1
SJMDS016473_D2
SJAML016494_D1
SJMDS016468_D2
SJMDS016475_D2
SJAML021960_D4
SJMDS016489_D2
SJAML003151_D3
SJAML016478_D2
SJAML016498_D4
SJAML016507_D2
SJAML016493_D1
SJAML016462_D3
SJAML043618_D1
SJAML043614_D3
SJAML041447_D2
SJAML043615_D3
SJAML043613_D1
SJAML043619_D1
SJAML016487_D1
SJAML043612_D2
SJAML016513_D2
SJAML043616_D2
SJAML005124_D2
SJAML016500_D1
SJAML004919_D3
SJAML016506_D3
SJAML003512_D3
SJAML016492_D1
SJMDS005142_D2
SJAML016517_D2
SJMDS016491_D2
SJAML016508_D1
SJMDS015847_D1

b

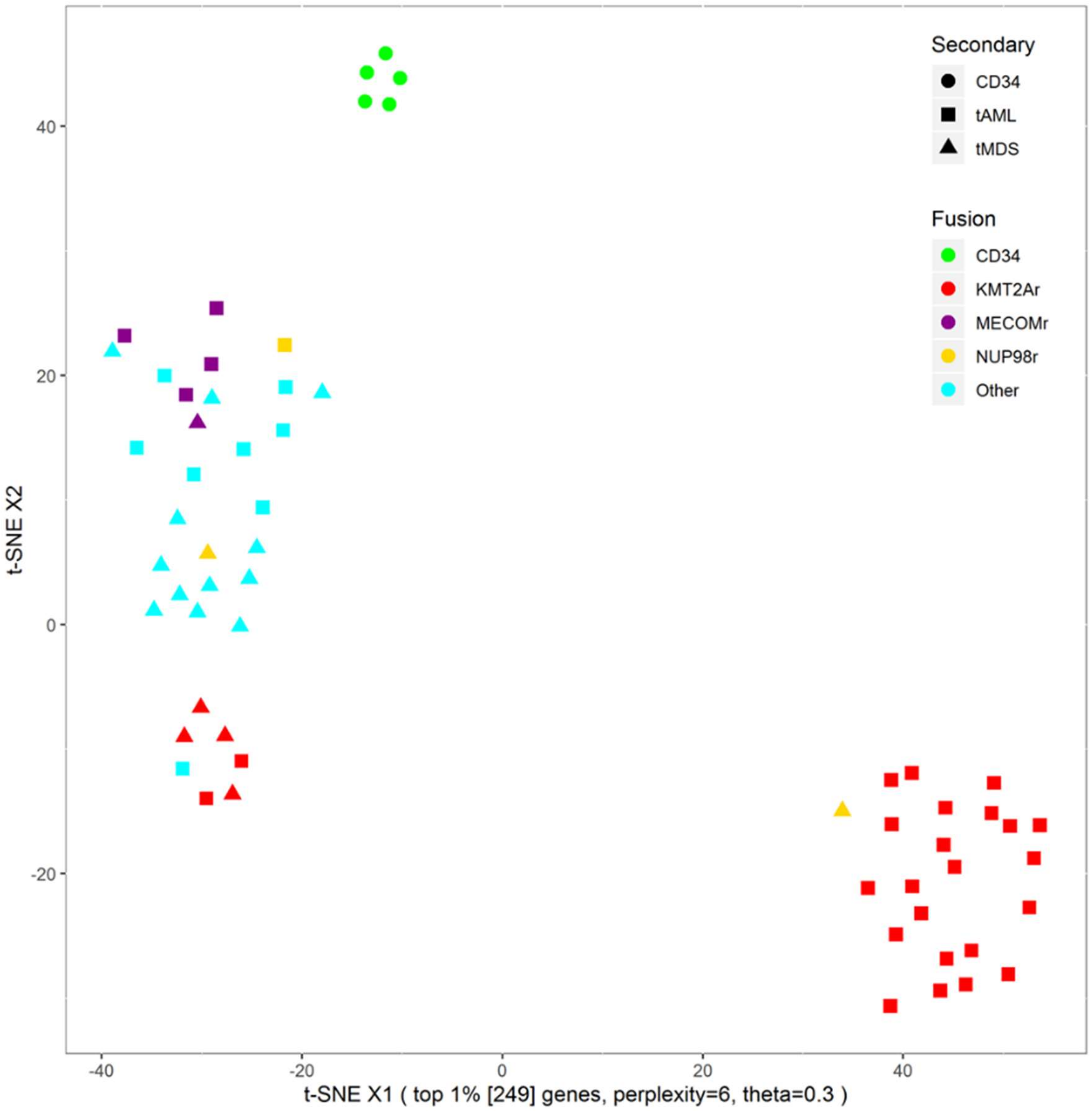

Supplementary Figure 11. a) Heat map showing differentially expressed genes in those cases with and without *KMT2A*r. b) tSNE plot using RNA expression data showing the majority of *KMT2A*r cases cluster together and all except for a single case with a *NUP98*r are tAML. Interestingly, more than half of the smaller cluster of cases with *KMT2Ar* are tMDS cases. Five different cases of cord blood CD34+ cells (green circles) were used as a control and they cluster separately than both tMDS and tAML cases.

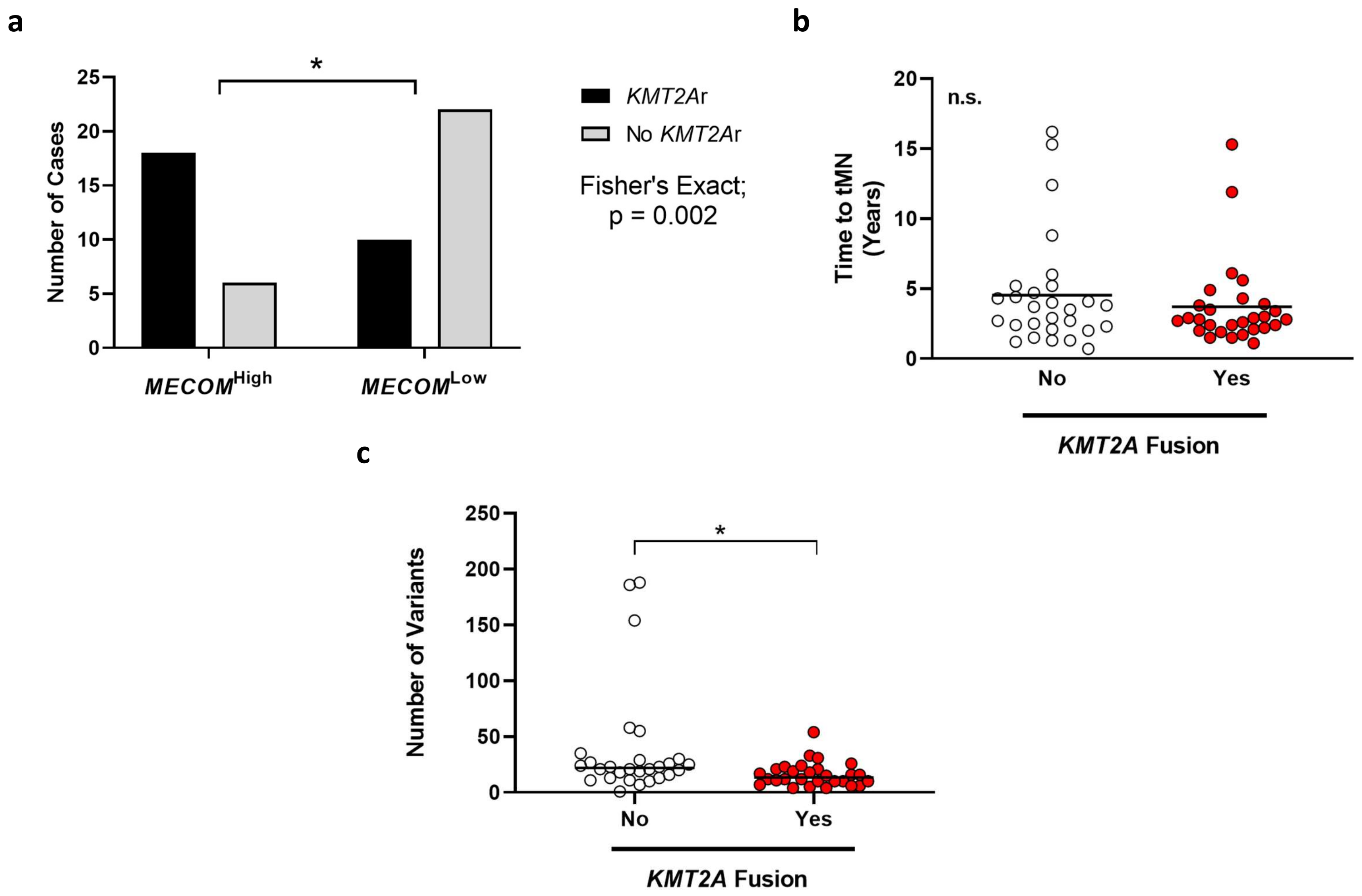

Supplementary Figure 12. a) Contingency bar graph showing a statistically significant (p=0.02, Fisher's exact test, two tailed) enrichment of *KMT2A*r within the *MECOM*^{High} group; n=56 biologically independent samples. b) Grouped plot showing no significant difference (p=0.4319) in time to tMN when considering the presence of a *KMT2Ar* in those with RNA Seq data (n=56). c) Grouped plot showing a significant difference in the total somatic mutation burden (no: 39mut/patient vs. yes: 16 mut/patient; p<0.01), and this significant difference remains when the 3 hypermutated cases are excluded (no: .22mut/patient vs. yes: 16mut/patient; p=0.03). Black bars in panels b and c indicate median value. The Wilcoxon-Mann-Whitney non-parametric test, two-tailed test was used for comparisons in panels b and c.

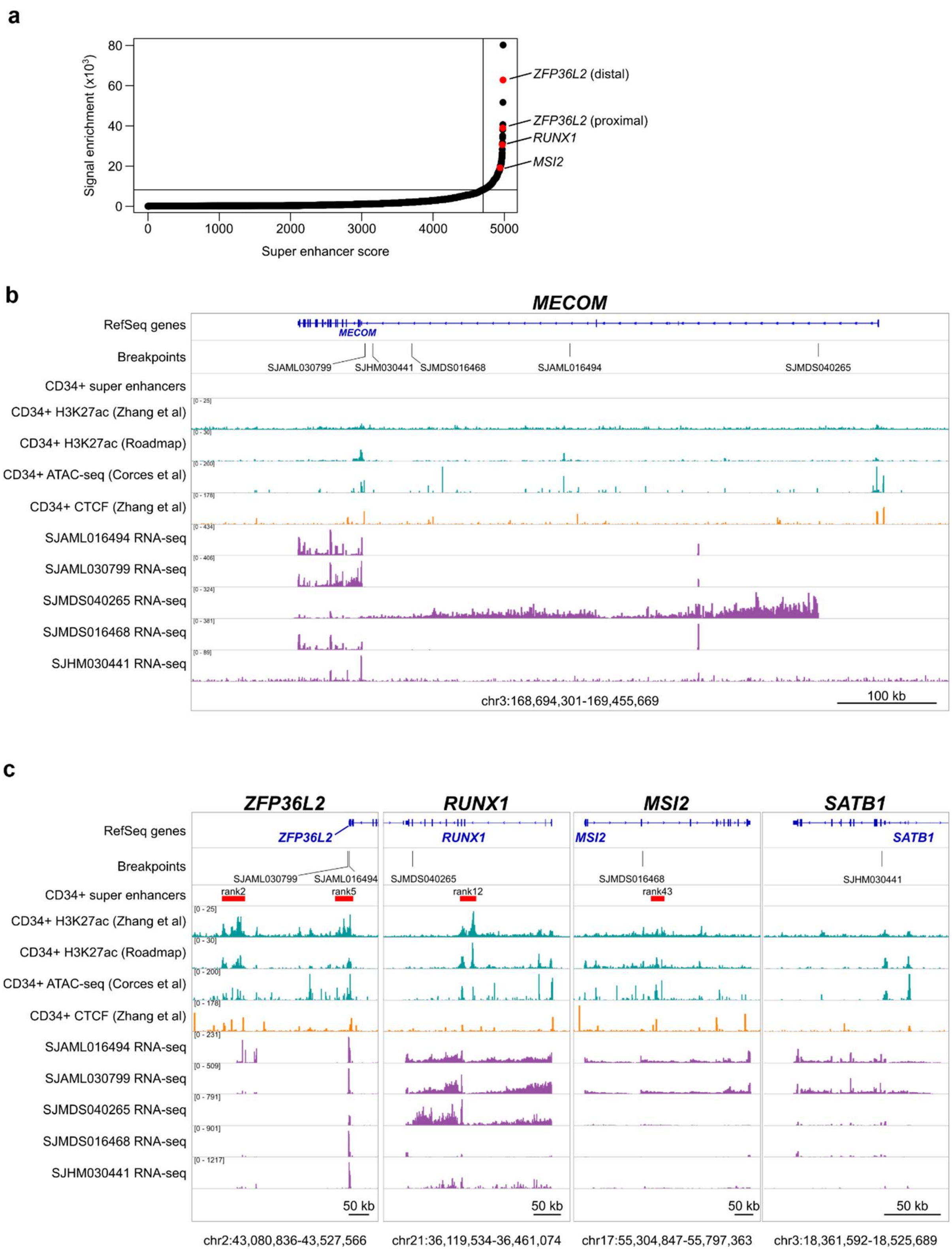

a
Signal enrichment (x10^3)
80
60
40
20
0
0
1000
2000
3000
4000
5000
Super enhancer score
ZFP36L2 (distal)
ZFP36L2 (proximal)
RUNX1
MSI2
b
MECOM
RefSeq genes
MECOM
Breakpoints
SJAML030799
SJHM030441
SJMDS016468
SJAML016494
SJMDS040265
CD34+ super enhancers
CD34+ H3K27ac (Zhang et al)
CD34+ H3K27ac (Roadmap)
CD34+ ATAC-seq (Corces et al)
CD34+ CTCF (Zhang et al)
SJAML016494 RNA-seq
SJAML030799 RNA-seq
SJMDS040265 RNA-seq
SJMDS016468 RNA-seq
SJHM030441 RNA-seq
chr3:168,694,301-169,455,669
100 kb
c
ZFP36L2
RUNX1
MSI2
SATB1
RefSeq genes
Breakpoints
SJAML030799
SJAML016494
SJMDS040265
SJMDS016468
SJHM030441
CD34+ super enhancers
rank2
rank5
rank12
rank43
CD34+ H3K27ac (Zhang et al)
CD34+ H3K27ac (Roadmap)
CD34+ ATAC-seq (Corces et al)
CD34+ CTCF (Zhang et al)
SJAML016494 RNA-seq
SJAML030799 RNA-seq
SJMDS040265 RNA-seq
SJMDS016468 RNA-seq
SJHM030441 RNA-seq
50 kb
chr2:43,080,836-43,527,566
chr21:36,119,534-36,461,074
chr17:55,304,847-55,797,363
chr3:18,361,592-18,525,689

Supplementary Figure 13. Super enhancer analysis of *MECOM* partner breakpoint regions in CD34⁺ cells. (a) Results from the Ranking Of Super Enhancers (ROSE)[6] analysis on H3K27ac ChIP-seq data from human CD34+ cells[7]. Each dot represents a stitched H3K27ac peak locus (candidate super enhancer) which is ranked according to the signal enrichment over input (y-axis). A total of 278 super enhancers were identified at a cut-off of signal enrichment of 8264 (horizontal line). All points to the right of the vertical line represent super enhancers, and those falling near *MECOM* break points are highlighted in red. (b) IGV browser snapshot depicting the *MECOM* locus. The positions of each breakpoint are shown along with H3K27ac ChIP-seq[7], ATAC-seq[8], and CTCF ChIP-seq[7] data from human $CD34^+$ cells. RNA-seq tracks are shown for each of the *MECOM*-rearranged patient samples. (C) IGV browser snapshot depicting each *MECOM* partner break point and highlighting the presence of at least one super enhancer in 3 of the 4 *MECOM* partner loci. Tracks same as in (b). All coordinates are Hg19.

a

The markers effects on diease-related death risk

res.df2	Marker	HR	95%CI	Pvalue
1	KMT2Ar.new	0.8932	[0.375;2.123]	0.7975
2	NUP98r.new	3.1899	[0.893;11.410]	0.07417
3	del.7.new	0.865	[0.385;1.942]	0.7252
4	Complex.Karyotype.new	2.1706	[1.026;4.596]	0.04261
5	TP53.Alteration.new	1.608	[0.811;3.188]	0.1741
6	Ras.MAPK.Mutation.new	1.1735	[0.573;2.401]	0.6616
7	MECOM.log	0.9867	[0.881;1.105]	0.8164

b

The markers effects on transplant-related death risk

res.df2	Marker	HR	95%CI	Pvalue
1	KMT2Ar.new	1.5699	[0.486;5.069]	0.4503
2	NUP98r.new	0	[NA;NA]	
3	del.7.new	2.6618	[0.871;8.140]	0.08576
4	Complex.Karyotype.new	0.2837	[0.0577;1.408]	0.1234
5	TP53.Alteration.new	0.4025	[0.0517;3.137]	0.3851
6	Ras.MAPK.Mutation.new	0.8106	[0.260;2.526]	0.7167
7	MECOM.log	1.1377	[0.936;1.384]	0.1943

c

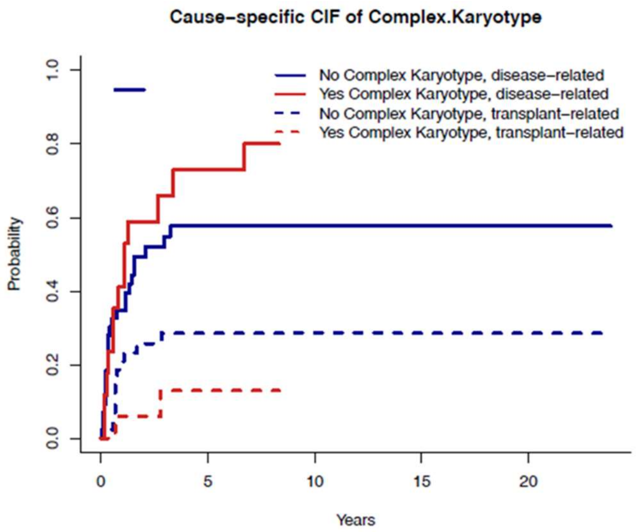

Supplementary Figure 14. Survival analysis of cause-specific death was performed via Fine-Gray modeling which is capable of handling competing risk while also considering the time-dependent binary covariate of HCT. This analysis was completed on the tumor/normal cohort (n=62). As shown in panel a, having a complex karyotype resulted in a significant impact on disease-related death, while none of the disease characteristic subgroups had any impact on transplant-related death, shown in panel b. c) Cumulative incidence plot showing the cause-specific cumulative incidence function of the complex karyotype group.

a

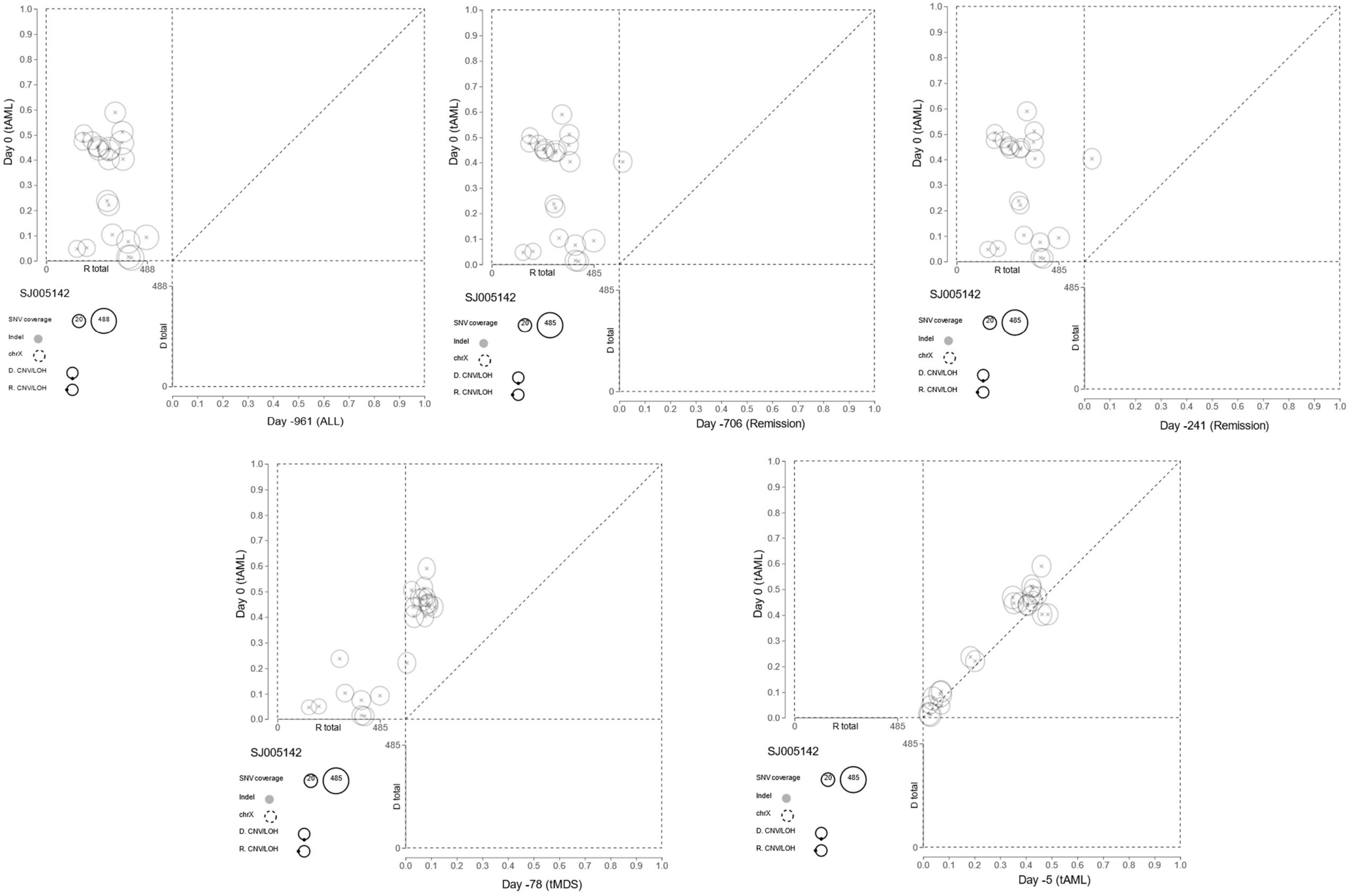
SJ005142
SNV coverage
Indel
chrX
D. CNV/LOH
R. CNV/LOH
Day 0 (tAML)
R total
D total
Day -961 (ALL)
Day -706 (Remission)
Day -241 (Remission)
Day -78 (tMDS)
Day -5 (tAML)

b

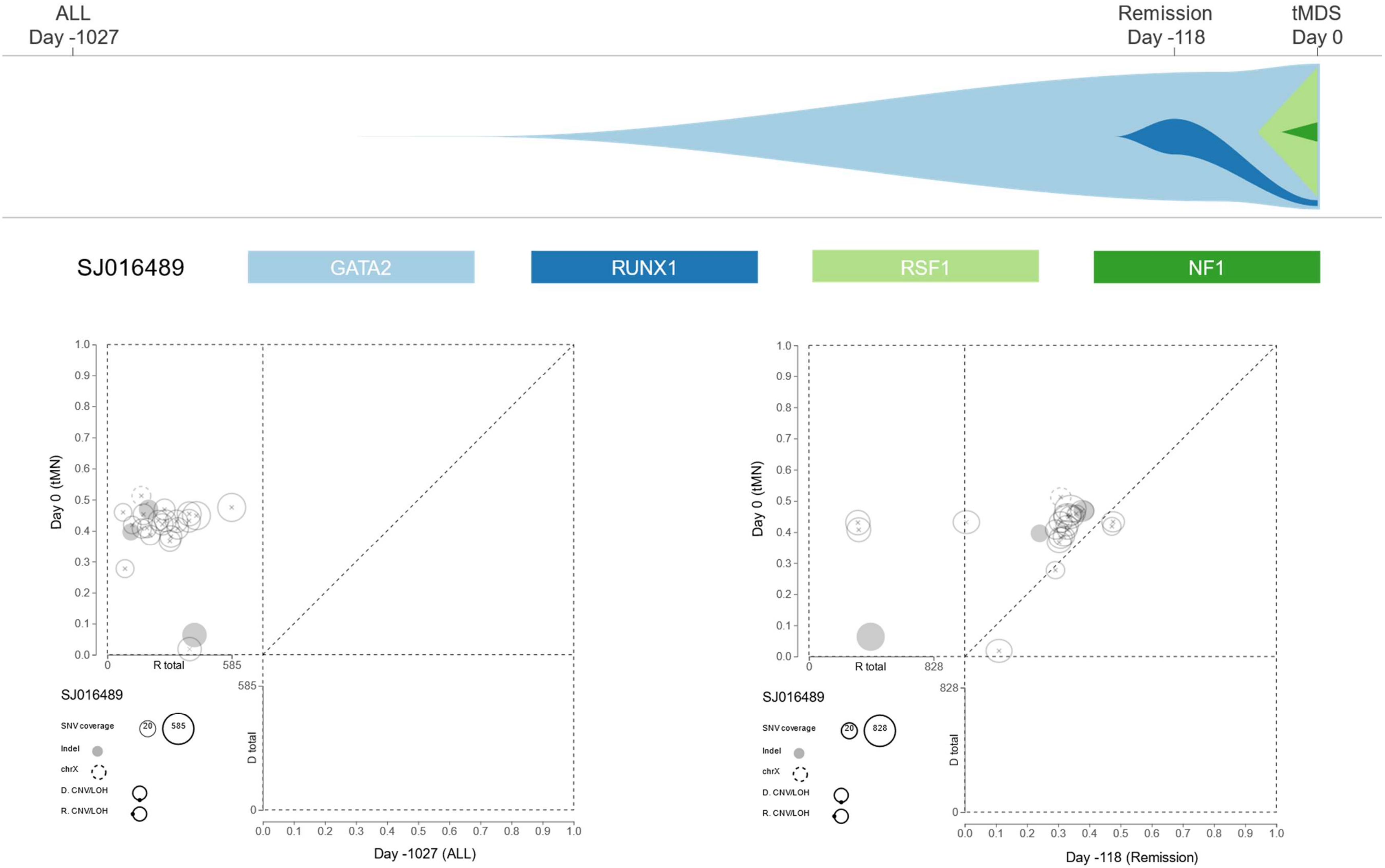

ALL
Day -1027
Remission
Day -118
tMDS
Day 0
SJ016489
GATA2
RUNX1
RSF1
NF1
Day 0 (tMN)
R total
D total
SNV coverage
Indel
chrX
D. CNV/LOH
R. CNV/LOH
Day -1027 (ALL)
Day -118 (Remission)

c

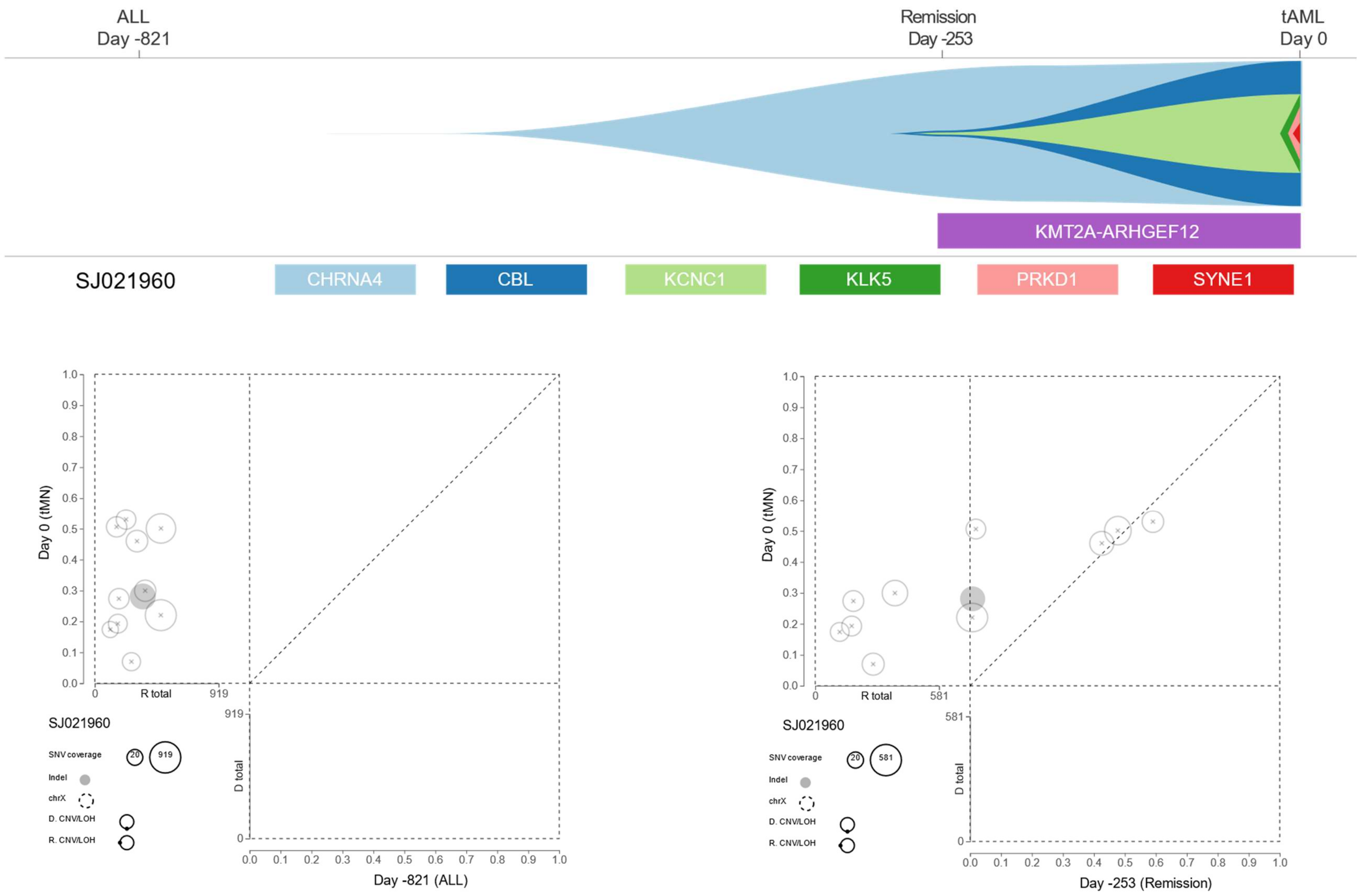

ALL
Day -821
Remission
Day -253
tAML
Day 0
KMT2A-ARHGEF12
SJ021960
CHRNA4
CBL
KCNC1
KLK5
PRKD1
SYNE1
Day 0 (tMN)
R total
919
D total
Day -821 (ALL)
SJ021960
SNV coverage
Indel
chrX
D. CNV/LOH
R. CNV/LOH
Day 0 (tMN)
R total
581
D total
Day -253 (Remission)
SJ021960
SNV coverage
Indel
chrX
D. CNV/LOH
R. CNV/LOH

d

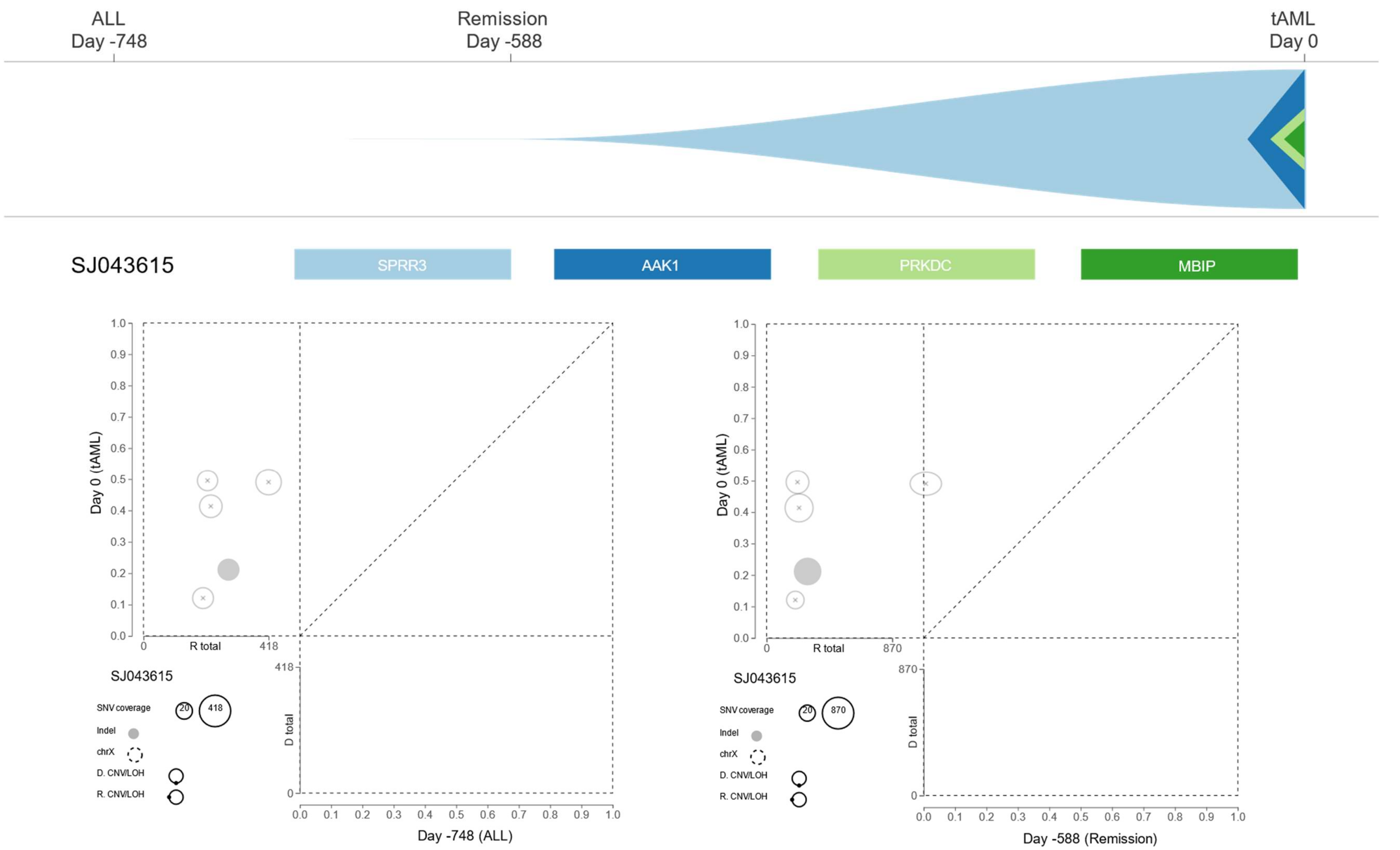
ALL
Day -748
Remission
Day -588
tAML
Day 0
SJ043615
SPRR3
AAK1
PRKDC
MBIP
Day 0 (tAML)
R total
D total
SJ043615
SNV coverage
Indel
chrX
D. CNV/LOH
R. CNV/LOH
Day -748 (ALL)
Day -588 (Remission)

e

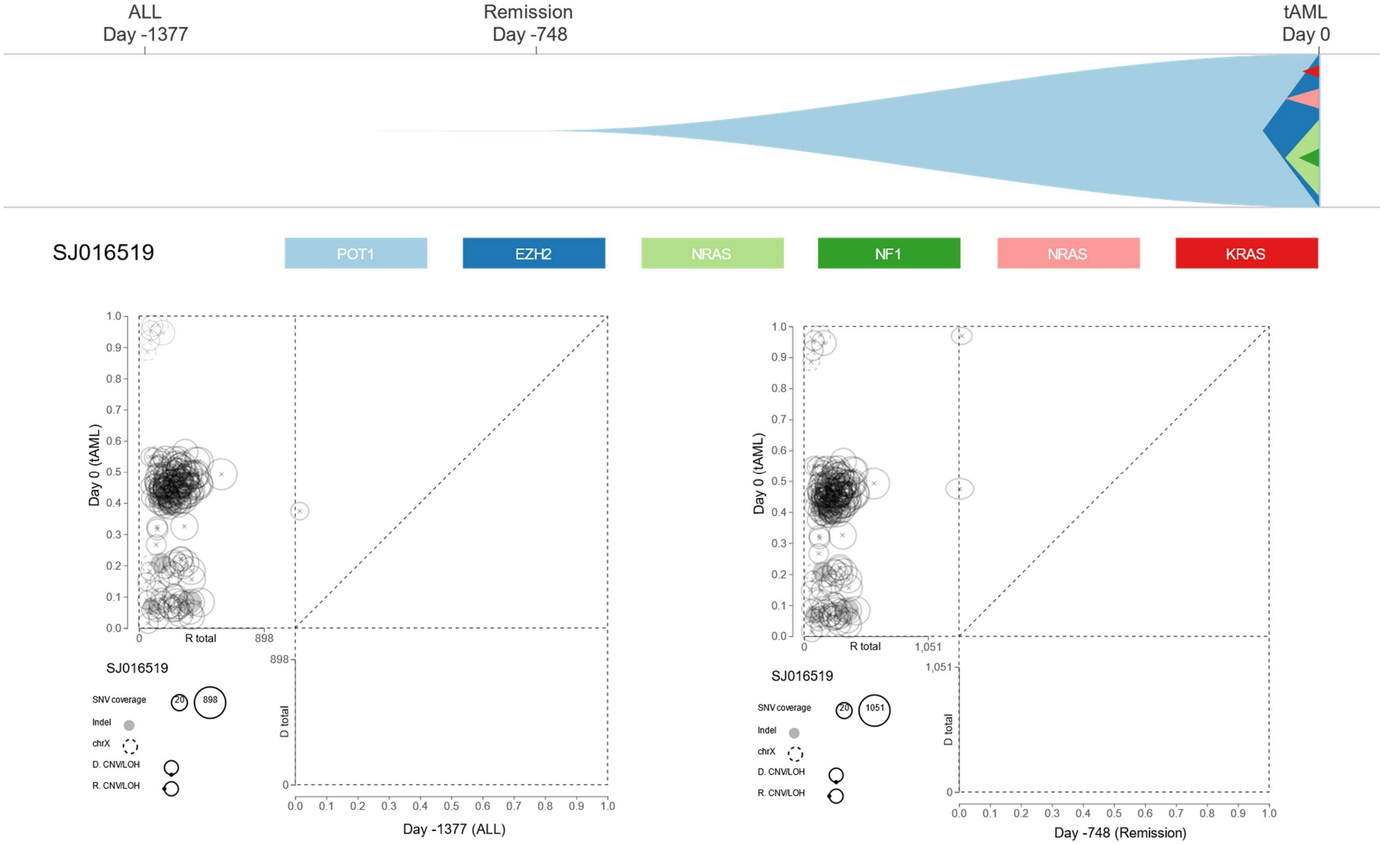
ALL
Day -1377
Remission
Day -748
tAML
Day 0
SJ016519
POT1
EZH2
NRAS
NF1
NRAS
KRAS
Day 0 (tAML)
R total
898
D total
SJ016519
SNV coverage
20
898
Indel
chrX
D. CNV/LOH
R. CNV/LOH
Day -1377 (ALL)
Day 0 (tAML)
R total
1,051
D total
SJ016519
SNV coverage
20
1051
Indel
chrX
D. CNV/LOH
R. CNV/LOH
Day -748 (Remission)

f

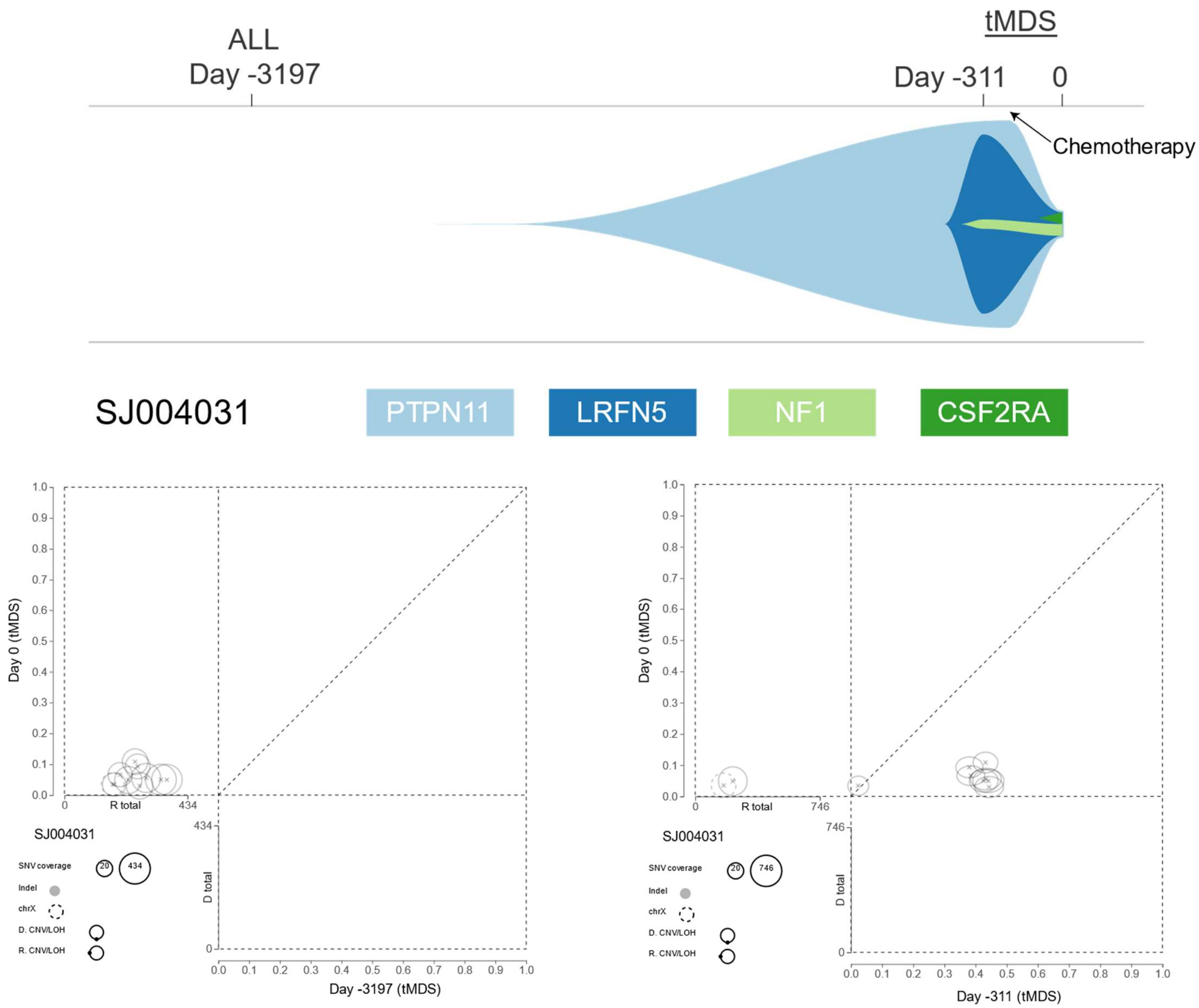

ALL
Day -3197
tMDS
Day -311
0
Chemotherapy
SJ004031
PTPN11
LRFN5
NF1
CSF2RA
Day 0 (tMDS)
R total
434
D total
SJ004031
SNV coverage
20
434
Indel
chrX
D. CNV/LOH
R. CNV/LOH
Day -3197 (tMDS)
746
Day -311 (tMDS)

g

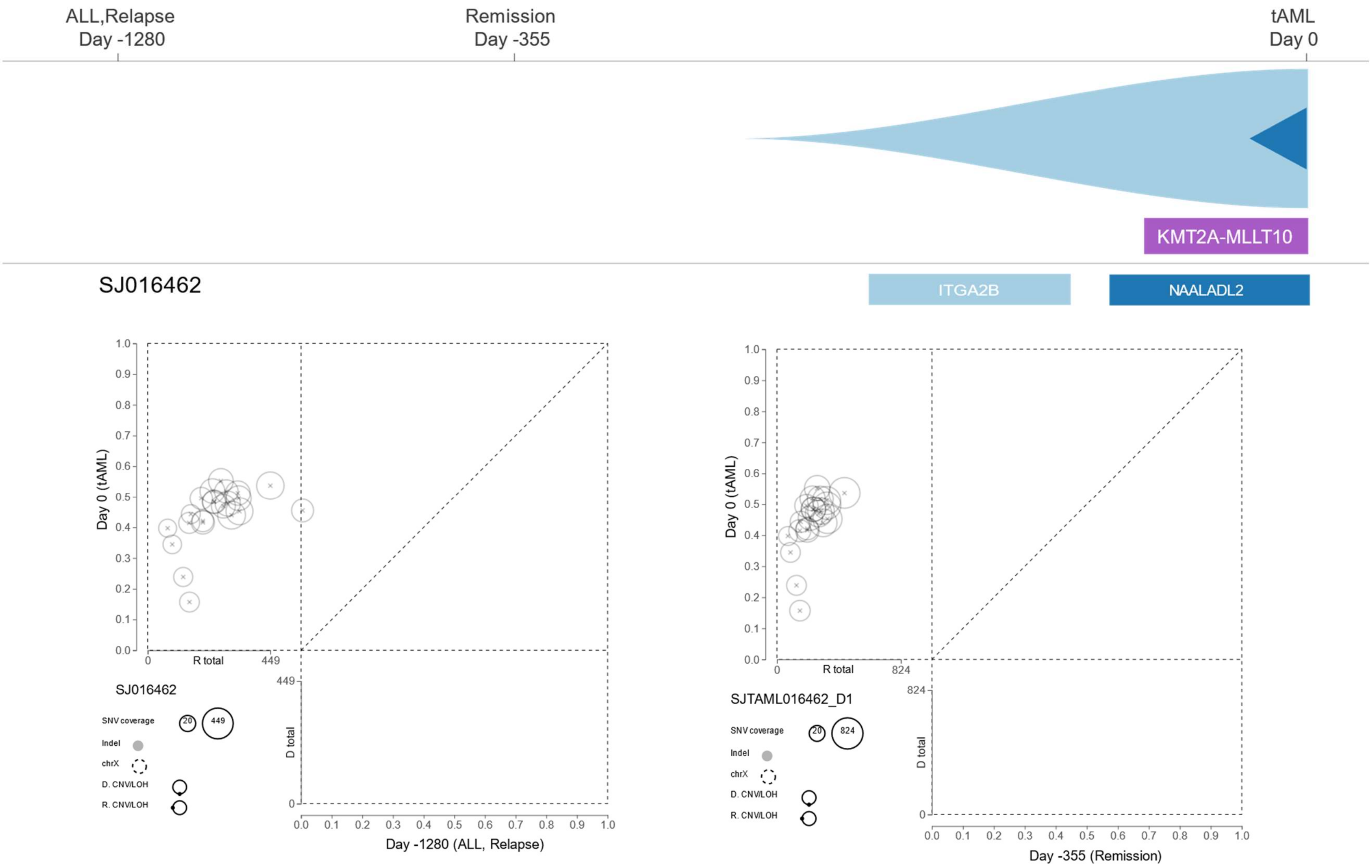

ALL,Relapse
Day -1280
Remission
Day -355
tAML
Day 0
KMT2A-MLLT10
SJ016462
ITGA2B
NAALADL2
Day 0 (tAML)
R total
449
D total
Day -1280 (ALL, Relapse)
SJ016462
SNV coverage
20
Indel
chrX
D. CNV/LOH
R. CNV/LOH
824
Day -355 (Remission)
SJTAML016462_D1

h

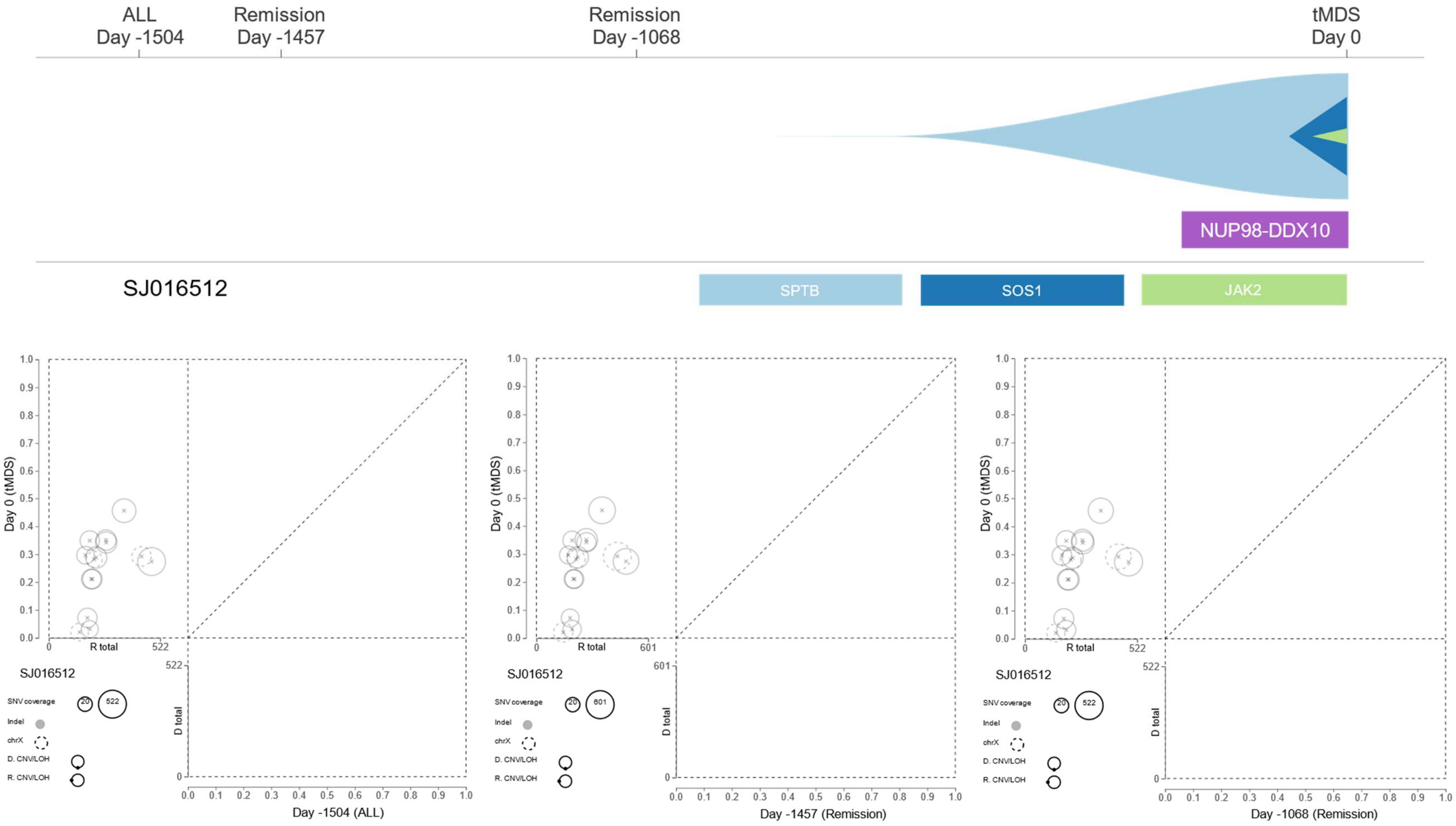
ALL
Day -1504
Remission
Day -1457
Remission
Day -1068
tMDS
Day 0
NUP98-DDX10
SJ016512
SPTB
SOS1
JAK2
Day 0 (tMDS)
R total
SJ016512
SNV coverage
Indel
chrX
D. CNV/LOH
R. CNV/LOH
D total
Day -1504 (ALL)
Day -1457 (Remission)
Day -1068 (Remission)

i

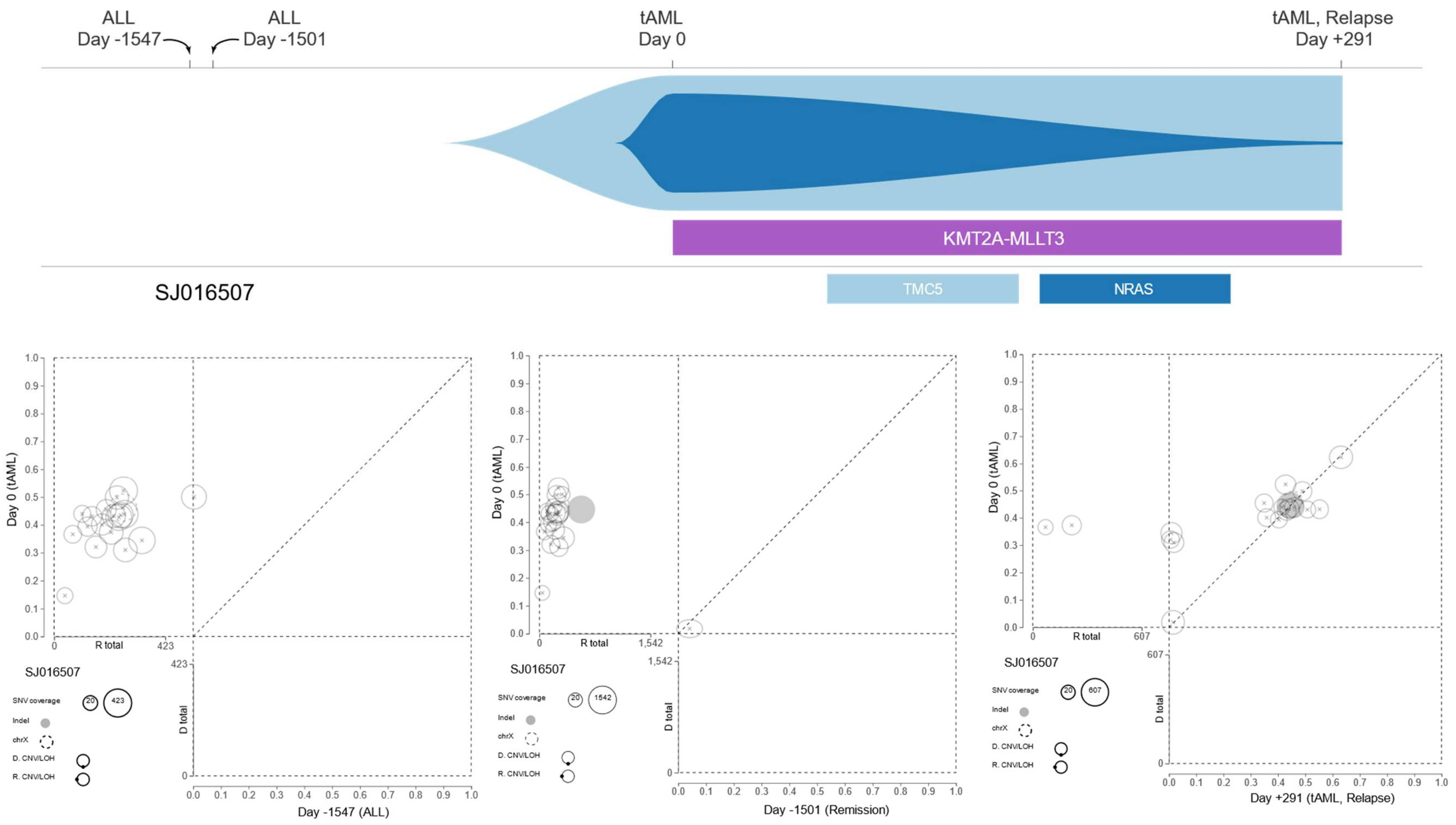

ALL Day -1547
ALL Day -1501
tAML Day 0
tAML, Relapse Day +291
KMT2A-MLLT3
SJ016507
TMC5
NRAS
Day 0 (tAML)
R total
D total
SNV coverage
Indel
chrX
D. CNV/LOH
R. CNV/LOH
Day -1547 (ALL)
Day -1501 (Remission)
Day +291 (tAML, Relapse)

j

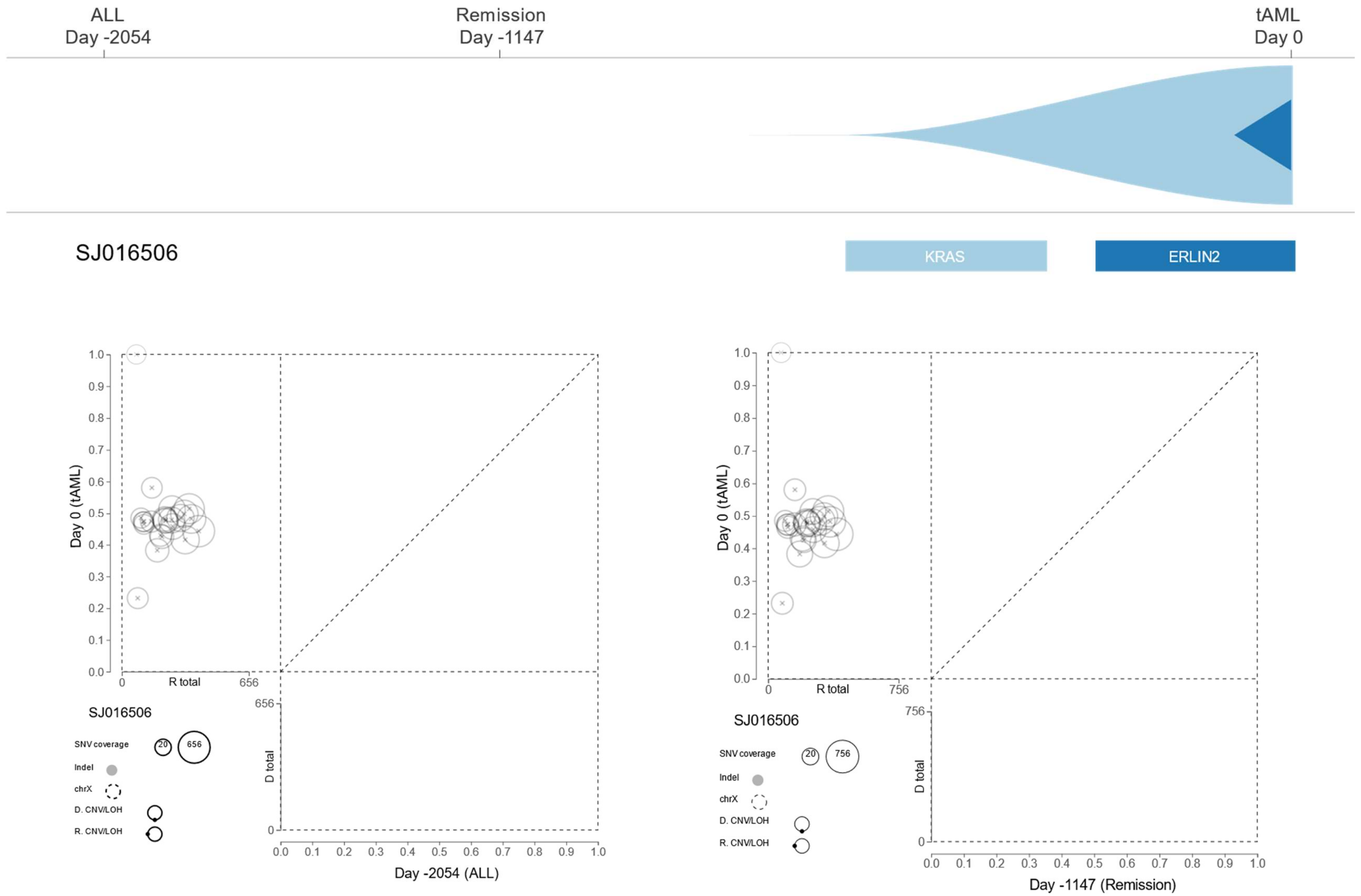

ALL
Day -2054
Remission
Day -1147
tAML
Day 0
SJ016506
KRAS
ERLIN2
Day 0 (tAML)
R total
656
D total
SJ016506
SNV coverage
20
656
Indel
chrX
D. CNV/LOH
R. CNV/LOH
Day -2054 (ALL)
Day 0 (tAML)
R total
756
D total
SJ016506
SNV coverage
20
756
Indel
chrX
D. CNV/LOH
R. CNV/LOH
Day -1147 (Remission)

k

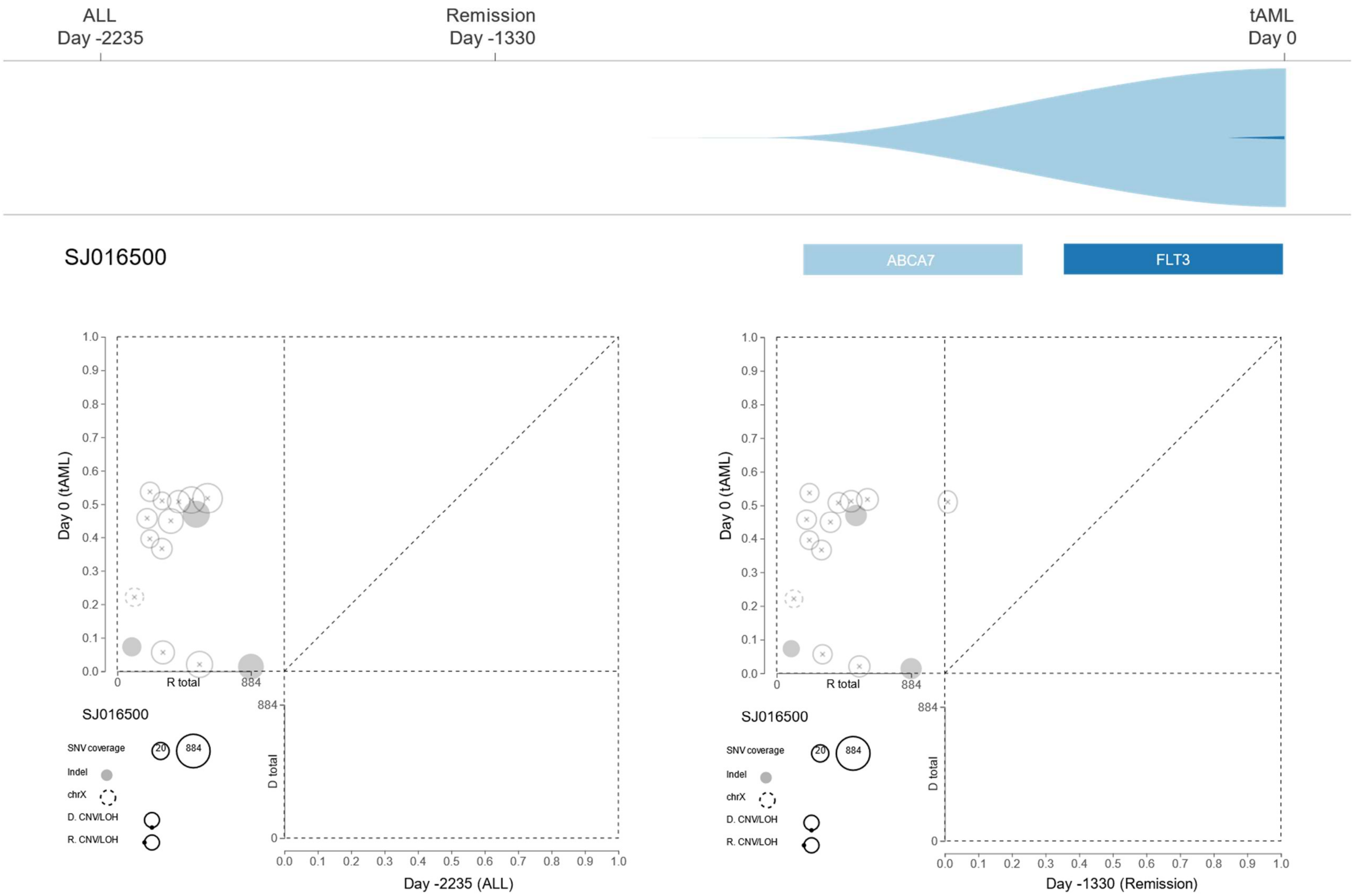

ALL
Day -2235
Remission
Day -1330
tAML
Day 0
SJ016500
ABCA7
FLT3
Day 0 (tAML)
R total
0
884
D total
SJ016500
SNV coverage
20
884
Indel
chrX
D. CNV/LOH
R. CNV/LOH
Day -2235 (ALL)
Day -1330 (Remission)
0.0
0.1
0.2
0.3
0.4
0.5
0.6
0.7
0.8
0.9
1.0

I

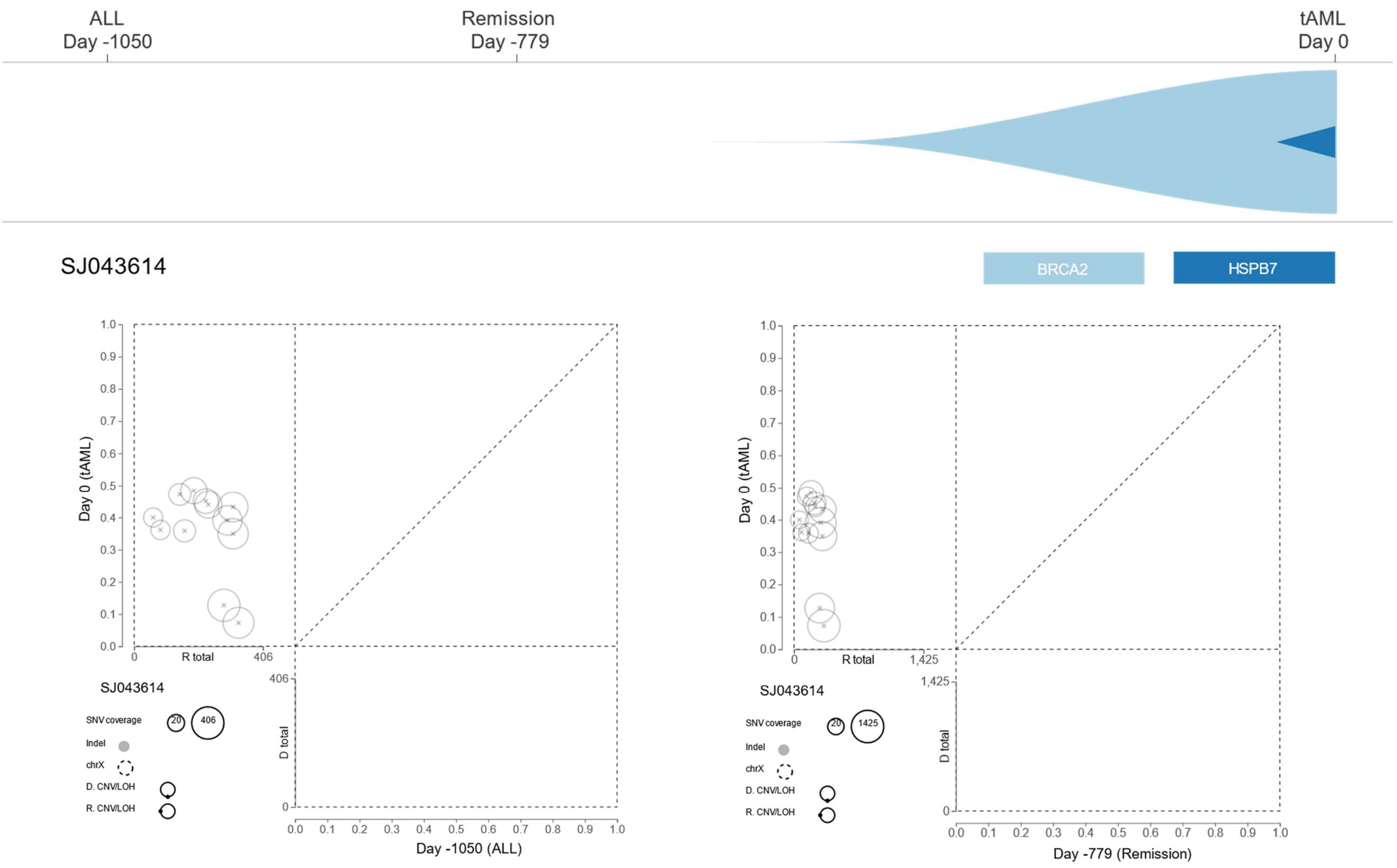

ALL
Day -1050
Remission
Day -779
tAML
Day 0
SJ043614
BRCA2
HSPB7
Day 0 (tAML)
R total
0
406
SJ043614
SNV coverage
20
406
Indel
chrX
D. CNV/LOH
R. CNV/LOH
D total
Day -1050 (ALL)
1,425
1425
Day -779 (Remission)

m

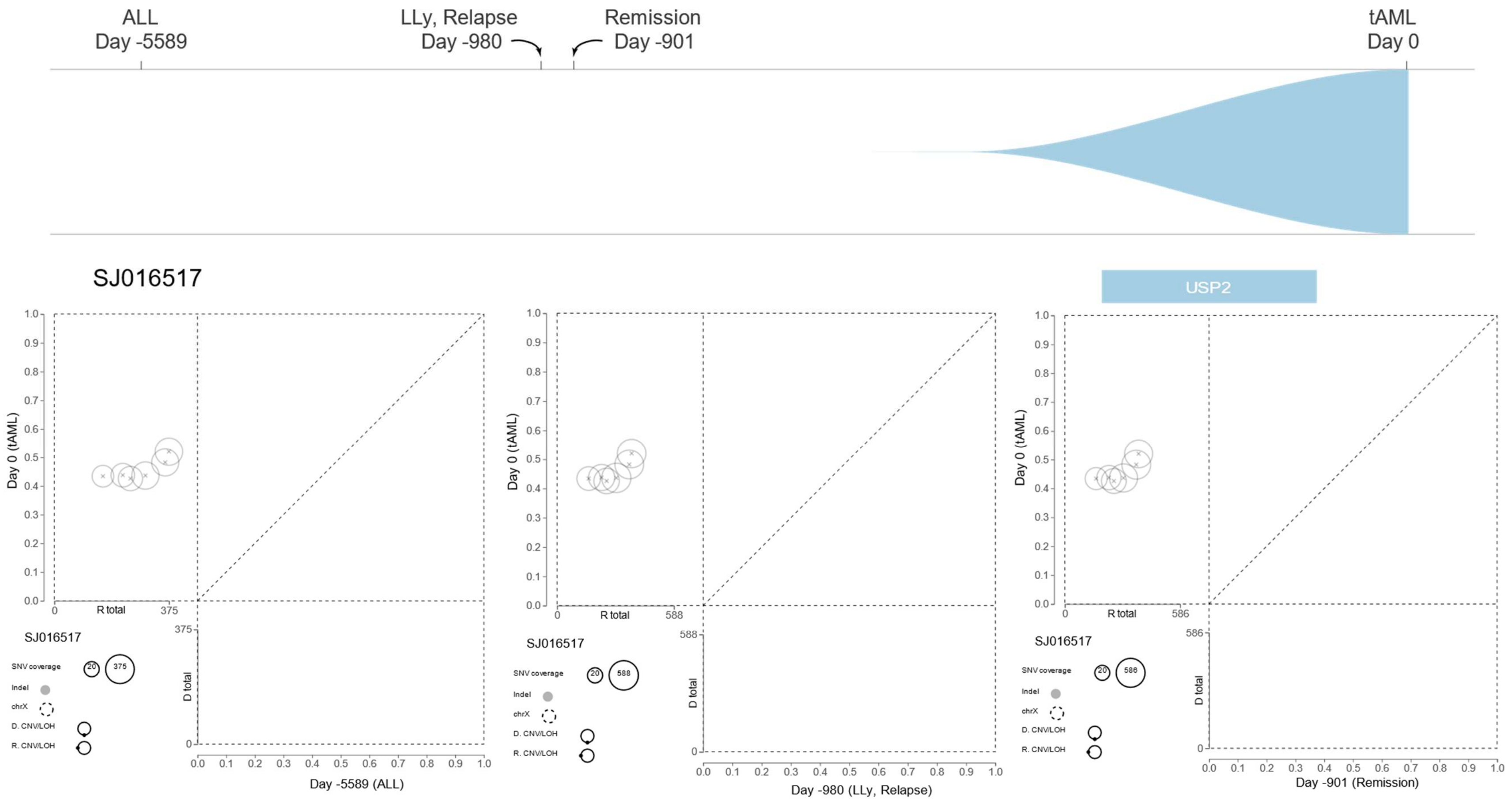

Supplementary Figure 15. a) 2-dimensional plots showing the clonal evolution present in SJ005142 (see river plot in Fig.4a). b-e) River- and 2-dimensional plots showing examples of 4 cases where tMN variants are detected 118 (b), 253 (c), 588 (d), and 748 (e) days prior to morphologic evidence of tMN. f-m) River- and 2-dimensional plots showing cases where tMN variants were neither present at initial diagnosis or at the interval timepoint available. SJ004031 (f) and SJ016507 (i) show examples of clonal changes at tMN relapse.

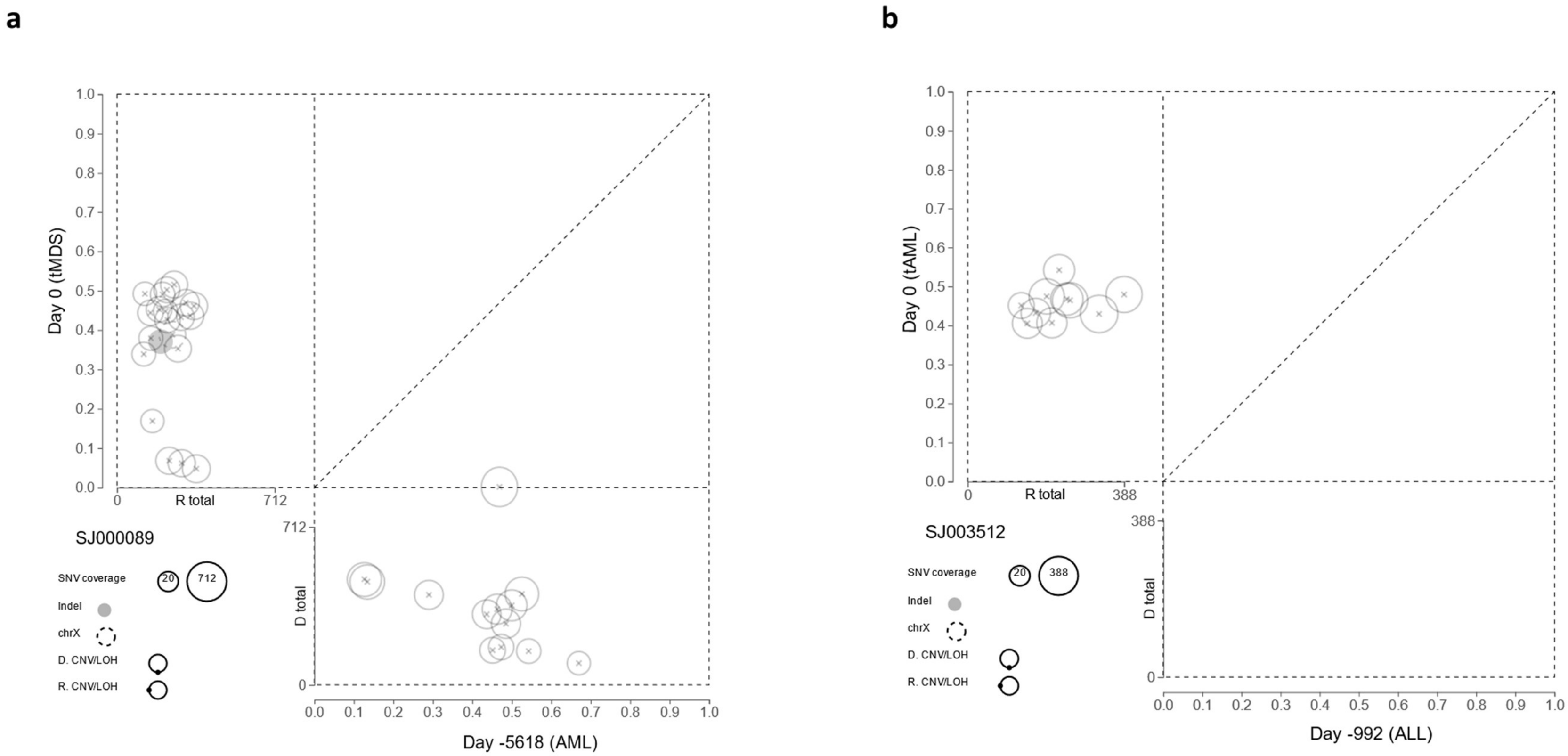
a
b
Day 0 (tMDS)
R total
D total
SJ000089
SNV coverage
20
712
Indel
chrX
D. CNV/LOH
R. CNV/LOH
Day -5618 (AML)
Day 0 (tAML)
SJ003512
388
Day -992 (ALL)

c

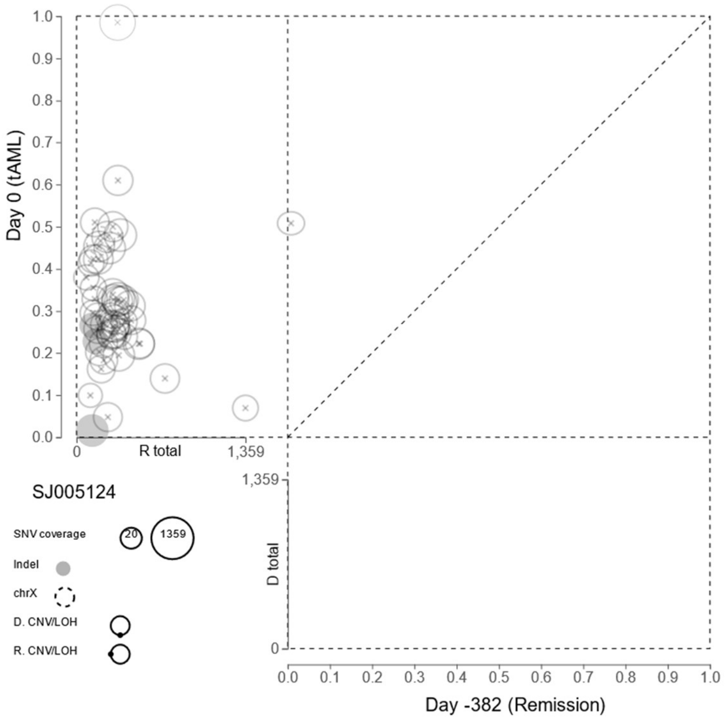
1.0
0.9
0.8
0.7
0.6
0.5
0.4
0.3
0.2
0.1
0.0
Day 0 (tAML)
0
R total
1,359
SJ005124
SNV coverage
20
1359
Indel
chrX
D. CNV/LOH
R. CNV/LOH
D total
0.0 0.1 0.2 0.3 0.4 0.5 0.6 0.7 0.8 0.9 1.0
Day -382 (Remission)

d

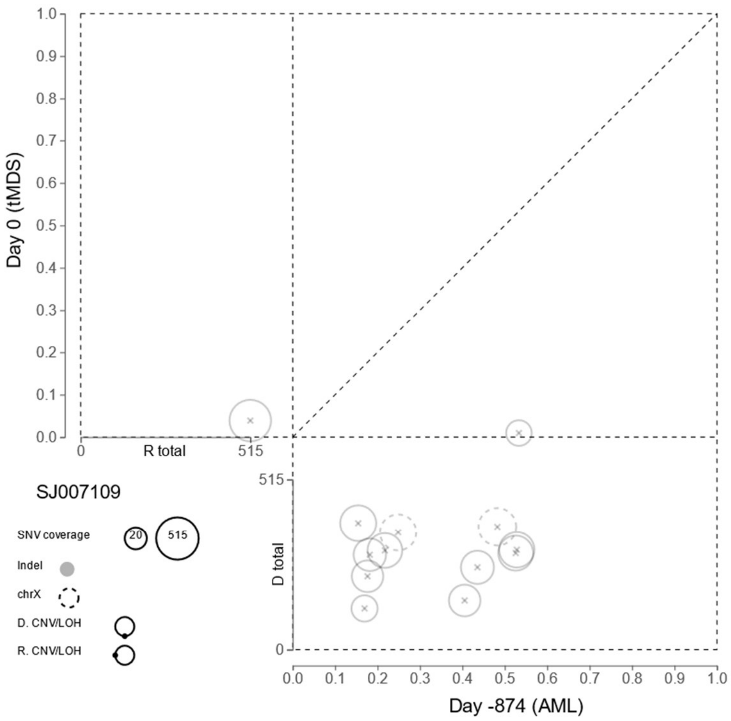
1.0
0.9
0.8
0.7
0.6
0.5
0.4
0.3
0.2
0.1
0.0
Day 0 (tMDS)
0
R total
515
SJ007109
SNV coverage
20
515
Indel
chrX
D. CNV/LOH
R. CNV/LOH
D total
0.0 0.1 0.2 0.3 0.4 0.5 0.6 0.7 0.8 0.9 1.0
Day -874 (AML)

e

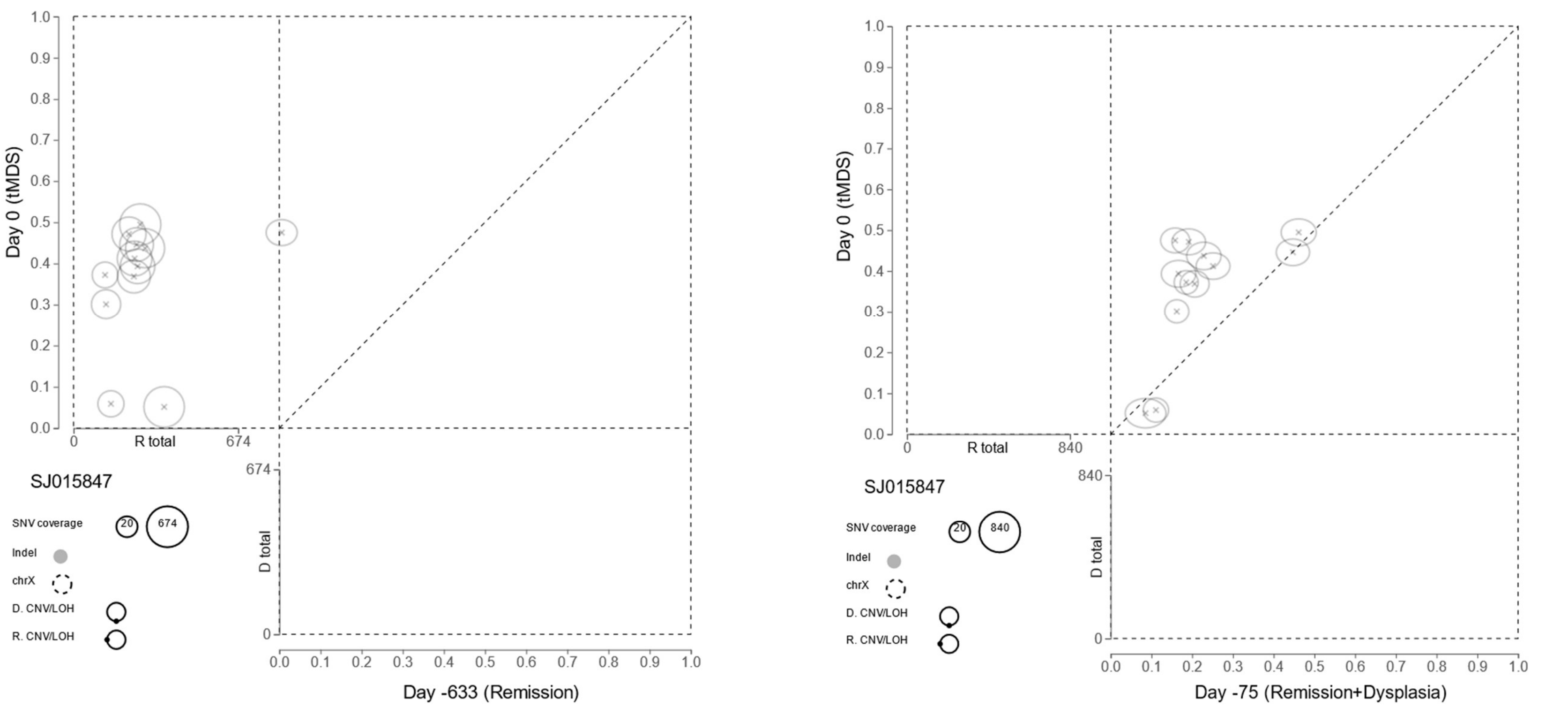
Day 0 (tMDS)
R total
0
674
SJ015847
SNV coverage
20
674
Indel
chrX
D. CNV/LOH
R. CNV/LOH
D total
Day -633 (Remission)
Day 0 (tMDS)
R total
0
840
SJ015847
SNV coverage
20
840
Indel
chrX
D. CNV/LOH
R. CNV/LOH
D total
Day -75 (Remission+Dysplasia)

f

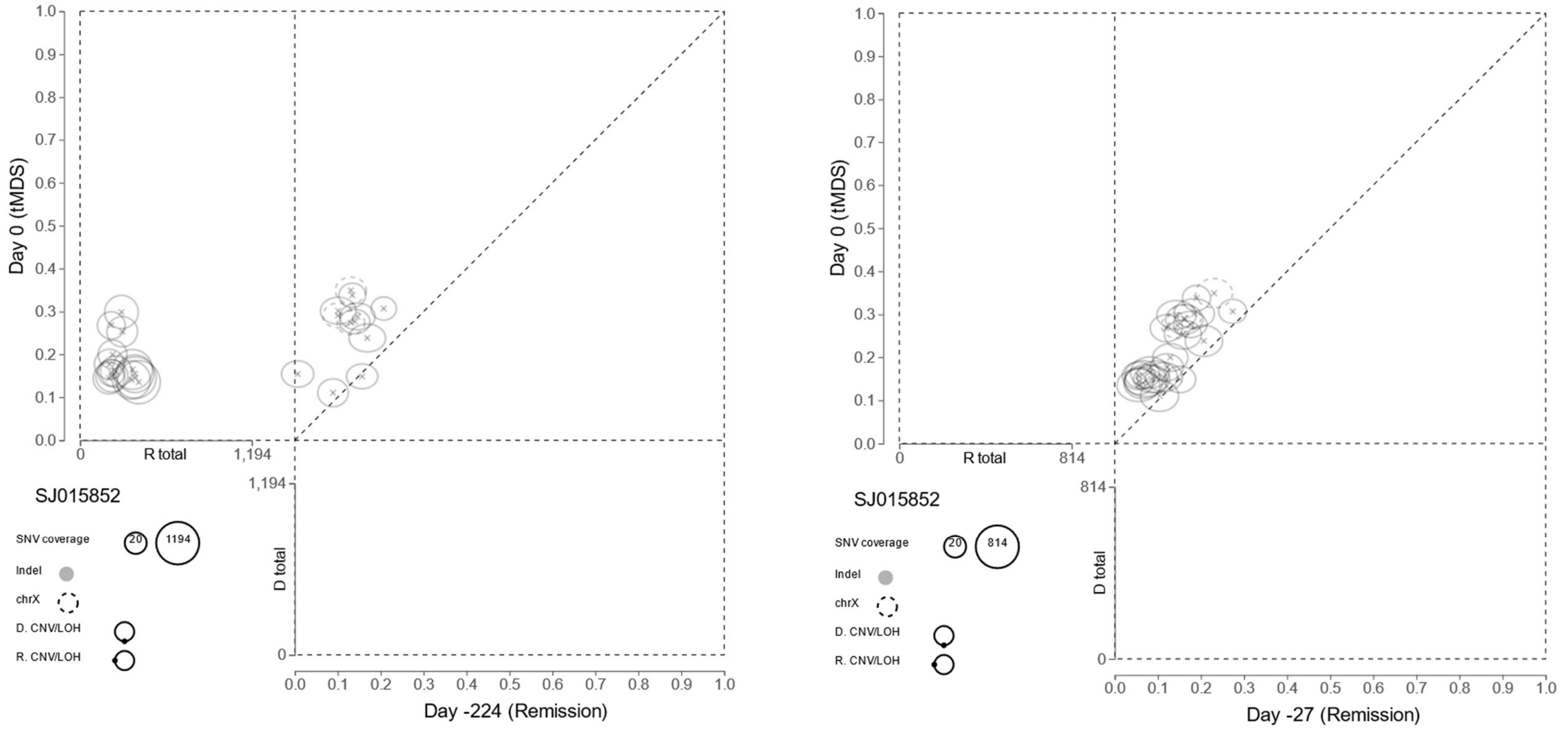

Day 0 (tMDS)
R total
0
1,194
SJ015852
SNV coverage
20
1194
Indel
chrX
D. CNV/LOH
R. CNV/LOH
D total
Day -224 (Remission)
Day 0 (tMDS)
R total
0
814
SJ015852
SNV coverage
20
814
Indel
chrX
D. CNV/LOH
R. CNV/LOH
D total
Day -27 (Remission)

g

h

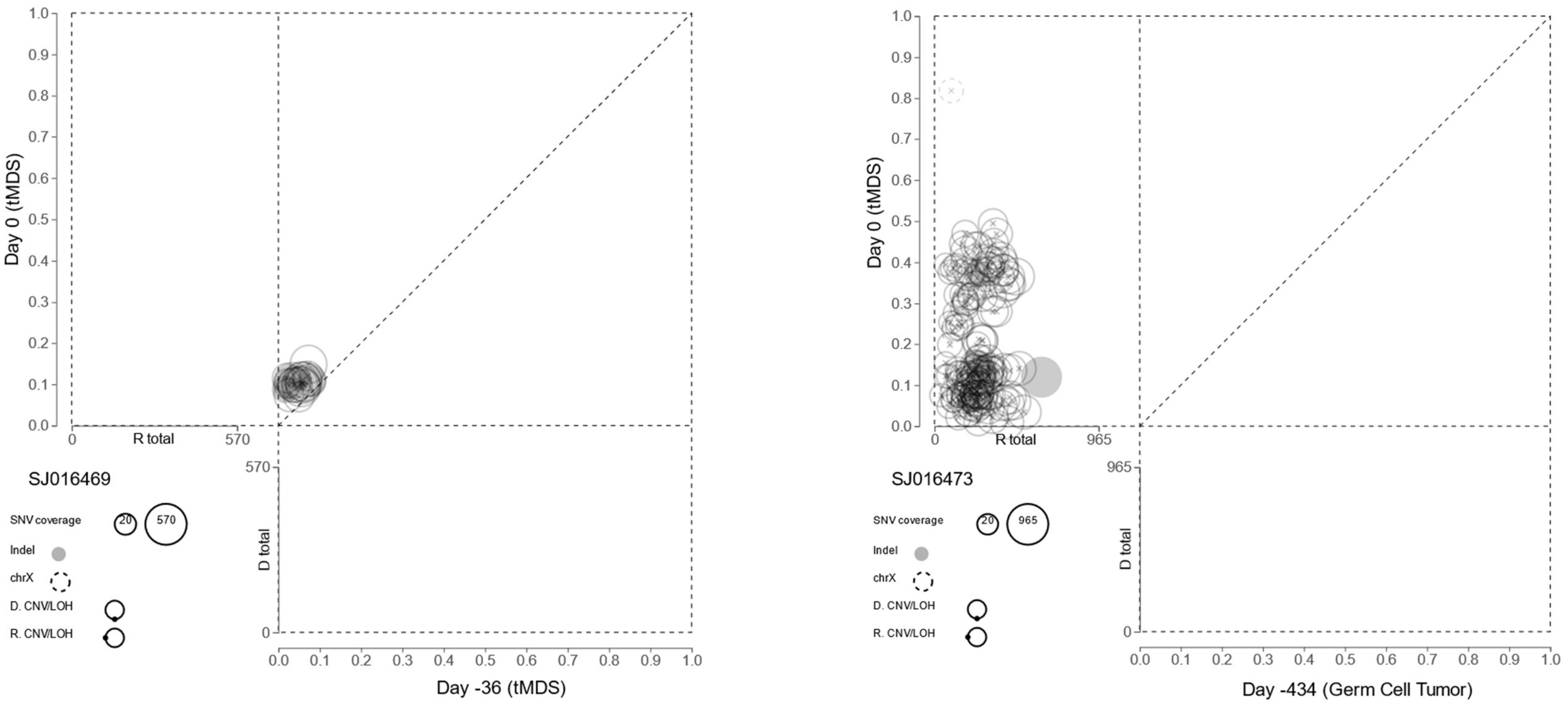

Day 0 (tMDS)
R total
0
570
SJ016469
SNV coverage
20
570
Indel
chrX
D. CNV/LOH
R. CNV/LOH
D total
Day -36 (tMDS)
Day 0 (tMDS)
R total
965
SJ016473
SNV coverage
20
965
Indel
chrX
D. CNV/LOH
R. CNV/LOH
D total
Day -434 (Germ Cell Tumor)

i

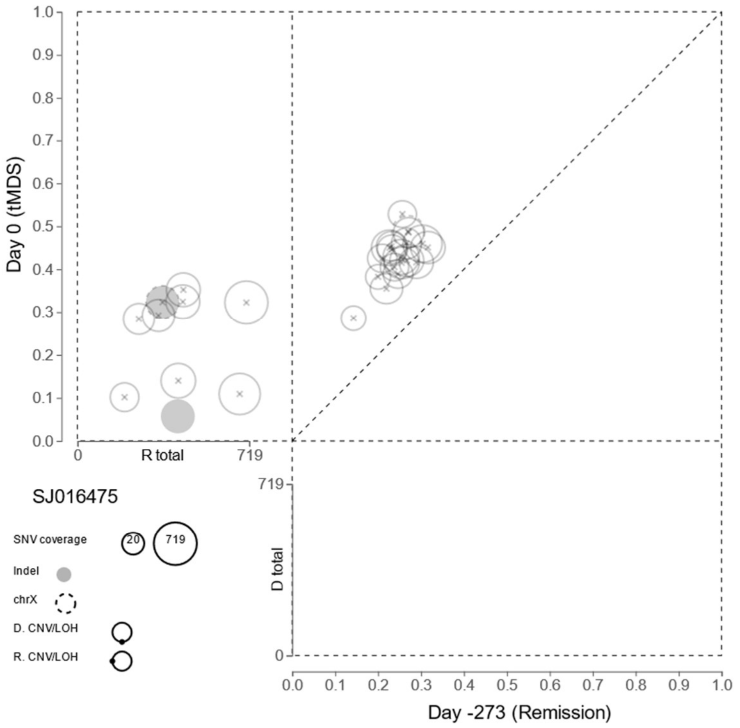

1.0
0.9
0.8
0.7
0.6
0.5
0.4
0.3
0.2
0.1
0.0
Day 0 (tMDS)
0
R total
719
SJ016475
SNV coverage
20
719
Indel
chrX
D. CNV/LOH
R. CNV/LOH
719
D total
0
0.0 0.1 0.2 0.3 0.4 0.5 0.6 0.7 0.8 0.9 1.0
Day -273 (Remission)

j

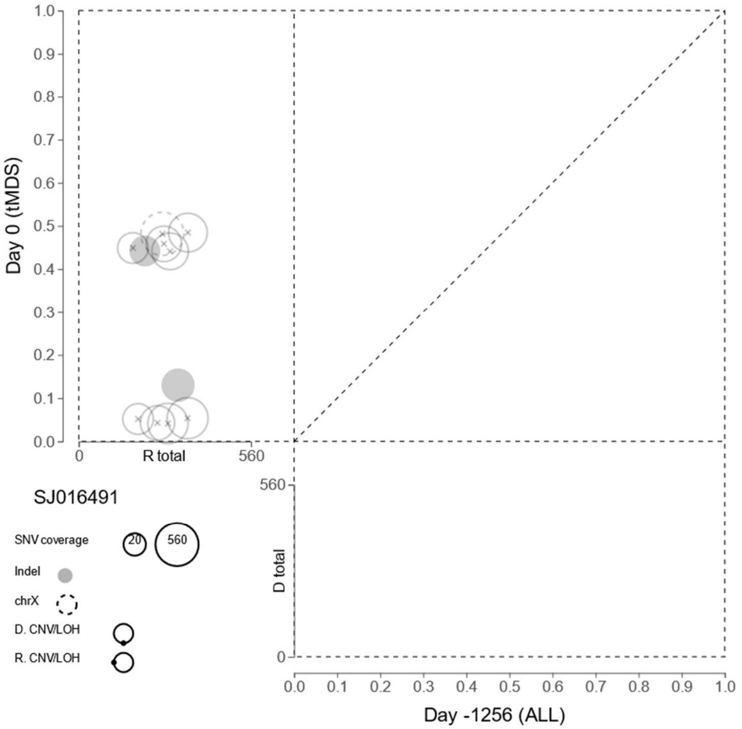

1.0
0.9
0.8
0.7
0.6
0.5
0.4
0.3
0.2
0.1
0.0
Day 0 (tMDS)
0
R total
560
SJ016491
SNV coverage
20
560
Indel
chrX
D. CNV/LOH
R. CNV/LOH
560
D total
0
0.0 0.1 0.2 0.3 0.4 0.5 0.6 0.7 0.8 0.9 1.0
Day -1256 (ALL)

k

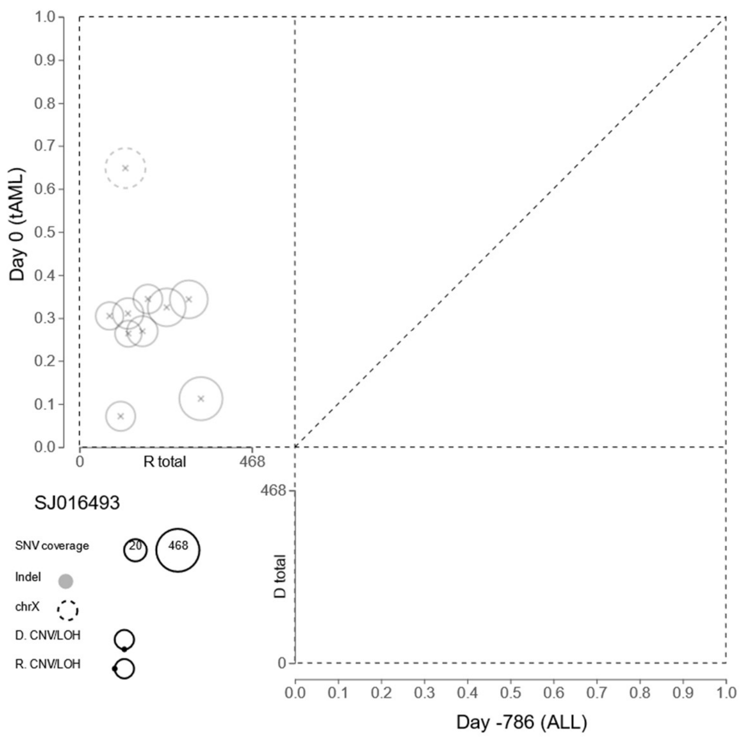

Day 0 (tAML)
R total
SJ016493
SNV coverage
20
468
Indel
chrX
D. CNV/LOH
R. CNV/LOH
D total
Day -786 (ALL)

l

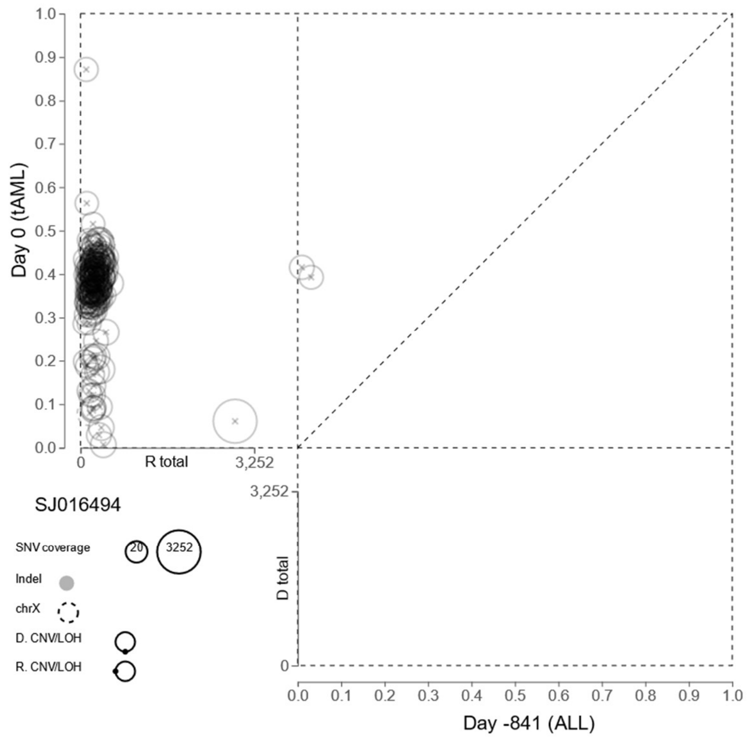

Day 0 (tAML)
R total
3,252
SJ016494
SNV coverage
20
3252
Indel
chrX
D. CNV/LOH
R. CNV/LOH
D total
Day -841 (ALL)

m

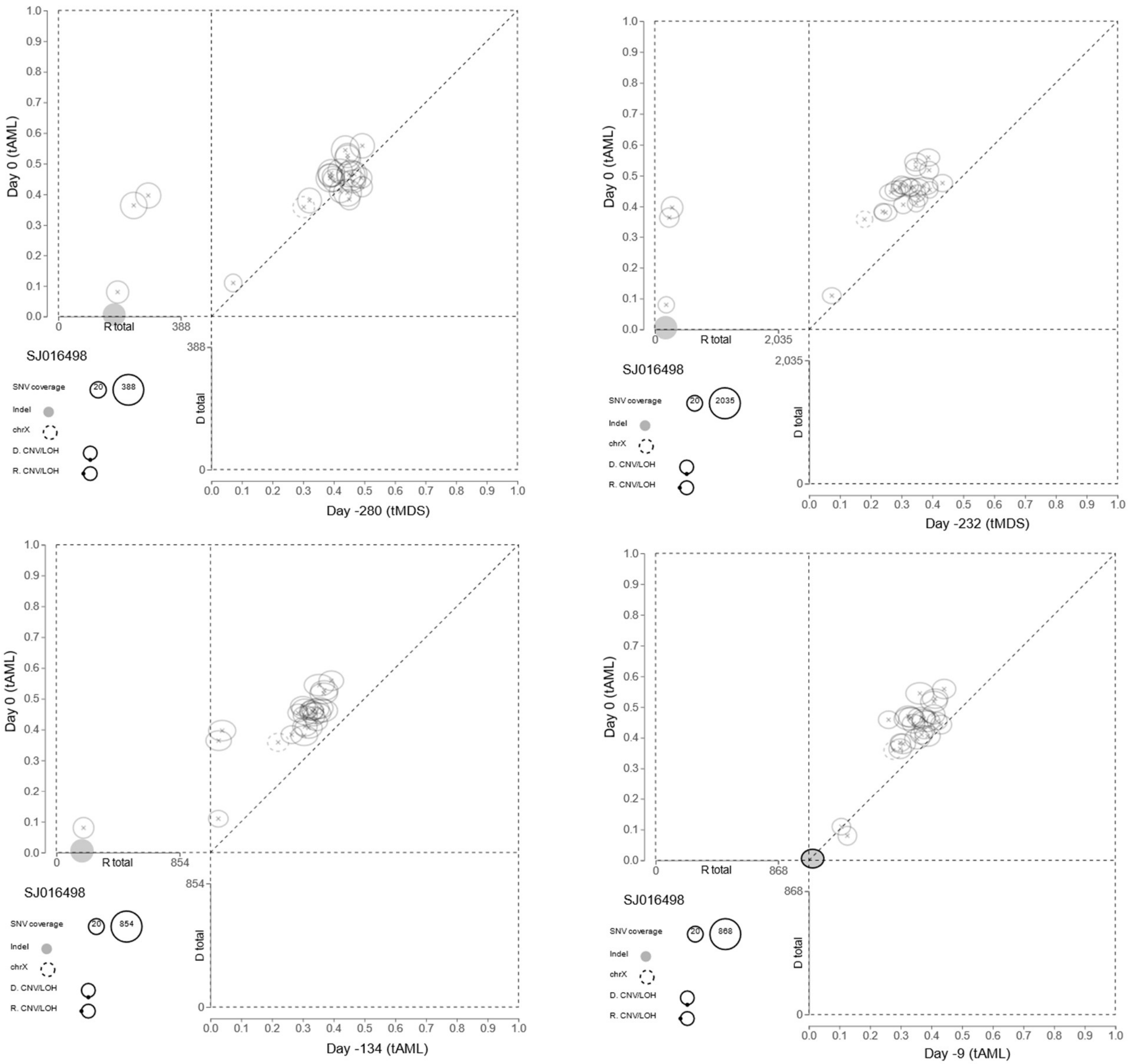

Day 0 (tAML)
R total
D total
SJ016498
SNV coverage
Indel
chrX
D. CNV/LOH
R. CNV/LOH
Day -280 (tMDS)
Day -232 (tMDS)
Day -134 (tAML)
Day -9 (tAML)

n

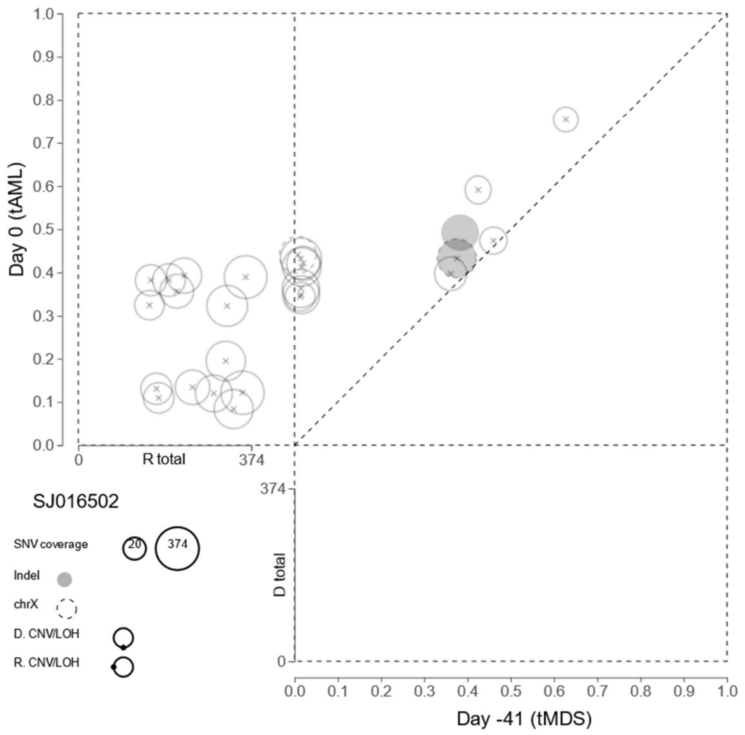

1.0
0.9
0.8
0.7
0.6
0.5
0.4
0.3
0.2
0.1
0.0
Day 0 (tAML)
0
R total
374
SJ016502
SNV coverage
20
374
Indel
chrX
D. CNV/LOH
R. CNV/LOH
374
D total
0
0.0 0.1 0.2 0.3 0.4 0.5 0.6 0.7 0.8 0.9 1.0
Day -41 (tMDS)

o

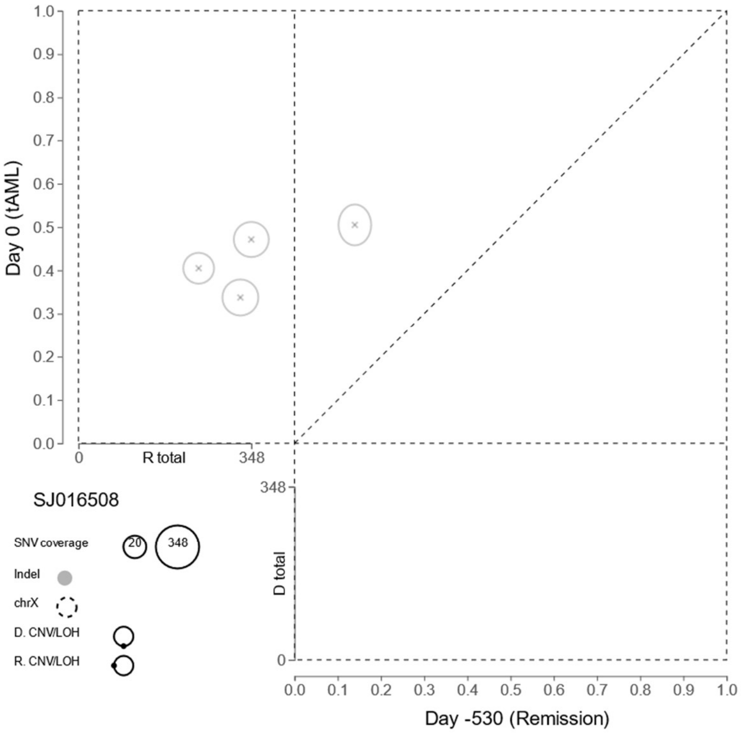

1.0
0.9
0.8
0.7
0.6
0.5
0.4
0.3
0.2
0.1
0.0
Day 0 (tAML)
0
R total
348
SJ016508
SNV coverage
20
348
Indel
chrX
D. CNV/LOH
R. CNV/LOH
348
D total
0
0.0 0.1 0.2 0.3 0.4 0.5 0.6 0.7 0.8 0.9 1.0
Day -530 (Remission)

p

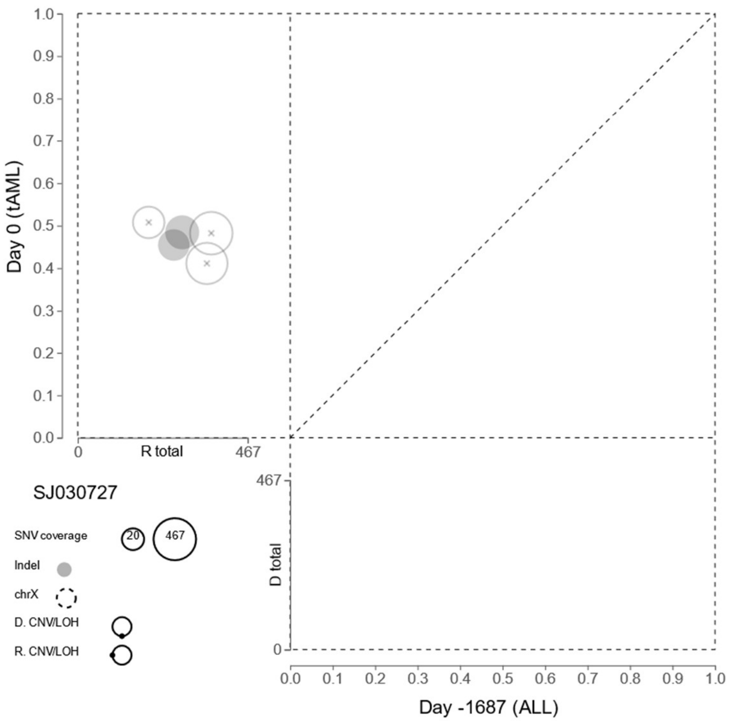

Day 0 (tAML)
1.0
0.9
0.8
0.7
0.6
0.5
0.4
0.3
0.2
0.1
0.0
0
R total
467
SJ030727
SNV coverage
20
467
Indel
chrX
D. CNV/LOH
R. CNV/LOH
467
D total
0
0.0 0.1 0.2 0.3 0.4 0.5 0.6 0.7 0.8 0.9 1.0
Day -1687 (ALL)

q

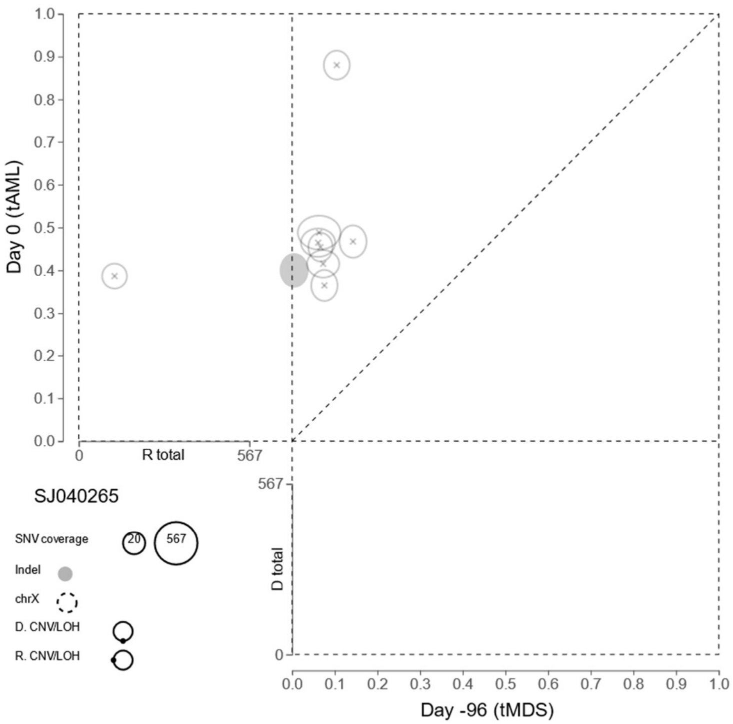

Day 0 (tAML)
1.0
0.9
0.8
0.7
0.6
0.5
0.4
0.3
0.2
0.1
0.0
0
R total
567
SJ040265
SNV coverage
20
567
Indel
chrX
D. CNV/LOH
R. CNV/LOH
567
D total
0
0.0 0.1 0.2 0.3 0.4 0.5 0.6 0.7 0.8 0.9 1.0
Day -96 (tMDS)

r

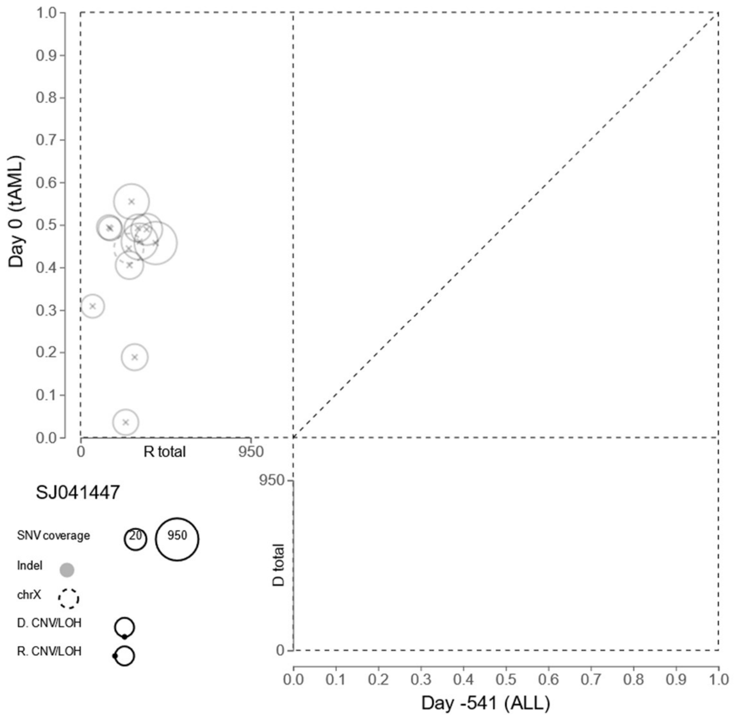

1.0
0.9
0.8
0.7
0.6
0.5
0.4
0.3
0.2
0.1
0.0
Day 0 (tAML)
0
R total
950
950
0
D total
SJ041447
SNV coverage
20
950
Indel
chrX
D. CNV/LOH
R. CNV/LOH
0.0 0.1 0.2 0.3 0.4 0.5 0.6 0.7 0.8 0.9 1.0
Day -541 (ALL)

s

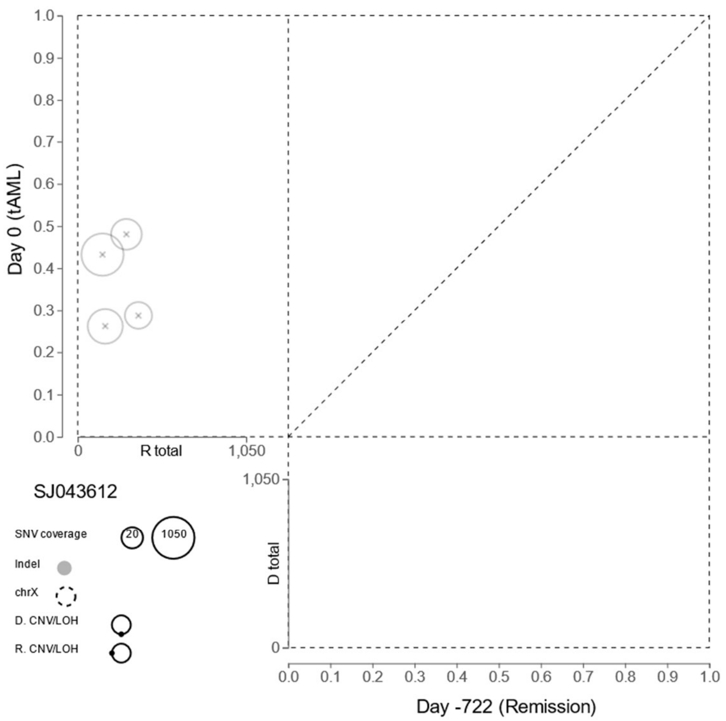

1.0
0.9
0.8
0.7
0.6
0.5
0.4
0.3
0.2
0.1
0.0
Day 0 (tAML)
0
R total
1,050
1,050
0
D total
SJ043612
SNV coverage
20
1050
Indel
chrX
D. CNV/LOH
R. CNV/LOH
0.0 0.1 0.2 0.3 0.4 0.5 0.6 0.7 0.8 0.9 1.0
Day -722 (Remission)

t

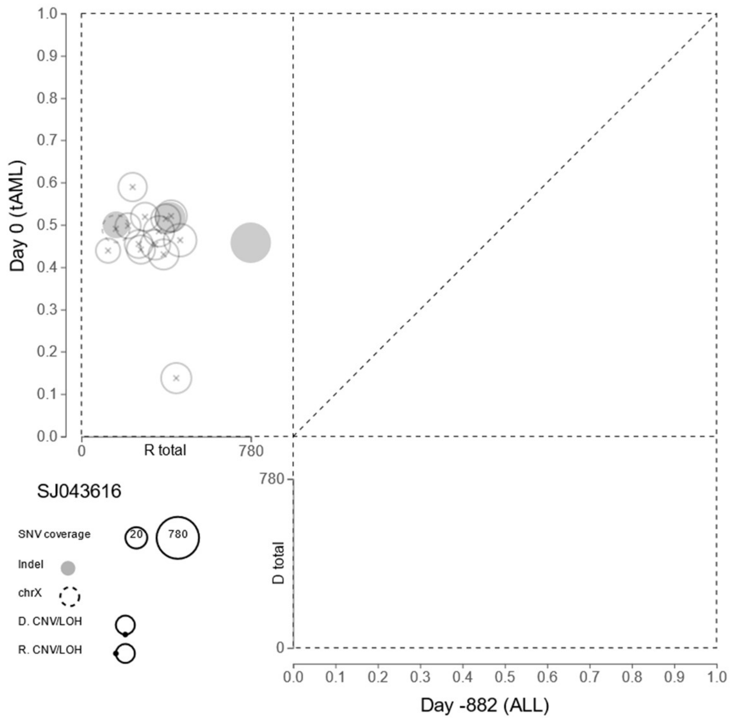

u

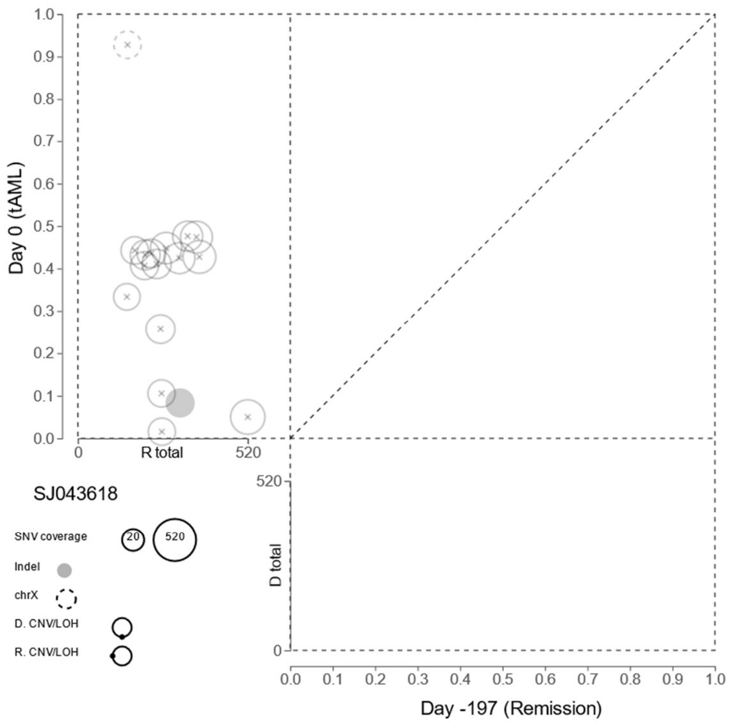

Supplementary Figure 16. a-u) 2-dimensional plots of the cases with only a single prior time point relative to the tMN, or cases with tAML progression from tMDS. These cases did not have suitable data to generate a river plot.

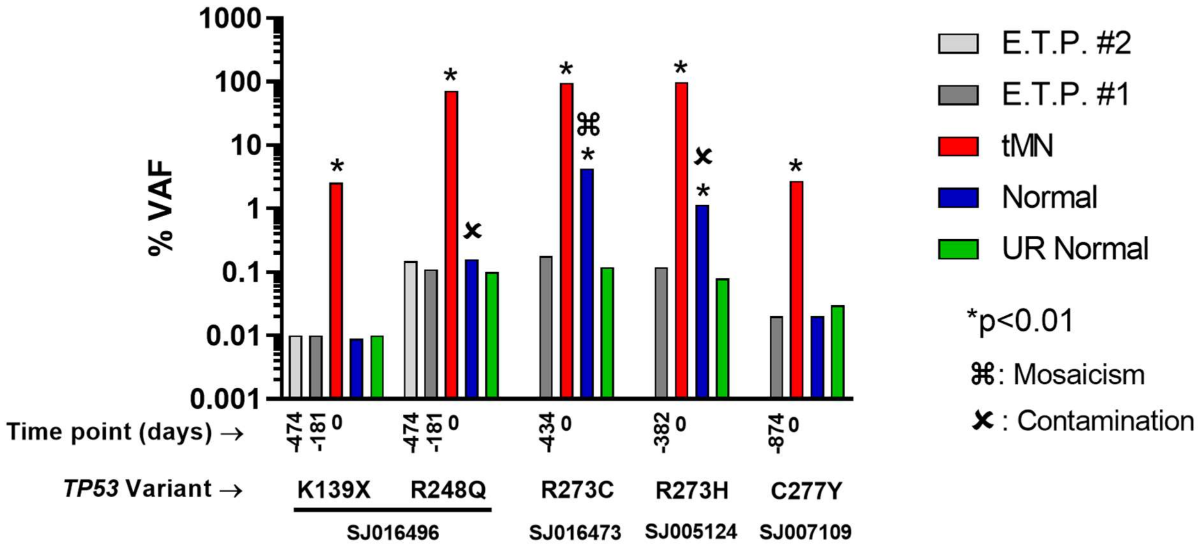

Supplementary Figure 17. Bar graph showing *TP53* variant VAFs at timepoints prior to tMN compared to the normal control (sorted lymphocytes) and an unrelated (UR) normal, also sorted lymphocytes. Statistical comparison was made versus the unrelated control by a Bonferroni corrected Fisher's exact test. Read counts used for these tests are listed in Supplementary Data 22. These data show that the *TP53* variants found in the tMN were not present in earlier time points (E.T.P). See exact number of days prior to tMN under each individual time point. The significant difference present between the normal and unrelated normal in SJ016473 and SJ005124 is most likely due to mosaicism or tumor in normal contamination, respectively.

a

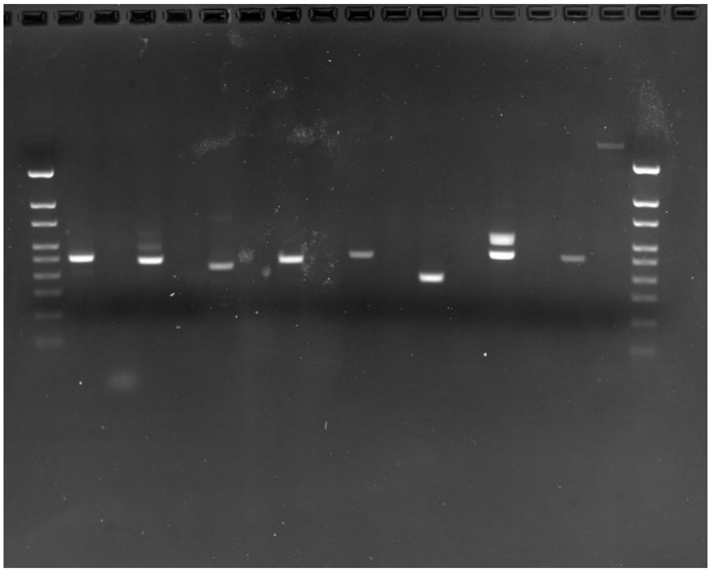

b

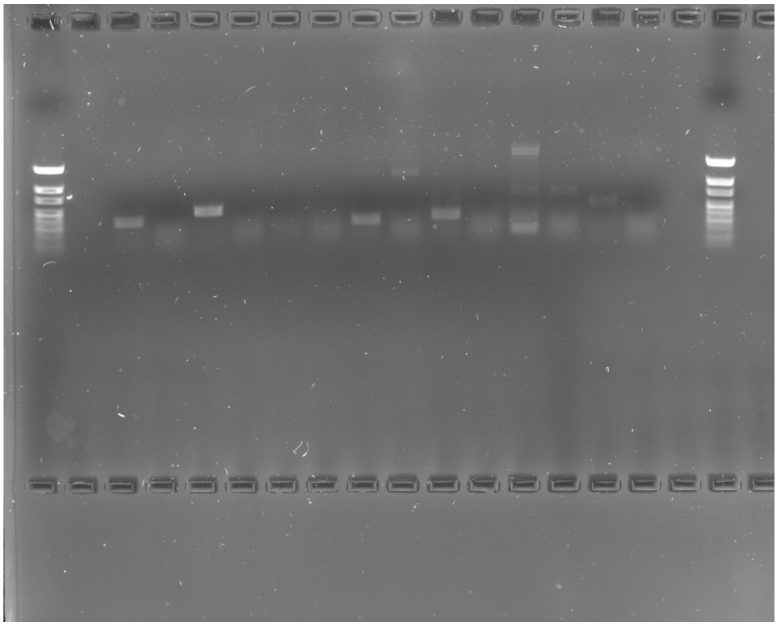

Supplementary Figure 18. Uncropped gel images of top (a) and bottom (b) images presented in Supplementary Figure 9.

REFERENCES:

1. Schwartz, J.R. *et al.* The genomic landscape of pediatric myelodysplastic syndromes. *Nature Communications* **8**, 1557 (2017).
2. Faber, Z.J. *et al.* The genomic landscape of core-binding factor acute myeloid leukemias. *Nat Genet* **48**, 1551-1556 (2016).
3. SEER*Explorer: An interactive website for SEER cancer statistics. (Surveillance Research Program, National Cancer Institute, 2020).
4. Shirts, B.H. *et al.* Using Somatic Mutations from Tumors to Classify Variants in Mismatch Repair Genes. *Am J Hum Genet* **103**, 19-29 (2018).
5. Li, B. *et al.* Therapy-induced mutations drive the genomic landscape of relapsed acute lymphoblastic leukemia. *Blood* **135**, 41-55 (2020).
6. Whyte, W.A. *et al.* Master transcription factors and mediator establish super-enhancers at key cell identity genes. *Cell* **153**, 307-19 (2013).
7. Zhang, X. *et al.* Large DNA Methylation Nadirs Anchor Chromatin Loops Maintaining Hematopoietic Stem Cell Identity. *Mol Cell* **78**, 506-521 e6 (2020).
8. Corces, M.R. *et al.* Lineage-specific and single-cell chromatin accessibility charts human hematopoiesis and leukemia evolution. *Nat Genet* **48**, 1193-203 (2016).
