# Supplementary material for: The acquisition of molecular drivers in pediatric therapy-related myeloid neoplasms
Source: Nat Commun. 2021 Feb 12;12:985. doi: 10.1038/s41467-021-21255-8 (PMC7880998; doi:10.1038/s41467-021-21255-8)
Supplement: Supplementary file 3 — Description of Additional Supplementary Files [file 41467_2021_21255_MOESM3_ESM.pdf]

Description of Additional Supplementary Files

File Name: Supplementary Data 1
Description: Clinical Data of the pediatric tMN cohort

File Name: Supplementary Data 2
Description: Rank-sum p-values for age for each molecular group, this data complements that of Supplementary Figure 1a & b.

File Name: Supplementary Data 3
Description: Rank-sum p-values for time-to-tMN for each molecular group, this data complements that of Supplementary Figure 1c & d.

File Name: Supplementary Data 4
Description: Complete list of somatic variants present in tumor/normal sub-cohort (n=62).

File Name: Supplementary Data 5
Description: Gene list for Twist panel used for variant validation via targeted sequencing.

File Name: Supplementary Data 6
Description: WGS QC data

File Name: Supplementary Data 7
Description: WGS QC data

File Name: Supplementary Data 8
Description: Targeted sequencing (TWIST) QC data

File Name: Supplementary Data 9
Description: Listing of DNA repair gene mutations present in the 4 cases with a hypermutated phenotype. Complements that of Supplementary Figure 2a.

File Name: Supplementary Data 10
Description: GRIN analysis

File Name: Supplementary Data 11
Description: Gene list categorized into functional class used for preparing Figure 1c.

File Name: Supplementary Data 12
Description: Complete list of germline variants present in the pediatric tMN cohort.

File Name: Supplementary Data 13
Description: Clinical & therapy data for patient's with P/LP germline variants

File Name: Supplementary Data 14
Description: Gene list used for germline variant determination.

File Name: Supplementary Data 15
Description: List of germline variants determined to be mosaic.

File Name: Supplementary Data 16
Description: Complete list of copy number alterations found in the tumor/normal sub-cohort (n=62).

File Name: Supplementary Data 17
Description: Type and dose of chemotherapy agents used for treatment of the primary malignancy for each case.

File Name: Supplementary Data 18
Description: Complete list of structural variants identified via RNASeq in the tumor/normal sub-cohort (n=62).

File Name: Supplementary Data 19
Description: RNASeq QC data

File Name: Supplementary Data 20
Description: Listing of primers used for RT-PCR fusion validation. This data complements Supplementary Figure 9.

File Name: Supplementary Data 21
Description: Read counts of all variants at each time point cases used to describe clonal evolution.

File Name: Supplementary Data 22
Description: Ultra-deep amplicon sequencing of TP53 in cases with TP53 variants and the determination of tumor in normal contamination or mosaicism. This data complements Supplementary Figure 17.
