# Supplementary material for: The acquisition of molecular drivers in pediatric therapy-related myeloid neoplasms
Source: Nat Commun. 2021 Feb 12;12:985. doi: 10.1038/s41467-021-21255-8 (PMC7880998; doi:10.1038/s41467-021-21255-8)
Supplement: Supplementary file 26 — Reporting Summary [file 41467_2021_21255_MOESM26_ESM.pdf]

nature research

Corresponding author(s): Jeffery M. Klco, Xiaotu Ma, Tanja Gruber

Last updated by author(s): Jan 8, 2021

Reporting Summary

Nature Research wishes to improve the reproducibility of the work that we publish. This form provides structure for consistency and transparency in reporting. For further information on Nature Research policies, see Authors & Referees and the Editorial Policy Checklist.

Statistics

For all statistical analyses, confirm that the following items are present in the figure legend, table legend, main text, or Methods section.

n/a	Confirmed	
☐	☒	The exact sample size (*n*) for each experimental group/condition, given as a discrete number and unit of measurement
☐	☒	A statement on whether measurements were taken from distinct samples or whether the same sample was measured repeatedly
☐	☒	The statistical test(s) used AND whether they are one- or two-sided<br>*Only common tests should be described solely by name; describe more complex techniques in the Methods section.*
☐	☒	A description of all covariates tested
☐	☒	A description of any assumptions or corrections, such as tests of normality and adjustment for multiple comparisons
☐	☒	A full description of the statistical parameters including central tendency (e.g. means) or other basic estimates (e.g. regression coefficient) AND variation (e.g. standard deviation) or associated estimates of uncertainty (e.g. confidence intervals)
☐	☒	For null hypothesis testing, the test statistic (e.g. *F*, *t*, *r*) with confidence intervals, effect sizes, degrees of freedom and *P* value noted<br>*Give P values as exact values whenever suitable.*
☒	☐	For Bayesian analysis, information on the choice of priors and Markov chain Monte Carlo settings
☒	☐	For hierarchical and complex designs, identification of the appropriate level for tests and full reporting of outcomes
☒	☐	Estimates of effect sizes (e.g. Cohen's *d*, Pearson's *r*), indicating how they were calculated

Our web collection on statistics for biologists contains articles on many of the points above.

Software and code

Policy information about availability of computer code

Data collection	No custom software was used in this study
Data analysis	Software used: BWA (WGS: v0.7.15-r1140; WES: v0.5.9-r26-dev & v0.7.12-r1039), Picard tools 1.65 (broadinstitute.github.io/picard/), Bambino, CREST (v1.0), StrongARM pipeline, CICERO (v0.3.0), Chimerascan (v0.4.5), BWA (v0.7.12) MEM algorithm, Rsamtools (v1.30.0), VarScan2 (v2.3.5), CONSERTING, Samtools (v1.1 & 1.2), DNAcopy R package, MutSigCV, Trim Galore (v0.4.4), biobambam2 (2.0.87), macs2 (v2.1.1)

For manuscripts utilizing custom algorithms or software that are central to the research but not yet described in published literature, software must be made available to editors/reviewers. We strongly encourage code deposition in a community repository (e.g. GitHub). See the Nature Research guidelines for submitting code & software for further information.

Data

Policy information about availability of data

All manuscripts must include a data availability statement. This statement should provide the following information, where applicable:
- Accession codes, unique identifiers, or web links for publicly available datasets
- A list of figures that have associated raw data
- A description of any restrictions on data availability

Genomic data have been deposited in the European Genome-Phenome Archive (EGA), which is hosted by the European Bioinformatics Institute (EBI), under accession EGAS00001004850 [https://www.ebi.ac.uk/ega/studies/EGAS00001004850] and St. Jude Cloud [https://pecan.stjude.cloud/permalink/tMN]. Other publicly available datasets used for CD34+ cell super-enchancer analysis are deposited in Gene Expression Omnibus (GEO) [https://www.ncbi.nlm.nih.gov/geo/] under the accession numbers GSE104579 [https://www.ncbi.nlm.nih.gov/geo/query/acc.cgi?acc=GSE104579], GSM772885 [https://www.ncbi.nlm.nih.gov/geo/query/acc.cgi?acc=GSM772885], and GSE74912 [https://www.ncbi.nlm.nih.gov/geo/query/acc.cgi]. Also used: NHLBI GO Exome Sequencing Project [http://evs.gs.washington.edu/EVS/]; 1000 genomes [http://www.internationalgenome.org]; ExAC non-TCGA version [http://exac.broadinstitute.org/], and GRCh37/hg19 human genome assembly

Field-specific reporting

Please select the one below that is the best fit for your research. If you are not sure, read the appropriate sections before making your selection.

☒ Life sciences ☐ Behavioural & social sciences ☐ Ecological, evolutionary & environmental sciences

For a reference copy of the document with all sections, see nature.com/documents/nr-reporting-summary-flat.pdf

Life sciences study design

All studies must disclose on these points even when the disclosure is negative.

Sample size	No sample size calculation was performed. Those tMN patients with appropriate informed consent and available material in the St. Jude Children's Research Hospital Tissue Bank were included in this study. Pediatric tMN is a rare disorder accounting for ~1% of pediatric patients treated for primary malignancies. The sample size used reflects tMN all available cases with material banked at our single institution with appropriate consent. This study provides a sampling of pediatric tMN from a single institution over approximately 2 decades.
Data exclusions	There are no data exclusions.
Replication	Given the nature of using banked patient material from diseases with very poor outcomes it was impossible to single each case and each timepoint more than once. Therefore, replicates were not completed for WGS, WES, or RNASeq, though orthogonal methods for confirming findings such has immunohistochemistry and RNASeq expression data were use to confirm a finding. Further, a selected panel of recurrently mutated genes in MDS was used for capture based sequencings (TWIST) to validate findings from WGS and WES.
Randomization	There was no randomization, as this study was not comparing any experimental groups, but rather characterizing one group.
Blinding	Blinding was not relevant to our study given that there were no experimental groups other than the whole cohort (pediatric tMN) being characterized.

Reporting for specific materials, systems and methods

We require information from authors about some types of materials, experimental systems and methods used in many studies. Here, indicate whether each material, system or method listed is relevant to your study. If you are not sure if a list item applies to your research, read the appropriate section before selecting a response.

Materials & experimental systems

n/a	Involved in the study	
☐	☒	Antibodies
☐	☒	Eukaryotic cell lines
☒	☐	Palaeontology
☒	☐	Animals and other organisms
☐	☒	Human research participants
☒	☐	Clinical data

Methods

n/a	Involved in the study	
☒	☐	ChIP-seq
☒	☐	Flow cytometry
☒	☐	MRI-based neuroimaging

Antibodies

Antibodies used	MECOM IHC antibody: C50E12, Cell Signaling Technology, dilution = 1:500
Validation	Shown to bind MECOM in multiple cell lines on the manufacturer's website (www.cellsignal.com), original citation is PMID: 29939287

Eukaryotic cell lines

Policy information about cell lines

Cell line source(s)	ATCC (cat.# CCL-243)
Authentication	Our K562 cell line has been karyotyped at SJCRH (10/20/2017), with a complex karyotype consistent with that previously reported.
Mycoplasma contamination	Our K562 cell line has not recently been tested for mycoplasma.
Commonly misidentified lines (See ICLAC register)	None listed on the ICLAC register

Human research participants

Policy information about studies involving human research participants

Population characteristics	All tMN patients with appropriate informed consent and with available stored material in the St. Jude Children's Research Hospital's Tissue Bank were included. The characteristics of the patient's used are listed in Supplementary Table 1 and Figure 1a. None of these were used as an experimental covariant at the outset of the study.
Recruitment	All tMN patients with appropriate informed consent and with available stored material in the St. Jude Children's Research Hospital's Tissue Bank were included. Therefore, potential self-selection bias may be present given that there is likely some pediatric tMN cases during the time period in question that did not consent to having material banked. This self-selection bias should have negligible effects on any data presented in the manuscript. There was no recruitment of individuals for this study.
Ethics oversight	St. Jude Children's Research Hospital Institutional Review Board.

Note that full information on the approval of the study protocol must also be provided in the manuscript.
